# Lesion remyelinating activity of GSK239512 versus placebo in patients with relapsing-remitting multiple sclerosis: a randomised, single-blind, phase II study

**DOI:** 10.1007/s00415-016-8341-7

**Published:** 2016-11-25

**Authors:** Caryl J. Schwartzbach, Richard A. Grove, Robert Brown, Debra Tompson, Florian Then Bergh, Douglas L. Arnold

**Affiliations:** 1 GSK, Research Triangle Park, Raleigh-Durham, NC USA; 2 GSK, Stockley Park, Uxbridge, UK; 3 McGill University, Montreal, QC Canada; 4 GSK, Gunnels Wood Road, Stevenage, Hertfordshire UK; 5 University of Leipzig, Leipzig, Germany; 6 Department of Neurology, NeuroRx Research, Montreal, QC Canada

**Keywords:** Relapsing-remitting multiple sclerosis, Remyelination, GSK239512, Magnetic resonance imaging, Magnetisation transfer ratio

## Abstract

Histamine H_3_ receptor blockade may enhance lesion remyelination in multiple sclerosis (MS). The efficacy (using a magnetic resonance imaging marker of myelination, magnetisation transfer ratio [MTR]), safety and pharmacokinetics of GSK239512, a potent and brain penetrant H_3_ receptor antagonist/inverse agonist on lesion remyelination in relapsing-remitting MS (RRMS) were assessed. This was a phase II, randomised, parallel-group, placebo-controlled, double-blind (sponsor-unblinded), international, multicentre study (NCT01772199). Patients aged 18–50 with RRMS, receiving intramuscular interferon-β1a or glatiramer acetate, were randomised 1:1 to once-daily oral GSK239512 or placebo, up-titrated over 4–5 weeks to a maximum tolerable dose up to 80 µg and maintained until Week 48. The co-primary endpoints were mean changes in post-lesion MTR in gadolinium-enhanced (GdE) or Delta-MTR defined lesions from pre-lesion values. Adverse events (AE) and withdrawals were monitored. Of the 131 patients randomised, 114 patients completed the study (GSK239512, *n* = 51; placebo, *n* = 63) and 27 (GSK239512) and 28 (placebo) patients contributed lesions to the primary analysis. GSK239512 was associated with positive effect sizes of 0.344 [90% confidence interval (CI) 0.018, 0.671] and 0.243 (90% CI −0.112, 0.598) for adjusted mean changes in the normalised MTR for GdE and Delta-MTR lesions, respectively. The overall incidence of AEs was similar between GSK239512 and placebo during the treatment phase although some AEs including insomnia were more common with GSK239512, particularly during the titration period. A small but positive effect of GSK239512 on remyelination was observed. MTR assessment represents a promising method for detecting lesion remyelination in RRMS.

**Electronic supplementary material:**

The online version of this article (doi:10.1007/s00415-016-8341-7) contains supplementary material, which is available to authorized users.

## Background

Multiple sclerosis (MS) is a chronic progressive neurologic disorder, characterised by inflammatory-demyelinating lesions in the central nervous system (CNS) and associated axonal loss. Relapsing-remitting MS (RRMS) accounts for 85% of all incident cases of MS and is associated with episodes of CNS dysfunction, reflecting acute inflammatory focal demyelination and subsequent spontaneous recovery, in turn reflecting resolution of focal inflammation and remyelination, with a variable contribution of CNS plasticity [1]. Remyelination is a naturally occurring process but is rarely complete, and failure becomes more extensive with increased disease duration [2].

Decreased oligodendrocyte precursor cell (OPC) differentiation may play a role in remyelination failure [3]. The promotion of remyelination through increased OPC differentiation may provide functional recovery and prevent or reduce irreversible disability accumulation. OPC differentiation is controlled by a range of molecules and their receptors, including constitutively active histamine H_3_ receptors expressed by neurons and OPCs [4]. The importance of constitutive H3R activity in OPC differentiation is demonstrated by the ability of inverse agonists, which inhibit both ligand-independent and ligand-dependent receptor activation but not neutral antagonists of H3R, to promote OPC differentiation [4, 5].

GSK239512 is a potent, selective, orally bioavailable and brain penetrant H_3_ receptor antagonist/inverse agonist, originally investigated for the treatment of cognitive impairment in Alzheimer’s disease (AD) and schizophrenia [6–8]. Previous clinical studies have indicated that GSK239512 is well tolerated as a monotherapy when titrated up to 80 µg, resulting in >90% H_3_ receptor occupancy in the brain at trough plasma concentrations [6, 7, 9]. GSK239512 promotes OPC differentiation in vitro and enhances remyelination in the cuprizone mouse model of remyelination [4].

Magnetisation transfer ratio (MTR) imaging on magnetic resonance imaging (MRI) can be used to assess myelin content in vivo. MTR changes have been demonstrated to correlate with myelin content in animal models [10–13] and humans [14–17]. The aim of this study was to determine the efficacy of GSK239512 in terms of lesion remyelination in patients with RRMS, using changes in MTR as a marker of remyelination.

## Methods

### Study design

This was a phase II, randomised, parallel-group, placebo (adjunct)-controlled, double-blind (sponsor-unblinded) study conducted across 34 centres in eight countries (Ukraine, Spain, Germany, Bulgaria, Canada, UK, Czech Republic and Sweden). ClinicalTrials.gov, number NCT01772199; GSK study identifier, H3M116477.

The study was conducted in accordance with the International Conference on Harmonisation of Technical Requirements for Registration of Pharmaceuticals for Human Use, Good Clinical Practice (ICH-GCP) and the ethical principles outlined in the Declaration of Helsinki 2008 [18]. Ethics approval was obtained from respective countries’ Ethics Committees (Online resource 1). The study protocol is available online at http://www.gsk-clinicalstudyregister.com/files2/gsk-116477-protocol-redact.pdf.

### Participants

Inclusion criteria included age 18–50 years, MS diagnosis according to McDonald criteria [19], a relapsing-remitting course [20, 21], MS onset within 10 years of screening, stable disease-modifying treatment (DMT) with intramuscular interferon-β1a or glatiramer acetate for ≥1 year, recent disease activity [≥1 gadolinium-enhanced (GdE) lesion on MRI or reported relapse within the past year], and an Expanded Disability Status Scale (EDSS) [22] score of 1–4.5 at screening. Females were eligible if they were not pregnant, nursing, or of childbearing potential. Patients were excluded if they were unable to undergo regular MRI scans, had a history of severe and clinically significant CNS trauma, epilepsy, sleep disturbance, the presence or history of hallucinations, an uncontrolled medical condition, a significant electrocardiogram abnormality, or renal insufficiency. Online Resource 1 details full eligibility criteria. Patients remained on a stable DMT dose and regimen (either intramuscular interferon-β1a or glatiramer acetate) for the study duration. Patients provided written informed consent prior to any study-specific procedures.

### Randomisation and masking

Randomisation was performed centrally, using a computer-generated randomisation schedule created by the study statistician, stratified according to DMT. Randomisation numbers were allocated to patients by an interactive voice recognition system (IVRS; RAMOS). Drug bottles were numbered and assigned as per treatment assignment and tablets were of identical appearance. All patients, site staff and sponsor staff directly involved in interactions with sites remained blinded to treatment assignment throughout the study. At the time of the interim analysis the statistician was unblinded to treatment assignment. Additionally, two clinical team members (Investigative Lead and Early Development Lead) were unblinded to treatment level results but not individual patient treatment assignment.

### Procedures

Patients were randomised 1:1 to receive once-daily, oral GSK239512 or placebo for a treatment phase of up to 48-weeks (4- to 5-week titration period and maintenance period until Week 48). Patients started on GSK239512 10 µg or matching placebo and up-titrated weekly (20, 40 and 80 µg) to a maximum dose of 80 µg. If tolerability issues were reported, patients were either maintained at their current dose-level or down-titrated and/or the titration period extended by an additional week. The maximum tolerated dose during the titration period was continued during the maintenance period. One down-titration was permitted during the maintenance period if tolerability issues were reported. There was a minimum follow-up period of 2 weeks following end of treatment at Week 48 or early withdrawal.

### Outcomes

#### Co-primary endpoints

The co-primary endpoints were mean changes in MTR post-lesion compared with pre-lesion in newly developed lesions (during the conduct of the study) defined by either GdE or Delta-MTR [23]. New GdE lesions were defined as an area of increased signal intensity on a post-contrast T1-weighted MRI that was not due to normal structures and was not present on a previous scan. New Delta-MTR lesions were defined as regions that experience a decrease in MTR that is greater than the 99th percentile of the normal variation measured in white matter (WM) from one scan to the next and was not present on a previous scan. Further details on how these lesions were identified are included in the Online Resource 1. Both GdE or Delta-MTR lesions were included as previous studies have found that areas of Gd enhancement are not always co-localised with Delta-MTR changes indicating demyelination [24, 25]; additionally, Delta-MTR lesions can demonstrate larger changes in myelination/demyelination, so provide more statistical power to detect treatment effects [24, 25]. This study was not designed to determine a direct effect on lesion formation.

#### Secondary endpoints

Secondary MRI endpoints included assessment of adjusted mean changes from baseline in T2 lesion MTR, the number of new or enlarging T2 or GdE lesions, cumulative unique active lesions, new unenhancing T1 lesions, new GdE lesions evolving into chronic unenhancing T1 black holes, and total brain, WM and grey matter (GM) volumes. MRI scans (including conventional and MTR sequences as described in Online Resource 1) were performed at approximately 6-week intervals. Secondary clinical and patient-reported outcomes included occurrence of relapse, disability and functionality, cognitive impairment and health outcomes. During the titration period, clinical visits occurred approximately every 7 days and during the maintenance period approximately every 4 weeks. Relapse was assessed as per the criteria by Polman et al. [19] at each clinical visit, based on subject self-reporting. Disability and functionality were assessed using EDSS at baseline and every 12 weeks, cognitive impairment by a CogState battery at baseline and at Week 12, 24 and 48, and health outcomes by Multiple Sclerosis Quality of Life (MSQoL)-54 at baseline and at Week 48 [26]. Safety assessments included monitoring adverse events (AEs) and serious AEs (SAEs), clinical laboratory tests, vital sign assessments and suicidal ideation or behaviours using the IVRS version of the Columbia-Suicide Severity Rating Scale. Pharmacokinetics were assessed for plasma concentrations at 0.5, 2 and 6 h post-dose at Week 8, and trough concentrations at Weeks 4, 8, 24, 36 and 48.

### Statistical analysis

The intent-to-treat (ITT) population (all patients receiving ≥1 dose of study medication) was used for all efficacy and safety analyses. The co-primary endpoints were analysed using a mixed model for repeated measures [27]. The average change in MTR before and after new lesion formation was modelled separately for each lesion within each patient with at least two MRI scans pre- and post-lesion and an interval of at least 70 days from lesion formation to post-lesion scan, allowing for variation in effects between patients, between different lesions, within patients, and within lesions across time (Online Resource 1). The date of lesion identification is labelled as the reference MRI, and all visits are seen relative to that visit, described as relative MRIs. Statistical models were used to estimate treatment effect sizes of GSK239512 relative to placebo. Due to the exploratory nature of this study, no study endpoints were formally statistically powered and a sample size of approximately 100 patients was considered sufficient to estimate the effect size. Posterior probabilities were generated using Bayesian analyses using a non-informative prior distribution to calculate the posterior probability of the effect size being ≥0 based on data observed in the study, i.e. assessing potential effectiveness of the drug [28], and to provide an understanding of the probabilistic distribution of effect sizes for GSK23512 relative to placebo [29]. Full details on statistical analysis and sample size derivation can be found in Online Resource 1.

### Role of the funding source

This study was funded by GSK (H3M116477). Some authors were employees of GSK at the time of the study and were involved in study design, data collection, data analysis, data interpretation, and the writing of the report. All authors had full access to all the data in the study and had final responsibility for the decision to submit for publication.

## Results

**Fig. 1:**
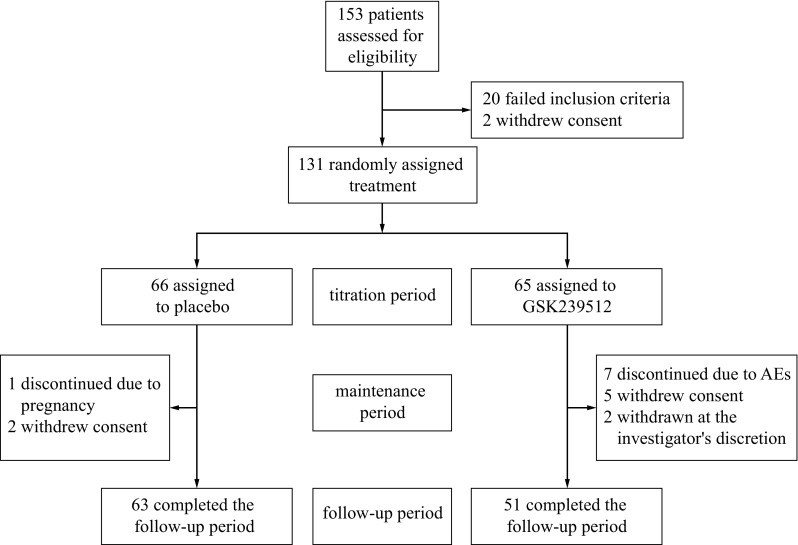
Summary of design and study entry. *AE* adverse event

Of the 153 patients screened across 35 sites between 1 February and 19 August, 2013, 131 were randomised at 34 sites (65 to GSK239512; 66 to placebo). In total, 22 patients failed screening: 20 did not meet inclusion or exclusion criteria and two withdrew consent. All randomised patients were included in the ITT population and completed the titration period. A total of 114 patients, 51 (78%) in the GSK239512 and 63 (95%) in the placebo groups completed both the treatment and follow-up phases. Reasons for withdrawal are shown in Fig. 1.

In total, 55 (85%) and 65 (98%) patients in the GSK239512 and placebo groups, respectively, were able to titrate to 80 µg by the end of the titration period. During the maintenance period, five patients down-titrated from 80 to 40 µg in the GSK239512 group. Overall, patient demographics and baseline characteristics in both treatment groups were as expected for the target population and were balanced between treatment groups (Table 1). The mean and median number of days since relapse was greater in the GSK239512 compared with the placebo group.

**Table 1:** Summary of MS medical history at screening (ITT population)

	Placebo (*N* = 66)	GSK239512 (*N* = 65)	Total (*N* = 131)
Age (years), mean (range)	36.2 (19–50)	36.4 (22–49)	36.3 (19–50)
Female, *n* (%)	41 (62)	42 (65)	83 (63)
Weight (kg), mean (range)	73.19 (48–128)	70.07 (48–109)	71.65 (48–128)
Body mass index (kg/m^2^), mean (range)	24.92 (16.0–40.4)	24.18 (16.9–36.7)	24.55 (16.0–40.4)
Caucasian, *n* (%)	66 (100)	65 (100)	131 (100)
Years since confirmation of diagnosis	5.12 (2.971)	4.74 (2.896)	4.93 (2.929)
Years since onset of symptoms	7.55 (3.920)	7.73 (4.383)	7.64 (4.141)
Receiving disease-modifying treatment, *n* (%)			
Intramuscular interferon-β1a	38 (58)	37 (57)	75 (57)
Glatiramer acetate	28 (42)	28 (43)	56 (43)
Total number of relapses	4.2 (2.84)	4.2 (2.82)	4.2 (2.82)
Number of relapses (last 12 months)	1.1 (0.49)	1.1 (0.55)	1.1 (0.52)
Number of relapses (last 24 months)	1.8 (1.04)	1.8 (1.02)	1.8 (1.02)
Days since last relapse^a^			
Mean (SD)	184.9 (180.77)	232.3 (265.77)	208.4 (227.33)
Median (range)	129 (7, 1243)	184 (7, 1597)	160 (7, 1597)
Number of scans with GdE lesions in last 12 months^b^, *n* (%)^c^	*n* = 28	*n* = 40	*n* = 68
0	13 (46)	22 (55)	35 (51)
1	14 (50)	18 (45)	32 (47)
2	1 (4)	0	1 (1)
T2 lesion MTR at screening MRI, mean (SD)	−0.036 (0.324)	−0.048 (0.385)	
Whole brain volume at screening MRI (cm^2^), mean (SD)	1486.3 (79.78)	1475.4 (68.87)	
EDSS scores at screening, median (min, max)	2.50 (1.0–4.5)	2.50 (1.0–4.5)	

Data shown represent mean (SD) unless otherwise specified

*EDDS* Expanded Disability Status Scale, *GdE* Gadolinium-enhanced, *ITT* intent-to-treat, *MRI* magnetic resonance imaging, *MS* multiple sclerosis, *MTR* magnetisation transfer ratio, *SD* standard deviation

^a^Days since last relapse are calculated based on the screening visit and date of relapse

^b^Subset of patients that had an MRI for GdE lesions in the 12 months before study enrolment

^c^Proportion of patients with scan data

**Fig. 2:**
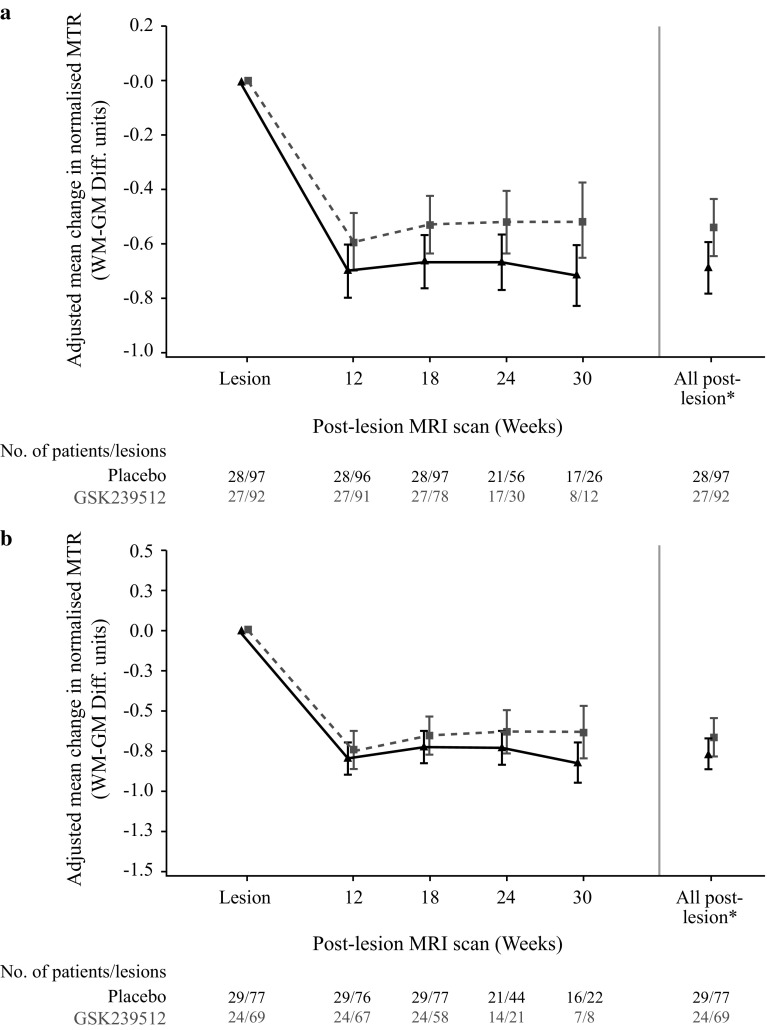
Adjusted mean change in MTR value (90% CI) for **a** GdE lesions and **b** Delta-MTR lesions (ITT population). *CI* confidence interval, *GdE* Gadolinium-enhanced, *GM* grey matter, *ITT* intent-to-treat, *MRI* magnetic resonance imaging, *MTR* magnetisation transfer ratio, *WM* white matter. MRI scanning was performed at approximately 6-week intervals post-reference lesion; GSK293512 is represented by a *hashed line* and placebo by a *solid line*; the *asterisk* ‘All post-lesion’ data points represent the mean values for the adjusted mean change in normalised MTR at 12, 18, 24 and 30 weeks

Overall, 92 lesions in 27 patients in the GSK239512 group and 97 lesions in 28 patients in the placebo group contributed to the primary analysis of GdE lesions. For Delta-MTR lesions, 69 lesions in 24 patients in the GSK239512 group and 77 lesions in 29 patients in the placebo group contributed to the primary analysis. The analysis excluded all MRI data 70 days pre- and post-reference lesion, and indicated that effect sizes for the change in GdE and Delta–MTR lesions were 0.344 (90% CI 0.018, 0.671) and 0.243 (90% CI −0.112, 0.598), respectively. The adjusted mean change in normalised MTR (difference between normal WM and GM) for GdE lesions was 0.149 (90% CI 0.008, 0.289; Fig. 2a) and Delta-MTR lesions was 0.105 (90% CI −0.049, 0.257; Fig. 2b) favouring GSK239512 over placebo (Table 2). These values are in calibrated MTR units [30] and are interpretable as improved MTR recovery corresponding to 15% (GdE) and 10% (Delta-MTR) of the difference between normal GM and WM. In the Bayesian analysis, estimated probabilities were 0.955 and 0.877 that the effect size for GSK239512 relative to placebo was greater than 0, for the change in post- versus pre-lesion for GdE and Delta-MTR lesions, respectively (Online Resource 2).

**Table 2:** Analysis of mean change in MTR value for all eligible GdE lesions and Delta-MTR lesions (ITT population)

Combined post-lesion MRIs	Treatment	Patients, *N*	Lesions, *n*	Adjusted mean change for normalised MTR^a^ (WM-GM diff units)	Difference in adjusted mean change (90% CI)	ES^b^ (90% CI)	Posterior probability^c^
GdE lesion	Placebo	28	97	−0.689	0.149 (0.008, 0.289)	0.344 (0.018, 0.671)	0.955
	GSK239512	27	92	−0.541			
Delta-MTR lesion	Placebo	29	77	−0.769	0.105 (−0.048, 0.257)	0.243 (−0.112, 0.598)	0.877
	GSK239512	24	69	−0.665			

*CI* confidence interval, *ES* effect size, *GdE* Gadolinium-enhanced, *GM* grey matter, *ITT* intent-to-treat, *MRI* magnetic resonance imaging, *MTR* magnetisation transfer ratio, *WM* white matter

^a^Adjusted mean change for normalised MTR is the post-lesion MTR value change from average pre-lesion MTR value

^b^ES is defined as the treatment difference divided by the standard deviation of the treatment difference averaged across relative MRIs

^c^Posterior probability is the conditional probability that the ES > 0 given the data observed in the study assuming a non-informative prior distribution

At Week 48, there was an increase in lesion development observed trending in favour of placebo (Table 3); post hoc investigations identified an interaction between treatment group and GdE lesion presence on the screening MRI, indicating that patients in the small subgroup with no GdE lesions at the screening MRI had more than double the number of new lesions, on average, during the treatment period on GSK239512 compared with placebo (Online Resource 3). Over the duration of the study, observed changes in brain volume were small. At Week 48, no difference in evolution of whole brain, WM or GM volume was observed between treatment groups (Online Resource 4).

**Table 3:** Analysis of secondary MRI assessments at Week 48 (ITT population)

	Patients, *n*	Adjusted mean change from screening	Difference in adjusted mean change^a^ (90% CI)	ES^b^ (90% CI)	Posterior probability
T2 lesion MTR at Week 48					
Placebo	57	0.002	−0.022 (−0.052, 0.009)	−0.246 (−0.588, 0.095)	0.120
GSK239512	50	-0.020			

	Treatment	*n*	Mean rate	Treatment comparison Ratio^c^ (90% CI)	
Cumulative number of new and enlarging lesions at Week 48					
New GdE lesions	Placebo	66	0.66	1.72 (1.05, 2.82)	
	GSK239512	64	1.14		
New and enlarging T2 lesions	Placebo	66	1.45	1.18 (0.75, 1.85)	
	GSK239512	64	1.71		
Cumulative unique active lesions	Placebo	66	1.48	1.17 (0.74, 1.85)	
	GSK239512	64	1.74		
New unenhanced T1 lesions	Placebo	66	0.50	1.38 (0.87, 2.18)	
	GSK239512	64	0.69		
Cumulative new GdE lesions evolving into chronic unenhancing T1 black holes	Placebo	63	0.29	1.71 (1.05, 2.78)	
	GSK239512	57	0.50		

Data taken from the AES, all evaluable scans dataset

*CI* confidence interval, *ES* effect size, *GdE* Gadolinium-enhanced, *ITT* intent-to-treat, *MRI* magnetic resonance imaging

^a^Adjusted mean change for normalised MTR is the post-lesion MTR value change from average pre-lesion MTR value

^b^ES is defined as the treatment difference divided by the standard deviation of the treatment difference averaged across relative MRIs

^c^Treatment Ratio (GSK239512/Placebo). A ratio <1 indicates a lower risk with GSK239512 versus placebo

A total of 50 relapses (24 with GSK239512 and 26 with placebo were reported in 38 patients [18 with GSK239512 and 20 with placebo]) during the treatment phase. No difference in relapse rates between treatment groups was observed (GSK239512: 0.417 and placebo: 0.400 relapses per 48 weeks). No meaningful changes from baseline were observed on EDSS and CogState battery throughout the treatment phase and at Week 48 (Table 4). Changes from baseline in MSQoL at Week 48 are shown in Online Resource 5.

**Table 4:** Summary of clinical outcomes (ITT population)

	*n*	Relapse rates: negative binomial regression		
		Mean rate of relapse (Total no. relapses)	Rate ratio (90% CI)	
Placebo	66	0.40 (26)	1.04 (0.62, 1.77)	
GSK239512	65	0.42 (24)		

	*n*	CogState score change from baseline		
		Adjusted mean (SE)	Difference versus placebo (90% CI)^a,b^	Effect size (90% CI)
Total score				
Placebo	56	0.16 (0.048)	−0.08 (−0.20, 0.04)	−0.24 (−0.59, 0.11)
GSK239512	48	0.08 (0.052)		
Executive function				
Placebo	55	0.08 (0.065)	−0.02 (−0.18, 0.14)	−0.05 (−0.37, 0.28)
GSK239512	47	0.06 (0.070)		
Memory				
Placebo	55	0.31 (0.070)	−0.15 (−0.32, 0.01)	−0.31 (−0.65, 0.03)
GSK239512	48	0.16 (0.075)		
Attention				
Placebo	54	−0.00 (0.088)	−0.06 (−0.27, 0.16)	−0.09 (−0.45, 0.26)
GSK239512	47	−0.06 (0.094)		

	*n*	EDSS score at Week 48^c^		
		Improved, *n* (%)	Unchanged, *n* (%)	Worsened, *n* (%)
Placebo	57	5 (9)	48 (84)	4 (7)
GSK239512	48	3 (6)	44 (92)	1 (2)

*CI* confidence interval, *EDSS* Expanded Disability Status Scale, *ITT* intent-to-treat, *MS* multiple sclerosis, *SE* standard error

^a^Difference in adjusted least squares means are shown (GSK239512 minus placebo). A positive treatment difference indicates benefit, relative to placebo

^b^The analysis method was mixed model repeated measures adjusted for treatment, visit, baseline total score, background MS disease-modifying treatment, treatment by visit, baseline total score by visit and background disease-modifying treatment by visit

^c^Improved defined as 1.0 decrease, worsened is defined as a 1.0 increase, in EDSS score; a positive treatment difference indicates benefit, relative to placebo

Overall, during the treatment phase the incidence of AEs was similar between GSK239512 (74%) and placebo (76%) and AEs were generally of mild and moderate intensity (28 and 42% for GSK239512 and 29 and 42% for placebo, respectively). However, during the titration period the incidence of AEs was higher with GSK239512 (52%) than with placebo (35%), particularly the incidence of insomnia (31 versus 9%, respectively), whereas in the maintenance period the overall incidence of AEs was similar (66 and 67%, respectively). The incidence of AEs judged by investigators as treatment-related was higher with GSK239512 versus placebo during the titration period (35 and 9%, respectively) than the maintenance period (11 and 3%, respectively). As expected, the most commonly reported AE during the treatment phase was insomnia, reported by 22 (34%) and seven (11%) patients in the GSK239512 and placebo groups, respectively; the 95% CI for the relative risk [3.19 (95% CI 1.57, 6.95)] excluded 1, suggesting a real difference. Other incidences of common AEs are shown in Table 5. Three SAEs were reported in two patients [oral papilloma and thrombophlebitis of the leg (*n* = 1) and thymoma (*n* = 1)] in the GSK239512 group and one SAE [nephrolithiasis (*n* = 1)] in the placebo group, during the maintenance period, with no SAEs reported during the titration period. In total, seven patients withdrew from the study due to AEs during the treatment phase, all in the GSK239512 group [insomnia (*n* = 2), thrombophlebitis (*n* = 1), cough (*n* = 1)], elevated alanine aminotransferase (ALT) and aspartate aminotransferase (AST; *n* = 1), thymoma (*n* = 1) and, axillary candidiasis, dry mouth, toxic skin eruption and xerosis (*n* = 1)]. No deaths were reported. Mean haematology, chemistry, urinalysis and vital sign values remained within normal ranges throughout the duration of the study, with the exception of one patient in the GSK239512 group, on a background of intramuscular interferon-β1a treatment, who had moderate level ALT and AST elevations. These AEs were judged by investigators as not treatment-related and this patient was withdrawn from the study.

**Table 5:** Summary of AEs (ITT population)

	Number (%) of patients reporting event					
	Titration period		Maintenance period		Treatment phase	
	Placebo, *N* = 66	GSK239512, *N* = 65	Placebo, *N* = 66	GSK239512, *N* = 65	Placebo, *N* = 66	GSK239512, *N* = 65
Any common event	23 (35)	34 (52)	44 (67)	43 (66)	50 (76)	48 (74)
AEs rated by intensity						
Mild	17 (26)	20 (31)	17 (26)	19 (29)	19 (29)	18 (28)
Moderate	6 (9)	14 (22)	24 (36)	21 (32)	28 (42)	27 (42)
Severe	0	0	3 (5)	3 (5)	3 (5)	3 (5)
Treatment-related AEs	6 (9)	23 (35)	2 (3)	7 (11)	8 (12)	27 (42)
SAEs	0	0	1 (2)	2 (3)	1 (2)	2 (3)
AEs Leading to withdrawal	0	1 (2)^a^	0	6 (9)	0	7 (11)
Common AEs ≥ 5% patients						
Insomnia	6 (9)	20 (31)	1 (2)	4 (6)	7 (11)	22 (34)
Middle insomnia	0	2 (3)	1 (2)	3 (5)	1 (2)	5 (8)
Nightmare	0	4 (6)	0	2 (3)	0	5 (8)
Headache	6 (9)	8 (12)	7 (11)	10 (15)	11 (17)	16 (25)
Dizziness	2 (3)	2 (3)	1 (2)	4 (6)	3 (5)	6 (9)
Fatigue	0	1 (2)	4 (6)	0	4 (6)	1 (2)
Pyrexia	1 (2)	0	3 (5)	1 (2)	4 (6)	1 (2)
Nausea	4 (6)	1 (2)	3 (5)	2 (3)	6 (9)	2 (3)
Diarrhoea	0	3 (5)	3 (5)	0	3 (5)	3 (5)
Nasopharyngitis	3 (5)	1 (2)	12 (18)	10 (15)	14 (21)	11 (17)
Vertigo	0	3 (5)	1 (2)	1 (2)	1 (2)	3 (5)
Palpitations	0	3 (5)	0	0	0	3 (5)
Influenza	0	0	4 (6)	5 (8)	4 (6)	5 (8)
UTI	1 (2)	2 (3)	1 (2)	4 (6)	2 (3)	5 (8)
Bronchitis	0	0	3 (5)	0	3 (5)	0
Cystitis	0	0	0	3 (5)	0	3 (5)
Neck pain	1 (2)	1 (2)	4 (6)	2 (3)	4 (6)	3 (5)
Arthralgia	0	0	4 (6)	0	4 (6)	0

ITT population is the same population as the safety population

*AE* adverse event, *ITT* intent-to-treat, *SAE* serious AE (defined as any AE or adverse reaction that results in death, is life-threatening, requires hospitalisation or prolongation of existing hospitalisation, results in persistent or significant disability or incapacity, or is a congenital anomaly or birth defect), *UTI* urinary tract infection

^a^AE leading to withdrawal started during the titration period but the patient did not withdraw until the maintenance phase

Minimum trough concentrations of GSK239512 were approximately 10–25 pg/mL for the 80 µg dose and were consistent over the study duration. No relationship between trough concentrations of GSK239512 and the primary endpoints were observed.

## Discussion

In this phase II, randomised, parallel-group, placebo-controlled study, GSK239512 at a maximum dose of 80 µg per day up-titrated over a 4- to 5-week period and maintained for 43–44 weeks had a positive effect on change in MTR, a marker of lesion remyelination in patients with RRMS.

Patient demographics and characteristics were as expected for patients with RRMS who met the defined eligibility criteria. While patient relapse data at screening may be suggestive of a more active population in the GSK239512 treatment group, the current analysis was based on lesion activity during the time period of the study. The on-study disease activity was broadly comparable between treatment groups with a similar mean relapse rate, proportion of patients with relapse and primary endpoint lesion activity. Therefore, the difference in baseline relapse activity did not appear to have any implications for the outcomes of this trial.

The primary MTR analysis focused on the comparison between pre-lesion and recovery values at times when these variables were relatively stable. GdE and Delta-MTR lesions that formed following the baseline MRI were identified using independent procedures, based on images with different contrast mechanisms. While GdE lesions are short-lived and sensitive to blood–brain barrier breakdown [31, 32], Delta-MTR lesions are persistent and appear to better identify tissue that has demyelinated [24]. Although their occurrence does not always co-localize, MTR recovery in either lesion type was expected to show the same response to treatment. This is indeed what we observed, with a small positive effect of GSK239512 versus placebo on lesion MTR being observed with effect sizes of 0.344 in GdE and 0.243 in Delta-MTR lesions with GSK239512 over placebo indicating a relative increase in remyelination, with high posterior probabilities of the effect size being >0 (0.955 in GdE and 0.877 in Delta-MTR lesions, respectively). Therefore, the MTR methodology employed in this study for assessing remyelination represents a promising tool for detecting changes in lesion myelin content in RRMS. However, in our study, the effect size was smaller than the target of 0.5. Additionally, the effect size in this trial was greater for Gd lesions compared with newly detected MTR lesions (Delta-MTR lesions) and may be associated with the greater number of GdE lesions included in the analysis due to relatively high-frequency (approximately monthly) scanning [24]. The use of MTR as a marker of remyelination has been previously validated, demonstrating that MTR values decrease with demyelination and increase with remyelination [10–17]. Studies on post-mortem brains from patients with MS have shown a strong association between MTR and histopathologically measured myelin content. The MTR of remyelinated lesions differs from both normal-appearing WM and demyelinated lesions [14, 16], and there is a significant correlation between myelin content and MTR in both lesions and the normal-appearing WM [14]. The methodology used to quantify changes in MTR over time associated with lesion remyelination has been previously reported [24].

Secondary MRI assessments did not demonstrate a positive impact of GSK239512 treatment that corresponded with the observed improvement in remyelination demonstrated by the MTR assessment. While unexpected from the pharmacologic action of the study drug, a small relative difference in favour of placebo was found. The magnitude of effect is unlikely to be clinically relevant, as analysis of pooled data from clinical trials has shown that an increase in relapse frequency is predicted only by larger differences in active lesion detection [33]. The absence of changes in secondary assessments, particularly MTR in chronic T2-weighted lesions may indicate the need for further development of additional remyelination marker endpoints, capable of demonstrating changes over time associated with improvement in remyelination [34, 35].

As a H_3_ receptor antagonist/inverse agonist, GSK239512 was not expected to improve or enhance the inflammatory disease process. Importantly, there was no indication that GSK239512 had an exacerbating effect, with similar relapse rates and EDSS scores observed in both treatment groups. No meaningful changes in brain volumes were observed; this was not unexpected given the relatively small changes in remyelination of lesions and extended time period required to observe changes in brain volume measurements in a sample of this size. Additionally, the patient population assessed had modest physical and cognitive impairment at baseline. While memory scores on the CogState battery improved slightly in both treatment groups this may be an artefact of learning or exercise effect.

Safety findings were consistent with previous studies of GSK239512 in AD and schizophrenia [6–8]. GSK239512 was well tolerated by the majority of patients, with 85% of patients reaching the 80 µg dose. Patients treated with GSK239512 reported more CNS-related AEs such as insomnia and nightmare than with placebo and sleep-related AEs were found to be the most common side effect of GSK239512; this is unsurprising for a RRMS population and the treatment mechanism of action, and is consistent with previous trials of GSK239512 in patients with AD [6, 7]. In total, seven patients withdrew due to AEs, all in the GSK239512 group and AE tolerability may have been a contributing factor to the differential withdrawal rate between the placebo and GSK239512 groups. Several of these were judged to be potentially treatment-related including liver function test elevation and toxic skin eruption and may be due to an interaction between GSK239512 and concomitant interferon-1βa therapy and should therefore be considered in future study designs. Trough plasma concentrations of GSK239512 at the 80 µg dose are consistent with >90% H_3_ receptor occupancy in the brain, as predicted from a previous positron emission topography study conducted in healthy human subjects [9].

One limitation of this trial was that not all patients contributed at least one lesion to the analysis as expected, making the results harder to generalise. Further studies could assess the effect of potential remyelinating agents in a wider patient population, including patients without lesions to further extend generalisability. Additionally, there was no clinical correlate of improved remyelination. Clinical outcome measures used generally require much larger sample sizes or longer study durations (2–3 years) to demonstrate clinically relevant changes. Future studies should consider evaluating the use of shorter-term clinical endpoints, include more patients and define a patient population with the capability of demonstrating a change. Additionally, targeted clinical deficits associated with specific demyelinating lesions might be more sensitive and could be reflected in additional surrogate endpoints [34, 35].

In summary, GSK239512 treatment resulted in a positive effect on lesion remyelination, detected by GdE and Delta-MTR lesion assessments, validating this approach for multicentre clinical trials. GSK239512 was found to have an acceptable safety profile in patients with RRMS. However, the positive effect on lesion remyelination did not translate into observed benefits on conventional MRI or clinical assessments. The development of clinical endpoints more directly linked to changes in myelination, inclusion of a population with more disease activity and/or a longer duration to match the clinical assessments used in the current trial are required to further explore the potential impact of an H_3_ receptor antagonist/inverse agonist on remyelination and ultimately disease progression in RRMS.

## Electronic supplementary material

Below is the link to the electronic supplementary material.

Supplementary material 1 (DOCX 140 kb)

Supplementary material 2 (PDF 1060 kb)
