# Supplementary material for: Lesion remyelinating activity of GSK239512 versus placebo in patients with relapsing-remitting multiple sclerosis: a randomised, single-blind, phase II study
Source: J Neurol. 2016 Nov 25;264(2):304–15. doi: 10.1007/s00415-016-8341-7 (PMC5306088; doi:10.1007/s00415-016-8341-7)
Supplement: Supplementary file 1 — Supplementary material 1 (DOCX 140 kb) [file 415_2016_8341_MOESM1_ESM.docx]

Lesion remyelinating activity of GSK239512 versus placebo in patients with relapsing-remitting multiple sclerosis: a randomised, single-blind, Phase II study

Caryl J Schwartzbach PhD,^1^ Richard A Grove MSc,^2^ Robert Brown, PhD,^3^ Debra Tompson MSc,^4^ Florian Then Bergh MD,^5,*^ Douglas L Arnold MD^3, 5,*^

^1^GSK, Research Triangle Park, Raleigh-Durham, NC, USA; ^2^GSK, Stockley Park, UK; ^3^McGill University, Montreal, QC, Canada; ^4^GSK, Gunnels Wood Road, Stevenage, Hertfordshire, UK; ^5^University of Leipzig, Germany; ^5^NeuroRx Research, Department of Neurology, Montreal, QC, Canada

*Joint last authors

**Corresponding author:** Caryl J Schwartzbach

**Email:** Schwartzbach@mindspring.com

# Online Resource 1: Methods

## List of primary investigators

**Bulgaria:** Lyubomir Haralanov, Ivan G Milanov, Milena Milanova, Ivailo Tournev, **Canada:** Amit
Bar-Or, Gregg Blevins, Mark S Freedman, Francois Jacques, Michael M Yeung; **Czech Republic:** Radek Ampapa, Jan Mares, Marta Vachova; **Germany:** Katrin Bachus-Banaschak, Veit U Becker, Peter Jörg Emrich, Anselm Kornhuber, Wilfried Lueer, Nelles Gereon, Ali Safavi, Karl-Otto Sigel, Florian Then Bergh, Heinz Wiendl, **Spain:** Jose Carlos Alvarez Cermeño, Rafael Arroyo González, Oscar Fernández, Juan Antonio García Merino, Guillermo Izquierdo Ayuso, Xavier Montalbán, Albert Saiz Hinarejos, Antonio Tallón Barranco, **Sweden:** Homayoun Roshanisefat; **Ukraine:** Anatoliy L Galusha, Viktoriia Gryb, Yevgeniya Lekomtseva, Nataliya V Lytvynenko, Sergiy P Moskovko, Tetyana Y Nehrych, Olga Shulga, Olena A Statinova, **UK:** Jeremy Chataway, Abhjit Chaudhuri, Eli Silber.

## List of internal Safety Review Committee members

Steve Hobbiger, Trevor Gibbs, Debbie Hepworth, Ethan Weiner, David H Miller and Ed Pattishall (replaced Dr Weiner from 4Q 2103).

## List of ethics committees

**Bulgaria:** Ethics Committee For Multicenter Clinical Trials, Sofia; **Canada:** MNI/MNH Research Ethics Board, Montreal, Quebec; Health Research Ethics Board, University of Alberta, Edmonton, Alberta; Ottawa Hospital Research Ethics Board, Ottawa, Ontario; Institutional Review Board Services, Ottawa, Ontario; Conjoint Health Research Ethics Board, Calgary, Alberta; **Czech Republic:** Nemocnice Jihlava, Vrchlickeho, Jihlava; Fakultni Nemocnice A Lf Up Olomouc, Pavlova, Olomouc; Krajska Zdravotni, A.S. – Nemocnice, Duchcovska, Teplice; **Germany:** Ethik-Kommission An Der Medizinischen Fakultaet der Universitaet Leipzig, Leipzig; **Spain:** Hospital Clínico San Carlos, S/Nºciudad Universitaria,Madrid; **Sweden:** Regionala Etikprövningsnämnden I Stockholm, Stockholm; **Ukraine:** Kyiv Regional Clinical Hospital, Thoracic And Pulmonological Center, Kyiv; Ivano-Frankivsk Regional Clinical Hospital, Ivano-Frankivsk; Institute Of Neurology, Psychiatry And Narcology, Kharkiv; Poltava Regional Clinical Hospital, Poltava; Vinnitsa Regional Psycho-Neurological Hospital, Vinnitsa; Lviv Regional Clinical Hospital, Lviv; Volyn Regional Clinical Hospital, Lutsk; Donetsk Regional Territorial Health Maintenance Association, Donetsk; **UK:** Health Research Authority, Bristol.

## Inclusion criteria

**Age**: 18–50 years of age, inclusive.

**Relapsing-remitting multiple sclerosis (RRMS) diagnosis and treatment**: Patients had to have a diagnosis of RRMS as defined by the appropriate McDonald criteria at the time of diagnosis [1]; a diagnosis made within approximately 10 years prior to the screening visit, and no physical manifestations of other forms of MS including signs of progression to secondary progressive MS; be compliant with a stable dose regimen of interferon-β1a or glatiramer acetate for management of MS for ≥1 year prior to the screening visit; have at least one reported and/or documented relapse, OR one Gadolinium-enhanced (GdE) lesion on magnetic resonance imaging (MRI) within the year preceding the screening visit AND after ≥2 months of stable treatment with interferon-β1a or glatiramer acetate (e.g. if treatment was initiated at 12 months prior to screening the earliest a lesion or relapse could occur to qualify for the study would be 10 months prior to screening and be neurologically stable at screening); and must not be actively experiencing or recovering from a recent relapse at the screening visit.

**MS disability**: A Kurtzke Expanded Disability Status Scale [2] score of 1–4.5 (inclusive) at the screening visit was required.

**Clinical trial commitment:** Patients agreed not to participate in a clinical study involving another investigational drug or device throughout their participation in this study. Non-interventional study participation was allowed if the time involvement and scheduling would not interfere with compliance in this study in the opinion of the investigator.

**Female reproduction and lactation**: A female patient was eligible to enter the study if she was:

not pregnant or nursing; of non-childbearing potential; or, if women were of childbearing potential they must provide a negative serum pregnancy test at screening and agree to either complete abstinence from intercourse or consistent and correct use of an acceptable method of birth control for 1 month prior to the start of investigational product to 1 month after the last dose of investigational product.

**Informed consent:** Patients must be competent to understand the information given in the Institutional Review Board or Independent Ethics Committee approved informed consent form and must sign the form prior to the initiation of any study procedures.

## Exclusion criteria

**MRI:** Patients unable to undergo MRI scans (e.g. due to pacemaker, severe claustrophobia, hypersensitivity to contrast media); patients who lacked adequate venous access for administration of GdE agent; or findings on brain MRI scan that indicated any clinically significant brain abnormality other than MS (e.g. damage associated with prior traumatic brain injury) were excluded.

**Past and concurrent medical conditions**: History of severe and clinically significant central nervous system trauma with current sequelae (e.g. traumatic brain injury, spinal cord compression); significant concurrent, uncontrolled medical condition (e.g. significant psychiatric disorder) which could affect the patients’ safety, impair their reliable participation in the trial, impair the evaluation of the endpoints, or necessitate the use of medication not allowed by this protocol; history/presence of myelopathy due to spinal cord compression by disk or vertebral disease or chronic progressive myelopathy; a diagnosis of any type epilepsy; considered at risk of suicide; presence of significant and routine sleep disturbance (e.g. severe insomnia, nocturnal wandering, confusion, disorientation, agitation, or vivid dreams) that might have increased the risk of tolerability issues during dose escalation; presence/history of hallucinations that may increase the safety risk to the patient; or a known diagnosis/history consistent with positive human immunodeficiency virus.

**Cardiovascular status at screening:** Patients were excluded if their electrocardiogram showed a clinically significant abnormality at screening including a QTcB or QTcF interval of ≥450 msec or ≥480 msec for patients with a Bundle Branch Block.

**Infectious disease status at screening:** Patients with serologic evidence of active or chronic Hepatitis B including those patients testing positive for Hepatitis B surface antigen (HBsAg) and total Hepatitis B core antibody (anti-HBc, immunoglobulin G) were excluded. Patients with serologic evidence of active Hepatitis C, as indicated by a positive HCV RNA test and anti-HCV antibody test were also excluded.

**Laboratory values at screening:** Patients with haematology values of: total white cell count <2.0 x 10^9^/L; neutrophils <1.0 x 10^9^/L; platelets <75 x 10^9^/L (if out of range, platelet count can be repeated to exclude platelet clumping); haemaglobin <80 g/L were excluded. Clinical chemistry liver function test values of: alanine aminotransferase >2.0 x upper limit of normal (ULN); aspartate aminotransferase >2.0 x ULN; alkaline phosphatase >1.5 x ULN; bilirubin >1.5 x ULN were also excluded. Other criteria were documented renal insufficiency or laboratory results indicative of renal insufficiency (due to risks associated with administration of Gd-based contrast agents to patients with moderate to severe kidney disease): estimated creatinine clearance (Cockroft-Gault)
<60 mL/minute.

**Protocol compliance:** If it was known that, or according to investigator judgement, patient was suspected of not being able to comply with the study protocol requirements, they were excluded. Contributing factors to this assessment by the investigator could be, but not limited to: job demands, substance abuse, alcoholism, drug dependency or psychological disorder.

**Prior clinical trial experimental therapy experience**: Prior participation in a clinical trial or use of an investigational product for a non-approved intervention; prior use of an investigational drug for a condition other than MS within 4 weeks or 5 half-lives (whichever is longer) prior to screening; prior use of an investigational drug for MS within 4 weeks or 5 half-lives (whichever is longer) prior to screening.

## GdE and Delta-MTR lesion identification

GdE lesions had to meet the following criteria: (1) the lesion had to have at least 3 contiguous voxels that enhanced by at least 20% on the post-gadolinium T1-weighted image relative to the pre-gadolinium T1-weighted image; (2) the enhancing voxels on the post-gadolinium T1-weighted image had to partially co-localize with, or be adjacent to, a T2-weighted lesion. Delta-MTR lesions had to meet the following criteria: each lesion voxel had to (1) exhibit a decrease in MTR relative to the previous scan that was greater than the 99^th^ percentile of the distribution of decreases observed in high confidence (>80% probability) normal appearing WM; (2) have a final MTR value that was greater than the 5^th^ percentile of the distribution of MTR values observed in high confidence normal-appearing GM; (3) be identified as belonging to a T2 hyperintense lesion on the present scan, and within normal-appearing WM on the previous scan; and (4) be within the brain mask on both the present and previous scan. Individual lesions were identified and labelled as groups of lesion voxels under a six-connectedness criterion in three dimensions. Identified lesions were reviewed by trained MRI readers. New GdE and Delta-MTR lesions were defined as lesions that were not present on the previous scan. Two qualified MRI readers identified lesions and then met to perform a Consensus read.

## MRI protocol

NeuroRx Research provided centralised MRI analysis for MRI methodology. MRI scans were performed prior to randomisation and at Week 6, 12, 18, 24, 30, 36, 42, and 48 (±14 days) using a standardised protocol for all sites consisting of the following sequences: fast localiser scan (3 PLANE), true mid-line sagittal scan: T1 weighted, TR: 350 ms, TE: 18 ms, 3 mm slices; fast axial scan for repositioning, Proton Density-weighted: 60 slices; T2-weighted: 60 slices; MT OFF sequence: 60 slices, MT ON sequence: 60 slices, T1-weighted pre-gad: 60 slices, Turbo FLAIR sequence and T1-weighted post-gad sequence. Small amendments were allowed to accommodate for contrast with different field strengths and scanners. Dummy scans were performed at each site on a healthy volunteer using standardised scanning procedures without Gd contrast, to ensure image quality control and provide scanner-specific magnetisation transfer ratio (MTR) data for normalisation.

**Sample size for final analysis**

A sample size of approximately 100 patients was considered sufficient to assess a positive signal if (PP[Δ>0]>80%) for both co-primary endpoints or observed if one co-primary endpoint has a PP(Δ>0)>80% and the other co-primary endpoint has a PP(Δ>0)>70%; negative signal if both co-primary endpoints have a PP(Δ>0)<70%; unclear signal otherwise. No formal Type I or II error rates were defined and the sample size was based on 10,000 simulations. Accounting for dropouts, 114 patients were planned to be randomised.

## Sample size for interim analysis

A sample size of approximately 50 patients was considered sufficient to investigate the chance of stopping for futility and correctly detecting a signal. The simulations were based on the following assumptions: a target effect size of 0.5; correlation of the co-primary endpoints of 0.5; all patients would contribute at least one lesion to each endpoint; interim analysis conducted when 50% patients reached Week 42 (futility criteria: both co-primary endpoints have a posterior probability (PP; [Δ>0]<30%).

**Statistical analysis**

Analysis of the co-primary endpoints used a mixed model for repeated measures (MMRM). The date of identification of a lesion is labelled as the reference MRI, and all visits are assessed relative to that visit, described as relative MRIs. Post-lesion MTR value change from average pre-lesion MTR value for each lesion was modelled separately across relative MRI within each patient allowing for variation in effects between patients, between different lesions within patients, and within lesions across time. Variability between patients and lesions was modelled using an unstructured covariance matrix and measurements over time within the same lesion followed an auto-regressive structure. At the patient level we introduced a random patient effect.

Fixed effects in the model were treatment, average pre-lesion MTR value, relative MRI, lesion size, background disease-modifying treatment (DMT); and interactions between relative MRI and treatment, between average pre-lesion MTR value and relative MRI, between background DMT and relative MRI and between lesion size and relative MRI.

The models were used to estimate treatment effects of GSK239512 relative to placebo and were also averaged across all post-lesion MRIs. Standardised effect sizes, derived as the treatment difference divided by the standard deviation (estimated as the between-patient variability from the analysis model) of the treatment difference averaged across visits/relative MRIs were calculated.

Bayesian analysis was based on a non-informative prior, using the prior statement within the Markov chain Monte Carlo simulation procedure, and using the inverse Wishart prior option in SAS v9.3 to estimate the posterior probabilities the standardised effect size for the change in MTR value post-lesion relative to pre-lesion being greater than 0 from 500,000 simulations.

Lesion count endpoints and relapses were analysed using a generalised linear model with underlying negative binomial distribution. Other secondary endpoints measured repeatedly during the treatment period (T2 lesion MTR, white matter/grey matter volumes, CogState battery) were analysed using an MMRM approach using restricted maximum likelihood estimation with an unstructured covariance matrix. Secondary endpoints collected pre-treatment and at Week 48 only (brain atrophy, Multiple Sclerosis Quality of Life-54) were analysed using analysis of covariance adjusting for baseline measure and background DMT. Adverse events were coded using the Medical Dictionary for Regulatory Activities and summarised by system organ class and preferred term. Pharmacokinetic data were summarised descriptively by nominal sampling times at Week 8, by Week (4, 8, 24, 36, and 48) and dose level.

A Mixed-Model Repeated Measures statistical approach was implemented, which for data missing at random (i.e. when there is no explainable reason for the data to be missing in the data eg scan was missed or unreadable) looks at the covariance matrix and estimates values for missing data based on similar covariate patterns within the data

## Interim analysis

The Interim analysis of lesions available from the 46 patients who reached their Week 42 MRI visit, indicated an effect size of -0.118, in favour of placebo for the change in GdE lesion MTR and 0.335, in favour of GSK239512 and Delta-MTR lesion MTR, respectively. The study did not the meet the futility criteria specified in the statistical analysis plan (both co-primary endpoints (GdE: 0.396 and dMTR: 0.812) had a PP[∆>0] <30% at the time of the interim analysis) and so carried on to its specified conclusion.

**Online Resource 2.**

Assuming a non-informative prior distribution, 95.5% of the distribution for GdE lesions (Online Figure 1) and 87.7% for Delta-MTR lesions (Online Figure 2) suggested an Effect Size >0. i.e. the likely true treatment difference is going to be greater than equality (variability estimation permitting).

**Online Figure 1. Posterior probability of an MTR value post-lesion vs pre-lesion for GdE lesion effect size with GSK239512 versus placebo**


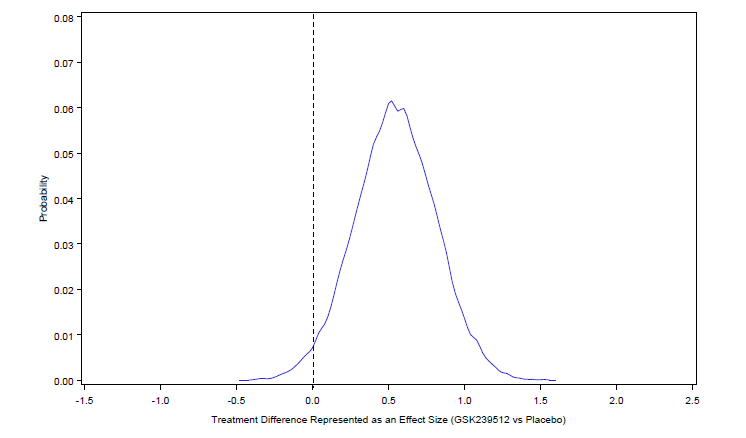


MTR, magnetic transfer ratio.

**Online Figure 2. Posterior probability of an MTR value post-lesion vs pre-lesion for Delta-MTR lesion effect size with GSK239512 versus placebo**


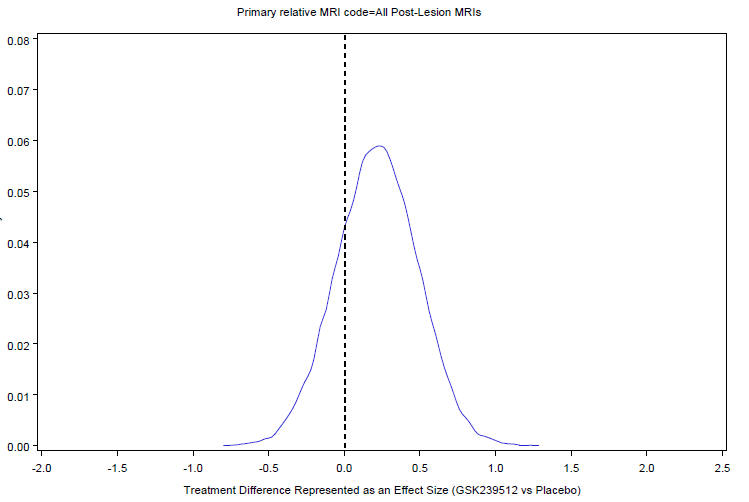


MTR, magnetic transfer ratio.

**Online Resource 3: Post hoc analysis of new lesion formation at Week 48 by lesion status at screen MRI (ITT population)**

| **Cumulative number of lesions at Week 48** | | | | |
| --- | --- | --- | --- | --- |
|  | | | | Treatment comparison |
| **New GdE lesions** | Treatment | n | Mean rate | ratio^a^ (90% CI) |
| **Patients with no GdE lesions on screening MRI** | Placebo | 42 | 0.24 | 2.36 (1.18, 4.70) |
|  | GSK239512 | 47 | 0.57 |  |
| **Patients with at least ONE GdE lesion on screening MRI** | Placebo | 24 | 1.77 | 0.78 (0.44, 1.40) |
|  | GSK239512 | 17 | 1.38 |  |

CI, confidence interval; GdE, gadolinium-enhanced lesion; ITT, intent-to-treat; MRI, magnetic resonance imaging; data taken from the all evaluable scans dataset.

^a^A ratio <1 indicates a lower risk with GSK239512 compared with placebo.

**Online Resource 4. Summary brain volume data (ITT population)**

Whole brain volumes were within the expected range of healthy patients [3,4] and similar between treatment groups.

|  | **Screening MRI (cm^3^)**  **mean (SD)** | | **Statistical analysis of change from screening (cm^3^)** | | |
| --- | --- | --- | --- | --- | --- |
|  |  |  | **Adjusted mean (SE)** | |  |
|  | **Placebo**  **(N=66)** | **GSK239512**  **(N=65)** | **Placebo**  **(N=66)** | **GSK239512**  **(N=65)** | **Difference vs placebo**  **(90% CI)** |
| **n** | 65 | 65 | 59 | 50 |  |
| **Whole brain volume** | 1486.3 (79.78) | 1475.4 (68.87) | -3.6 (0.81) | -4.6 (0.89) | -1.1  (-3.1, 0.9) |
| **WM volume** | 779.4 (41.33) | 776.7 (36.26) | 1.2 (0.55) | 0.2 (0.59) | -1.1  (-2.4, 0.3) |
| **GM volume** | 561.7 (51.50) | 555.3 (49.06) | -4.1 (0.52) | -4.4 (0.55) | -0.3  (-1.6, 1.0) |

CI, confidence interval; GM, grey matter; MRI, magnetic resonance imaging; SD, standard deviation; SE, standard error; WM, white matter.

**Online Resource 5. Summary of clinical outcomes (ITT population)**

| **MSQoL-54 [5] adjusted mean composite score changes from baseline at Week 48** | | | |
| --- | --- | --- | --- |
|  | **n** | **SE of adjusted mean^a^** | **Difference vs placebo (90% CI)^b^** |
| **Physical health composite score** | | | |
| **Placebo** | 65 | 1.98 | -2.95 (-6.72, 0.82) |
| **GSK239512** | 63 | -0.97 |  |
| **Mental health composite score** | | | |
| **Placebo** | 65 | 2.13 | -5.02 (-9.06, -0.98) |
| **GSK239512** | 63 | -2.89 |  |

CI, confidence interval; GdE, gadolinium-enhanced lesion; ITT, intent-to-treat; MRI, magnetic resonance imaging; MSQoL, Multiple Sclerosis Quality of Life-54; SE, standard error.

^a^Difference in adjusted least squares means are shown (GSK239512 minus placebo); ^b^the analysis method was analysis of covariance adjusted for treatment, baseline physical health composite score and background MS disease-modifying treatment.

# References

1. Polman CH, Reingold SC, Banwell B, Clanet M, Cohen JA, Filippi M, Fujihara K, Havrdova E, Hutchinson M, Kappos L, Lublin FD, Montalban X, O'Connor P, Sandberg-Wollheim M, Thompson AJ, Waubant E, Weinshenker B, Wolinsky JS (2011) Diagnostic criteria for multiple sclerosis: 2010 revisions to the McDonald criteria. Annals of neurology 69(2): 292-302.
2. Kurtzke JF (1983) Rating neurologic impairment in multiple sclerosis: an expanded disability status scale (EDSS). Neurology 33(11): 1444-52.
3. Kruggel F (2006) MRI-based volumetry of head compartments: normative values of healthy adults. NeuroImage 30(1): 1-11.
4. De Stefano N, Iannucci G, Sormani MP, Guidi L, Bartolozzi ML, Comi G, Federico A, Filippi M (2002) MR correlates of cerebral atrophy in patients with multiple sclerosis. Journal of Neurology 249(8): 1072-7.
5. Vickrey BG, Hays RD, Harooni R, Myers LW, Ellison GW (1995) A health-related quality of life measure for multiple sclerosis. Quality of life research: an international journal of quality of life aspects of treatment, care and rehabilitation 4(3): 187-206.
