# Supplementary material for: Lesion remyelinating activity of GSK239512 versus placebo in patients with relapsing-remitting multiple sclerosis: a randomised, single-blind, phase II study
Source: J Neurol. 2016 Nov 25;264(2):304–15. doi: 10.1007/s00415-016-8341-7 (PMC5306088; doi:10.1007/s00415-016-8341-7)
Supplement: Supplementary file 2 — Supplementary material 2 (PDF 1060 kb) [file 415_2016_8341_MOESM2_ESM.pdf]

Division: World Wide Development

Retention Category: GRS019
Information Type: Reporting and Analysis Plan

**Title:**	Reporting and Analysis Plan for H3M116477: Proof of Mechanism Study to Assess the Potential of GSK239512 to Remyelinate Lesions in Subjects with Relapsing Remitting Multiple Sclerosis

Compound Number: GSK239512

Effective Date: 30-SEP-2014

Description: This document provides the reporting and analysis plan for this Proof of Mechanism Study to Assess the Potential of GSK239512 to Remyelinate Lesions in Subjects with Relapsing Remitting Multiple Sclerosis

Subject: Relapsing Remitting Multiple Sclerosis, Remyelination, H3 inverse agonist, magnetic Resonance Imaging, Magnetisation Transfer Ratio

Author's Name, Title and Functional Area: [redacted] Manager, Neurosciences Clinical Statistics; [redacted] Director, Clinical Pharmacology, Modelling and Simulation.

Approved via e-mail by:

[redacted] NS Clin Stats TA Head

[redacted] Director, Development Sciences, Neurosciences TAU

Copyright 2014 the GlaxoSmithKline group of companies. All rights reserved. Unauthorised copying or use of this information is prohibited.

TABLE OF CONTENTS

PAGE

ABBREVIATIONS 5

1. INTRODUCTION 7

2. STUDY OBJECTIVE(S) AND ENDPOINT(S) 7
   2.1. Study Objectives and Endpoints 7
   2.2. Statistical Hypotheses 9
   2.3. Pharmacokinetic (PK) and PK/Pharmacodynamic (PD) hypotheses 10

3. STUDY DESIGN 10

4. PLANNED ANALYSES 11
   4.1. Interim Analyses 11
   4.2. Final Analysis 12

5. SAMPLE SIZE CONSIDERATIONS 12
   5.1. Sample Size Assumptions 12
   5.2. Sample Size Sensitivity 14
   5.3. Sample Size Re-estimation 17

6. ANALYSIS POPULATIONS 17
   6.1. Analysis Datasets 17
      6.1.1. Non-MTR MRI Endpoints 17

7. TREATMENT COMPARISONS 18
   7.1. Data Display Treatment and Other Sub-group Descriptors 18

8. GENERAL CONSIDERATIONS FOR DATA ANALYSES 18
   8.1. Multicentre Studies 18
   8.2. Other Strata and Covariates 19
   8.3. Examination of Subgroups 20
   8.4. Multiple Comparisons and Multiplicity 20
   8.5. Genetic Markers 20

9. DATA HANDLING CONVENTIONS 20
   9.1. Premature Withdrawal and Missing Data 20
   9.2. Derived and Transformed Data 21
      9.2.1. Baseline Data 21
      9.2.2. Demographic Data 21
      9.2.3. Study Day Definition 21
      9.2.4. Defining the First Dose Date 21
      9.2.5. Defining the Last Dose Date 22
      9.2.6. Defining Titration and Maintenance Periods 22
      9.2.7. MRI – MTR Data 23
      9.2.8. MRI – Other MRI Endpoints 24
         9.2.8.1. Conventional MRI endpoints from Central Reader 24
         9.2.8.2. MRI endpoints to be Derived Programmatically 25

9.2.9. CogState Battery 27
9.2.10. MSQoL54 29
9.2.11. Electrocardiograms (ECGs) 31
9.3. Assessment Windows 31
9.3.1. Study Phases 31
9.3.2. Assessment Windows 32
9.4. Values of Potential Clinical Importance 34
9.4.1. Laboratory Data 34
9.4.2. Vital Signs 35
9.4.3. ECGs 35

10. STUDY POPULATION 35
10.1. Disposition of Subjects 36
10.2. Protocol Deviations 36
10.3. Demographic and Baseline Characteristics 37
10.3.1. Demographics 37
10.3.2. Medical History 38
10.3.3. MS Disease History 38
10.3.4. Smoking History 38
10.4. Concomitant Medications 39
10.5. Exposure and Treatment Compliance 42
10.5.1. Exposure 42
10.5.2. Treatment Compliance 43
10.5.3. Dose Titration 43

11. EFFICACY ANALYSES 44
11.1. Primary Efficacy Analyses 44
11.1.1. Summary Displays 44
11.1.2. Statistical Analysis 45
11.2. Secondary MRI Efficacy Analyses 48
11.2.1. T2 Lesion MTR 48
11.2.2. MRI Lesion Count Variables 49
11.2.2.1. Summary Displays 49
11.2.2.2. Statistical Analysis 50
11.2.3. MRI Volume Measures 52
11.2.3.1. Summary Displays 52
11.2.3.2. Statistical Analysis 52
11.3. Other Efficacy Analyses 53
11.3.1. CogState Battery 53
11.3.1.1. Summary Displays 53
11.3.1.2. Statistical Analysis 54
11.3.1.3. Sensitivity Analysis around Missing Data 56
11.3.2. Exploratory Analyses: Understanding Relationships 57

12. SAFETY ANALYSES 57
12.1. Adverse Events 57
12.1.1. General Rules for AEs 58
12.1.2. Counting Rules for AEs 59
12.1.3. AE Collapsing Algorithm 60
12.1.4. Adverse Event Reporting 60
12.1.5. Adverse Events of Special Interest 62
12.1.6. Possible Suicidality Related Adverse Events 63

12.2. Deaths and Serious Adverse Events 63
12.3. Device Incidents and Near Incidents 63
12.4. Adverse Events Leading to Discontinuation of Investigational Product and/or Withdrawal from the Study and Other Significant Adverse Events 64
12.5. Pregnancies 64
12.6. Clinical Laboratory Evaluations 64
12.6.1. Haematology and Chemistry 64
12.6.1.1. Liver Function Tests 65
12.6.2. Urinalysis Results 66
12.7. Multiple Sclerosis Safety Assessments 66
12.7.1. EDSS 66
12.7.2. MS Relapses 67
12.8. Other Safety Measures 68
12.8.1. Vital Signs 68
12.8.2. ECG Assessments 68
12.8.3. Columbia Suicide Severity Rating Scale (C-SSRS) 69

13. HEALTH OUTCOMES ANALYSES 70
13.1. MSQoL54 70
13.1.1. Summary Displays 70
13.1.2. Statistical Analysis 70

14. CLINICAL PHARMACOLOGY DATA ANALYSES 71
14.1. Pharmacokinetic Analyses 71
14.2. Pharmacokinetic/Pharmacodynamic Analyses 71

15. REFERENCES 72

16. ATTACHMENTS 73
16.1. Table of Contents for Data Display Specifications 73
16.2. Data Display Specifications 101
16.3. Laboratory Normal Ranges and Potential Clinical Concern Ranges 102

ABBREVIATIONS

AE	Adverse Event
AES	All Evaluable Scans
AESI	Adverse Event of Special Interest
ALP	Alkaline Phosphatase
ALT	Alanine Amino Transferase
ANCOVA	Analysis of Covariance
AST	Aspartate Amino Transferase
ATC	Anatomical Therapeutic Class *
BIL	Total Bilirubin
BMI	Body Mass Index
bpm	Beats per Minute
BPT	Biostatistics & Programming Team
CUAL	Cumulative Unique Active Lesions
CSR	Clinical Study Report
DM	Data Management
ECG	Electrocardiogram
eCRF	electronic Case Report Form
eC-SSRS	electronic Columbia-Suicide Severity Rating Scale
EDSS	Expanded Disability Severity Scale
FSH	*
FSS	Functional System Score
Gd	Gadolinium
GdE	Gadolinium-enhancing
GGT	Gamma-Glutanyl Transferase
GMLT	Groton Maze Learning Test
GSK	GlaxoSmithKline
HLGT	Higher Level Grpoup Term
IMV	Imputed Missing Values
ISLT	International Shopping List Task – Immediate Recall
ISLT-R	International Shopping List Task – Delayed Recall
iSRC	internal Safety Review Committee *
ITT	Intention-to-Treat
kg	Kilogram
m	metre
MedDRA	Medical Dictionary for Regulatory Affairs
mmHg	millimeters of mercury
MMRM	Mixed Model Repeated Measures
MS	Multiple Sclerosis
MSQoL	Multiple Sclerosis Quality of Life
MTR	Magnetisation Transfer Ratio
MRI	Magnetic Resonance Imaging
NR	Non-Randomised Population
OBT	One Back Test
OC	Observed Case
OCL	One Card Learning
PCC	Potential Clinical Concern

PCI	Potential Clinical Importance
PD	Pharmacodynamics
PK	Pharmacokinetics
PP*	Per Protocol
PSRAE	Possible Suicide-Related Adverse Event
PT	Preferred Term
QC	Quality Control
R	Randomised Population
R&D	Research & Development
RAP	Reporting and Analysis Plan
RRMS	Relapsing Remitting Multiple Sclerosis
RUCAM	Roussel Uclaf Causality Assessment Method
SAE	Serious Adverse Event
SD	Standard Deviation
SOC	System Organ Class
SPM	Study Procedures Manual
SOP	Standard Operating Procedure
SPMS	Secondary Progressive Multiple Sclerosis
SRT	Safety Review Team
ug	Microgram
ULN	Upper Limit of Normal

Trademark Information

Trademarks of the GlaxoSmithKline group of companies
NONE

Trademarks not owned by the GlaxoSmithKline group of companies
Avonex
Copaxone
SAS

1. INTRODUCTION

This document details the reporting and analysis to be prepared by the Biostatistics and Programming Team, Neuroscience for this Proof of Mechanism Study to Assess the Potential of GSK239512 to Remyelinate Lesions in Subjects with Relapsing Remitting Multiple Sclerosis. The RAP has been written and approved whilst the project team remain blinded to treatment assignments. As per the protocol, some project team members may be unblinded at the time of the iSRC conducting the interim analysis. This sub-team will be reviewing data summaries whilst the study is conducted and analyses not specified in this RAP may be planned for final reporting. These analyses will be identified as being planned after unblinding in the CSR.

This RAP is based on Guidance document GUI_00000137354 and is in line with SOP_00000054838

The RAP references the H3M116477 protocol [GlaxoSmithKline Document Number HM2012N133918_00] and the study eCRF.

This RAP is intended for use by GSK staff within R&D involved in the delivery of data displays for study H3M116477. Version 2 of this RAP was generated based on comments from the instream blinded data look and following review of the analysis of the novel co-primary endpoints after the futility interim analysis at a time when the author was unblinded at the subject level and the approvers were unblinded to the treatment group results. The updates have been made to provide a clear reporting strategy for the programmers on this study based on information learned on review of the interim analysis results for the imaging and clinical endpoints. The unblinded sub-team did not review any grouped safety data. When version 1 was approved, no blinded data had been seen for the co-primary endpoints so updates in Section 11 are based on the opportunity to learn about their distribution of those endpoints at the time of the interim analysis.

2. STUDY OBJECTIVE(S) AND ENDPOINT(S)

2.1. Study Objectives and Endpoints

The study objectives and endpoints are detailed in Table 1.

Table 1 Objectives and Endpoints

Primary	
**Objectives**	**Endpoints**
Estimation of the effects of GSK239512 on lesion remyelination in subjects with Relapsing Remitting Multiple Sclerosis on stable treatment with Avonex [interferon-beta1a] or Copaxone [glatiramer acetate].	Changes in lesion myelination using two exploratory magnetization transfer ratio (MTR) endpoints comparing placebo to GSK239512 treated subjects in:<br>1) Mean change in gadolinium (Gd) enhanced (GdE) lesion MTR differences (calibrated to reference scan [Section 6.3.1.1]) from before enhancement to

	stable recovery (≥3 months post new GdE lesion), and<br>2) Mean change in Delta MTR lesion MTR differences (calibrated to reference scan) from before lesion appearance to stable recovery (≥3 months post lesion appearance).
**Secondary**	
**Objectives**	**Endpoints**
Evaluate the effects of GSK239512 on alternate potential marker of remyelination of lesions.	Change from baseline in T2 lesion MTR at Week 48.
Evaluate the effects of GSK239512 on brain MRI lesion counts.	Cumulative new and enlarging Gd enhancing, T2 and Combined Unique Active lesions comparing placebo to GSK239512 treated subjects.
Evaluate the effects of GSK239512 on overall neurodegeneration by assessing brain volumes (Total, white matter and grey matter)	Change from baseline at Week 48 in total brain volume, white matter volume and grey matter volume comparing placebo to GSK239512 treated subjects.
Evaluate the effects of GSK239512 on T1 hypointense lesion development associated with MS	Cumulative number of persistent black holes and new, unenhancing T1 lesion counts comparing placebo to GSK239512 treated subjects over treatment duration up to Week 48.
	Proportion of new GdE lesions evolving into chronic (unenhancing) T1 lesions (black holes) comparing placebo to GSK239512 treated subjects over treatment duration up to Week 48.
Evaluate the effect of GSK239512 on Cognitive ability	Mean change from baseline in overall Cognitive impairment and cognitive domains comparing placebo to GSK239512 treated subjects.
Evaluate the effect of GSK239512 on relapses	Comparison of Relapse Rates between placebo and GSK239512 treated subjects.
	Comparison of Time to First Relapse between placebo and GSK239512 treated subjects.
	Comparison of the proportion of subjects Relapse Free between placebo and GSK239512 treated subjects
Evaluate the effect of GSK239512 on Disability and Functionality	Proportion of subjects with sustained worsening of Expanded Disability Severity Scale (EDSS) over 3 months comparing placebo to GSK239512 treated subjects.
	Mean change from baseline in the EDSS functional systems and a subset of component assessments comparing placebo to GSK239512 treated subjects.

Safety	
**Objectives**	**Endpoints**
Evaluate the safety and tolerability of GSK239512	Frequency and severity of AEs and SAEs.
	Percentage of subjects withdrawing due to AEs.
	Summary of suicide behavior and ideation risk as assessed by the eC-SSRS and PSRAE.
	Change from baseline in clinical chemistry, hematology, and urinalysis parameters.
	Frequency of clinical chemistry, hematology, and urinalysis parameters of potential clinical concern.
	Change from baseline in vital signs and ECG parameters.
	Frequency of vital signs and ECG parameters of potential clinical concern.
**Pharmacokinetic**	
**Objectives**	**Endpoints**
Evaluate pharmacokinetics of GSK239512 in MS subjects	Pre-dose trough concentration at Wk 4, Wk 24, Wk 36 and Wk 48 with sparse sampling (1 pre-dose and 3 post-dose) at Wk 8.
**Exploratory**	
**Objectives**	**Endpoints**
Evaluate the effect of GSK239512 on MS symptoms and quality of life	Mean change from baseline in the Multiple Sclerosis Quality of Life (MSQoL) 54 comparing GSK239512 and placebo treated subjects

2.2. Statistical Hypotheses

There are no statistical hypotheses to be tested in this study. The primary objectives of this study are around estimation of the effects of remyelination of GSK239512 versus placebo. These objectives will be assessed by the generation of predictive probabilities that the remyelination of new lesions is of an effect size greater than zero and considered of potential clinical relevance. The target effect size for this study is 0.5. To this extent, inference will be carried out to estimate the posterior probability that the difference between the mean of the test treatment and the mean of the reference treatment μ(GSK239512) - μ(Placebo), is greater than 0 for each co-primary endpoint using effect sizes. An effect size is defined as the difference of the means in the two treatment groups divided by the standard deviation from the statistical model. The posterior probability that the true effect size is greater than zero will be referred to as PP(Δ>0).

In addition in this study it is also of interest to estimate the changes in the co-primary endpoints that are of potential clinical relevance, define their statistical distributions, estimate their relationship and explore the most appropriate statistical methodology to analyses these endpoints.

2.3. Pharmacokinetic (PK) and PK/Pharmacodynamic (PD) hypotheses

There are no PK or PK/PD hypotheses to be tested in this study. Analyses will be descriptive and exploratory.

3. STUDY DESIGN

This study is randomized, parallel group, and placebo-controlled. Subjects with RRMS on stable background treatment with either Avonex (Interferon-beta1a) or Copaxone (Glatiramer Acetate) are eligible to participate.

Subjects will be randomized in a 1:1 ratio between placebo and GSK239512. Randomization will be stratified by Background Disease Modifying Therapy (DMT) of either Avonex or Copaxone.

The total treatment period is 48 weeks, including a standard 4 week titration period and 44 week maintenance treatment period (which could be adapted to a 5-week titration and 43 week maintenance period, if needed [Protocol Section 3.3]). Titration doses start at 10µg and increase up to 80µg (10µg first week, 20µg second week, 40µg third week, 80µg fourth week). Subjects will be titrated to the maximum tolerated dose with the objective of titrating to the highest dose (80µg GSK239512), whenever possible, based on investigator judgement of tolerability, as described in Protocol Section 5.3. Subjects unable to titrate to 80µg will be allowed to remain in the study, at the highest dose that they were able to tolerate. The post-treatment follow-up period will be a minimum of 2 weeks in duration following the end of treatment at Week 48 or early withdrawal, as appropriate.

Safety data including all safety assessments will be reviewed throughout the study by the Safety Review Team (SRT) for GSK239512. Trends in clinical endpoint data (e.g. relapse, EDSS, etc...) to assess signals of disease worsening that could be indicative of a safety signal, will be reviewed and may include, as appropriate, the engagement of external expert consultation. At a minimum, these reviews will include efficacy data approximately every 3 months following randomization of the first subject. An internal Safety Review Committee (iSRC) consisting of GSK personnel who are not involved in the conduct of this study will be assembled and chartered to answer specific questions on behalf of the study team (Protocol Section 6.4.9).

The study will be conducted as a 'single-blind' study. Only, a subset of individuals defined in the iSRC Charter will be unblinded. ALL subjects and non-GSK personnel (including the principal investigator, sub-investigators, and study site personnel) involved in measuring, monitoring and obtaining data in the study will be blinded to subject treatment assignment. In addition all GSK and vendor personnel involved in the on-site monitoring and direct management of the data and study sites will also remain blinded to individual subject data.

The study will be conducted as a 'single-blind' study due to the exploratory nature of the study and use of the endpoints. The instream reviews of the MRI data will be conducted at intervals throughout the study with available subject data as defined in Section 8 and

the iSRC Charter, including involvement of external MS and/or imaging expert(s), as appropriate. The SRT will review study data as part of the standard SRT monitoring and review process for GSK239512. Measures will be taken to ensure that unblinded information is not provided to the site personnel, subjects or any GSK and vendor staff involved in the management of the study sites and subject data. In addition, a formal Interim Analysis will be conducted, in accordance with Protocol Section 8 and the iSRC charter, to assess the characteristics of the remyelination signal that may be detectable at the end of the study. This will include an assessment of the probability of achieving the primary study objective and allows for the possibility of stopping the study for futility.

Protocol waivers or exemptions are not allowed with the exception of immediate safety concerns. Therefore, adherence to the study design requirements, including those specified in the Protocol Time and Events Table, are essential and required for study conduct.

Supplementary study conduct information not mandated to be present in this protocol is provided in the accompanying Study Procedures Manual (SPM). The SPM will provide the site personnel with administrative and detailed technical information that does not impact subject safety.

Figure 1 Study Design with Titration

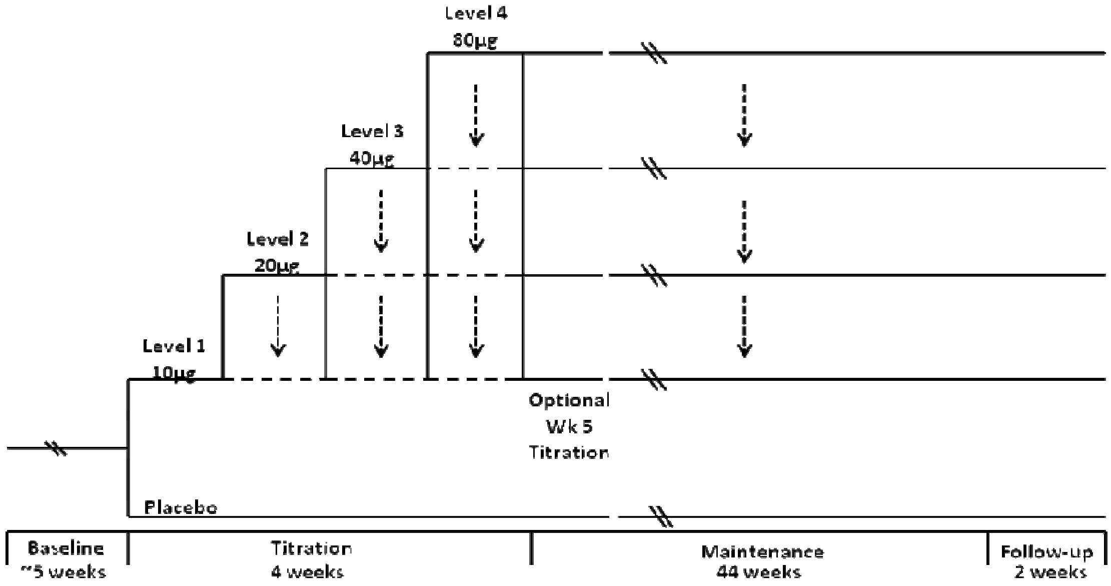

4. PLANNED ANALYSES

4.1. Interim Analyses

In addition, a formal Interim Analysis will be conducted when approximately half the subjects have Week 42 MRI data available to assess the characteristics of the remyelination signal that may be detectable at the end of the study. This will include an

assessment of the probability of achieving the primary study objective and allows for the possibility of stopping the study for futility (see Section 5 for details on stopping guidelines).

4.2. Final Analysis

Once all subjects have completed the study, all data is in house, all data queries have been resolved and protocol violators have been determined, the data will be frozen, unblinded and all data displays described in this analysis plan will be generated. Details of final efficacy analyses are provided in Section 11.

The quality control (QC) of the tables, figures and listings generated for the statistical reporting and analysis of this study is the responsibility of the Biostatistics and Programming Team (BPT). While it is anticipated that the BPT will work from a fully quality assured database, any data anomalies discovered during final analysis will be communicated to the Data Management and Clinical groups. The BPT will perform a QC review of the results prior to release in accordance with the SOP, SOP_55411, (Review/Quality Control of Statistics and Programming Phase I-IV Results).

5. SAMPLE SIZE CONSIDERATIONS

5.1. Sample Size Assumptions

The following assumptions have been made in the design of this study:

- The treatment effect for both co-primary endpoints will be expressed as effect sizes and are therefore assumed to have distributions of mean 0 and standard deviation 1. The target effect size for this study is 0.5. Based on unpublished data in post-mortem brains for SPMS subjects, it is believed that an effect size of 0.5 is approximately equivalent to a 50% increase in remyelination compared to the natural remyelination process that would be expected in the placebo group.
- The correlation between the two co-primary endpoints is 0.5. The distribution of the co-primary endpoints is described by a Bivariate Normal Distribution.
- Analysis will be performed at the subject level with the estimation of remyelination within each lesion averaged for each subject. It is assumed that each subject will have at least 1 lesion that contributes to the analysis for both endpoints.
- An interim analysis will be performed when approximately half the subjects have Week 42 MRI data available. The study will be considered as potentially futile if both co-primary endpoints have a $PP(\Delta>0)<30\%$ at the time of the interim analysis.
- A signal of efficacy will be declared if the posterior probability the true effect size (GSK239512-placebo) is greater than zero is more than 80% ($PP(\Delta>0)>80\%$) for both co-primary endpoints. An efficacy signal will also be

considered observed if one co-primary endpoint has a PP(Δ>0)>80% and the other co-primary endpoint has a PP(Δ>0)>70%. A negative study will be declared if the posterior probability that the true effect size (GSK239512-placebo) is greater than zero is less than 70% (PP(Δ>0)<70%). A negative study will also be considered to have been observed if one co-primary endpoint has a PP(Δ>0)<70% and the other co-primary endpoint has a PP(Δ>0)<80%. Otherwise there is a grey area. Table 2 provides a summary of the potential outcomes using a color-coded approach.

- Given the exploratory nature of the study, no formal Type I and Type II Error rates are defined and no adjustments for multiplicity are required.

Based on the above assumptions, a simulation approach has been employed to investigate the chance of stopping for futility, and correctly or incorrectly detecting a signal of efficacy on the co-primary endpoints, based on 10,000 simulations. Based on the outcome of these simulations 114 subjects will be randomized to achieve 100 subjects with at least one post-baseline MRI. Figure 2 shows the chance of stopping for futility or declaring a positive "Go" or negative "Stop" study with 50 evaluable subjects/arm for a range of unknown true effect sizes and Table 2 shows the chances of each making each potential decision in the study based on each co-primary endpoint for true unknown effect sizes of 0 and 0.5 for the study sample size.

Figure 2 Chance of Stopping for Futility at the Interim Analysis (PP(Δ>0)<30%), Positive "Go" Result at Study End (PP(Δ>0)>80%) and Negative "Stop" Result at Study End (PP(Δ>0)<70%)

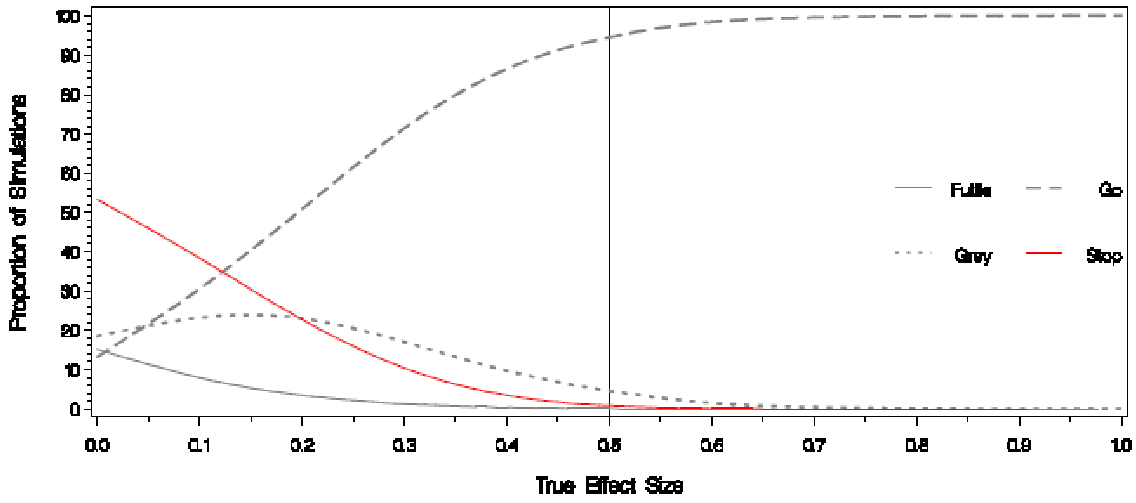

Table 2 Chance of Stopping for Futility at the Interim Analysis (PP(Δ>0)<30%), Positive "Go" Result at Study End (PP(Δ>0)>80%) and Negative "Stop" Result at Study End (PP(Δ>0)<70%)

Treatment Difference for both Co-Primary Endpoints	Percentage of Trials in this scenario (%)		Decision for Co-Primary Endpoint 2			
			Futile	Go	Grey	Stop
0	Decision for	Futile	15.39%	0.00%	0.00%	0.00%

Treatment Difference for both Co-Primary Endpoints	Percentage of Trials in this scenario (%)		Decision for Co-Primary Endpoint 2			
			Futile	Go	Grey	Stop
	Co-Primary Endpoint 1	Go	0.00%	8.01%	2.75%	8.21%
		Grey	0.00%	2.64%	1.40%	5.66%
		Stop	0.00%	8.44%	5.63%	41.87%
0.5	Decision for Co-Primary Endpoint 1	Futile	0.06%	0.00%	0.00%	0.00%
		Go	0.00%	90.45%	1.97%	2.31%
		Grey	0.00%	1.89%	0.24%	0.22%
		Stop	0.00%	2.15%	0.29%	0.42%

In this study with 100 subjects, the selected criterion for efficacy detection is the posterior probability the true effect size (GSK239512-placebo) is greater than zero is more than 80%. From Figure 2 and Table 2 we can see that for this efficacy detection (i.e. PP(Δ>0)>80%) with 50 subjects per arm, if the true effect is 0.5 for both co-primary endpoints we will detect a signal of efficacy in approximately 94% of trials, and declare a negative "Stop" signal in ~41% of trials. Less than 1% of studies would be stopped for futility at the interim analysis. If the true effect is 0 for both co-primary endpoints (i.e. GSK239512 is no better than placebo) then the studies would incorrectly detect a signal in ~13% of trials. If the true effect for both co-primary endpoints was 0 the study would be considered futile for its primary objective in ~16% of trials; and if the true effect for both co-primary endpoints was 0 the study would be considered negative for its primary objective at the study end in ~53% of trials.

5.2. Sample Size Sensitivity

As discussed in Section 5.1, it is possible that not all subjects will have an analyzable GdE lesion while participating in the study. It is estimated that 40-60% of subjects will have a GdE lesion that can be assessed for remyelination. It is believed that based on non-published data [personal communication, [redacted] the prevalence of delta-MTR lesions will be higher than the prevalence of GdE lesions.

Therefore, it is possible that not all subjects will be able to contribute to the analyses for either co-primary endpoint. Figure 3 demonstrates the chance of stopping for futility, or declaring a positive or negative study, with 30 evaluable subjects/arm for a range of unknown true effect sizes.

Figure 3 Chance of Stopping for Futility or Declaring a Positive or Negative study with 30 evaluable subjects/arm

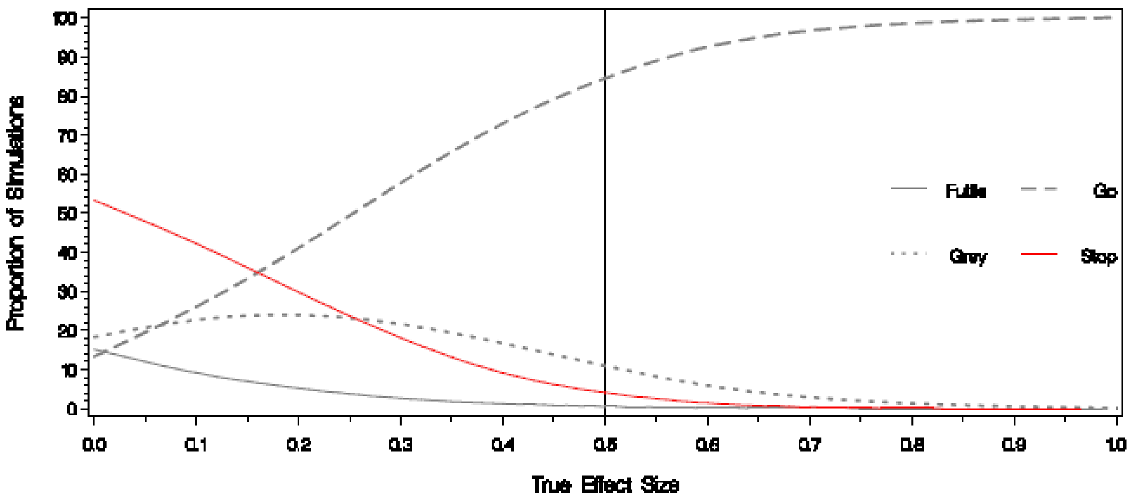

It can be seen that with fewer subjects being evaluable for the GdE lesion co-primary endpoint reduces the probability of declaring a positive study and slightly increases the chances of stopping the study at the interim analysis or declaring a negative study for the target effect size of 0.5. For this efficacy detection (i.e. PP(Δ>0)>80%) with 30 subjects per arm, if the true effect is 0.5 for both co-primary endpoints we will detect a signal of efficacy in approximately 84% of trials, and declare a negative signal in ~4% of trials. Less than 1% of studies would be stopped for futility at the interim analysis. If the true effect is 0 for both co-primary endpoints (i.e. GSK239512 is no better than placebo) then with 30 subjects per arm the outcomes are similar in that studies would incorrectly detect a signal in ~13% of trials. If the true effect for both co-primary endpoints was 0 the study would be considered futile for its primary objective in ~15% of trials; and if the true effect for both co-primary endpoints was 0 the study would be considered negative for its primary objective at the study end in ~53% of trials.

Table 3 shows the probability of each study outcome varying the correlation coefficient between the two co-primary endpoints for the sample size of 50 subjects per group. It can be seen that when the true treatment effect size is 0, the stronger the correlation between the two co-primary endpoints the more likely the study would either stop for futility at the interim analysis or declare a negative study. For a true effect size of 0.5 there is little impact on the outcomes of the study with changing correlation coefficients. As the correlation increases, there is a slight increase in the probability of a positive signal being observed.

Table 3 Assessment of Impact of Correlation Coefficients on Study Outcomes

Treatment Difference for both Co-Primary Endpoints	Percentage of Trials in this scenario (%)		Decision for Co-Primary Endpoint 2			
			Futile	Go	Grey	Stop
Correlation Coefficient: 0.2						
0	Decision for Co-Primary Endpoint 1	Futile	11.27%	0.00%	0.00%	0.00%
		Go	0.00%	5.31%	2.22%	11.44%
		Grey	0.00%	2.18%	1.20%	6.37%
		Stop	0.00%	11.71%	6.59%	41.71%
0.5	Decision for Co-Primary Endpoint 1	Futile	0.02%	0.00%	0.00%	0.00%
		Go	0.00%	89.80%	2.12%	2.81%
		Grey	0.00%	2.14%	0.10%	0.11%
		Stop	0.00%	2.57%	0.13%	0.20%
Correlation Coefficient: 0.5						
0	Decision for Co-Primary Endpoint 1	Futile	15.39%	0.00%	0.00%	0.00%
		Go	0.00%	8.01%	2.75%	8.21%
		Grey	0.00%	2.64%	1.40%	5.66%
		Stop	0.00%	8.44%	5.63%	41.87%
0.5	Decision for Co-Primary Endpoint 1	Futile	0.06%	0.00%	0.00%	0.00%
		Go	0.00%	90.45%	1.97%	2.31%
		Grey	0.00%	1.89%	0.24%	0.22%
		Stop	0.00%	2.15%	0.29%	0.42%
Correlation Coefficient: 0.8						
0	Decision for Co-Primary Endpoint 1	Futile	20.35%	0.00%	0.00%	0.00%
		Go	0.00%	12.13%	2.98%	3.86%
		Grey	0.00%	2.97%	1.89%	4.73%
		Stop	0.00%	4.41%	4.18%	42.50%
0.5	Decision for Co-Primary Endpoint 1	Futile	0.34%	0.00%	0.00%	0.00%
		Go	0.00%	91.90%	1.51%	1.32%
		Grey	0.00%	1.43%	0.48%	0.42%
		Stop	0.00%	1.19%	0.43%	0.98%

5.3. Sample Size Re-estimation

During the instream reviews (Protocol Section 8.3.4), an assessment of the GdE lesion rate and the ability of the study to detect effects of GSK239512 where $PP(\Delta>0)>80\%$ will be performed. If the GdE lesion occurrence rate is lower than expected an increase in sample size or changes in the inclusion/exclusion criteria will be considered to increase the likelihood that subjects recruited will contribute lesions to the primary analysis (Protocol Section 8.3.4).

6. ANALYSIS POPULATIONS

Five populations are defined for this study:

Screen Failure population: This will consist of all subjects who failed Screening and will be used for Screening Failure displays only.

Randomised population: This will consist of all subjects randomised to study medication.

Intent-to-treat (ITT) population (Safety Population): This will consist of all randomised subjects who have taken at least one dose of study medication. This population will be used for the analysis of all efficacy and safety data. For summary tables and figures it will be denoted as “Intent-to-Treat/Safety Population” and Table 6.01 will be footnoted to state that the 2 populations are defined the same. Throughout this RAP, this population will be referred to as the ITT Population.

PK-concentration population: This will include all subjects for whom a pharmacokinetic sample was obtained and analysed.

Pharmacogenetics Population: This will include all subjects in the ITT population who consented to providing a genetics sample, provided a sample and did not withdraw their consent for genetic testing.

6.1. Analysis Datasets

The Observed Case (OC) dataset is defined for all endpoints and will contain all the available change from baseline responses for each subject at each visit, without missing values being estimated or data carried forward other than as described in Section 9.2.8.2.

6.1.1. Non-MTR MRI Endpoints

For non-MTR MRI endpoints two additional datasets are defined.

- The **All Evaluable Scans (AES)** dataset which includes all evaluable on-treatment MRI scans for each subject, thus allowing estimates of lesion rates to be calculated across the entire Treatment Phase. A scan is evaluable if NeuroRx, the vendor who is reading the MRI scans, indicate it as evaluable and it was not performed between 1 and 7 days of the conclusion of steroid dosing for relapse.

An MRI scan will be considered evaluable if steroids were administered on the same day as the MRI scan was performed.

- The **Imputed Missing Values (IMV)** dataset will be used for a sensitivity analysis of MRI parameters only: in the IMV dataset, missing values for an individual subject will be estimated from the other available observations for only that subject, allowing estimates of treatment effect to be made with the complete study population. Further details on the derivation of both datasets are provided in Section 9.2.8.2.

7. TREATMENT COMPARISONS

The primary comparisons of interest in this study are the effects of GSK239512 relative to placebo administered adjunctively to stable Background MS Disease Modifying Treatment (Avonex or Copaxone) on the co-primary endpoints as specified in Section 2.2. No formal hypothesis testing will be performed and no Type I Error Rate is nominated for this study. Treatment differences will be estimated, standardised Effect Sizes will be derived as the treatment difference divided by the standard deviation (estimated as the between subject variability from the analysis model) of the treatment difference averaged across visits/Relative MRIs (both with associated 90% confidence intervals) and posterior probabilities that the difference between treatment groups is greater than zero will be presented.

7.1. Data Display Treatment and Other Sub-group Descriptors

Treatment groups will be identified in the data displays as ‘Placebo’ and ‘GSK239512‘. Specific displays will also be provided by dose of GSK239512 and will be identified as ‘GSK239512 10ug’, ‘GSK239512 20ug’, ‘GSK239512 40ug’ and ‘GSK239512 80ug’ respectively.

Data displays presented for all subjects and by Background Disease Modifying Therapy will be labelled: “Stratum: All Subjects”, “Stratum: Background Disease Modifying Therapy – Avonex” and “Stratum: Background Disease Modifying Therapy – Copaxone”

8. GENERAL CONSIDERATIONS FOR DATA ANALYSES

All programming will be performed using SAS version 9.1.3 or later in a Linux environment.

8.1. Multicentre Studies

Subjects have been recruited into this study from centres across Bulgaria, Canada, Czech Republic, Germany, Spain, Sweden, Ukraine and United Kingdom. Low numbers of subjects were recruited at some centres and therefore centres will be pooled within each country. As some countries recruited few subjects, country groups will be further combined as follows to create 2 groups: Ukraine and Other (Bulgaria, Canada, Czech Republic, Germany, Spain, Sweden and United Kingdom). The effect of country group

variability will be assessed as an exploratory covariate for the co-primary endpoints; See Section 11.1.2

Throughout this document any references to a centre effect or grouping of centres will be denoted as “country”.

Approximately 35% of subjects in this study have been recruited in the Ukraine. The number of subjects in each country group will be small and as such, any assessment of a treatment by country interaction would have a high probability of showing a false result (positive or negative). Hence formal hypothesis testing of the treatment by country interaction term will not be conducted. Inter-country variability may be investigated by reviewing summary statistics of the co-primary efficacy parameters by country group; graphical displays will also be produced. Details are provided in Section 8.3.

8.2. Other Strata and Covariates

The study design includes stratification based on the MS background disease modifying therapy the subject is receiving (Avonex or Copaxone). All statistical analyses will include a factor for Background Disease Modifying Therapy. Statistical analyses defined in Section 11 will define the terms that will be included in the statistical analysis models. As the study is relatively small, if analysis models do not converge covariates may be removed from the model or different factors of a covariate may be combined to allow the statistical analysis model to converge. If this occurs, details will be provided in the CSR.

The effects of following covariates will be assessed in an exploratory sensitivity analysis of the co-primary endpoints and some key secondary endpoints as appropriate based on initial results:

- Gender
- Age
- Country
- Presence of gadolinium-enhancing lesions on Screening MRI Scan (Presence/Absence)
- Baseline EDSS Score (≤2.5/≥3)
- Baseline T2 Lesion MTR
- Normalised Brain Volume at Screening\
- Number of Relapses in previous 24 months before Screening (<2/≥2)
- Duration of disease (years since diagnosis) ≤6/>6 years
- Baseline EDSS Modified Visual Score (≤1/≥2) [CogState endpoints only]

- Baseline Body Mass Index (BMI)
- Lesion size [MTR Co-primary endpoints only]

Continuous covariates will be centred such that each value will have the baseline mean for the ITT population subtracted from the actual value.

8.3. Examination of Subgroups

Two subgroups will be investigated in this study; subjects receiving “Avonex” and subjects receiving “Copaxone” as their background MS disease modifying therapy. These subgroups will contain a small number of subjects and the potential for false results is not controlled in the study. Each subgroup will be investigated for each co-primary endpoint and CogState assessments; other secondary endpoints may be investigated further if the effect for the covariate is significant in statistical models.

In addition, at the request of the Spanish Regulatory Agency, if a substantial number of subjects do not titrate up to Dose Level 4, sensitivity analyses of the co-primary endpoints and key secondary endpoints will also be analysed assessing Dose Level as a categorical covariate. For the purposes of conducting these analyses, a substantial proportion of subjects not maintaining Dose Level 4 of ≥30% will be the cut-off for this analysis as is in line with previous studies in this compound. Therefore, if more than 70% of subjects stay on Dose Level 4 through the Maintenance Period, these analyses will not be conducted

8.4. Multiple Comparisons and Multiplicity

This is an exploratory study with no hypothesis testing such that no adjustment for multiplicity is required.

8.5. Genetic Markers

Genetic markers are not planned to be investigated in this reporting effort. Any genetic analyses will be described in a separate analysis plan authored by the GSK Genetics Group, or designate.

9. DATA HANDLING CONVENTIONS

9.1. Premature Withdrawal and Missing Data

A subject is considered to have completed the study if they complete the 48 week treatment period and attend the follow-up visit specified in the protocol to be approximately 2 weeks after the Week 48 visit. For subjects that do not complete the study, details of how to deal with missing data are provided in Section 6.1 and Section 9.2.

9.2. Derived and Transformed Data

9.2.1. Baseline Data

For safety and efficacy data baseline is considered to be the subject's last available assessment prior to initiation of study medication unless otherwise stated. Change from baseline for post-baseline records will be calculated by subtracting the baseline value from the post-baseline value.

9.2.2. Demographic Data

Body Mass Index (BMI) at screening will be calculated using the height and weight at that visit:

$$\text{Body Mass Index (kg/m}^2\text{)} = \frac{\text{Weight (kg)}}{\text{Height (m)}^2}$$

Age (in years) will be determined at the Screening visit using the date of the Screening visit as recorded in the eCRF. Only Year of Birth is collected in the eCRF such that the birth date will be imputed as 30 June of the year entered in the eCRF.

9.2.3. Study Day Definition

Study day for the assessment date of interest should be calculated relative to the first dose date of double-blind study medication using the appropriate formula below:

1) If the assessment date of interest is on or after the first dose date of double-blind study medication:

   Study day = assessment date – date of first dose of double-blind study medication + 1

2) If the assessment date of interest is before the first dose date of double-blind study medication:

   Study day = assessment date – first dose date of double-blind study medication

9.2.4. Defining the First Dose Date

The first dose date of double-blind medication (*MSTSTDT* variable in MSTONE) is defined as the earliest start date in the Exposure dataset with a *VISITNUM* >=20.

If the number of tablets taken is equal to 0, the start date for this row of study medication should be ignored.

The first double-blind dose date will be used to populate the variable DMREFDT.
This date is used for study day calculations.

9.2.5. Defining the Last Dose Date

The last dose date of double-blind medication (*MSTENDT* variable in MSTONE) is defined as:

If the treatment stop date is present on the last row of data, this should be used as the stop date of study medication.

If the treatment stop date is missing on the last row of data but a start date is present then the start date should be used as the stop date of study medication.

In the case where no start or stop dates are known on the last dosing record then the above process is repeated on previous sets of dosing records until a stop date of study medication is obtained. Any dosing records where it cannot be determined that study medication was taken will be ignored.

It should be noted that the Treatment Phase will end 3 days after the last dose of double-blind study medication.

9.2.6. Defining Titration and Maintenance Periods

Titration in the study is planned to take 4 weeks but if an additional week of titration is required then the Titration Period can be adapted from 4 weeks to 5 weeks.

The Titration Period starts on the first dose date of double-blind medication (MSTSTDT variable in MSTONE) as defined in Section 9.2.4.

If the subject undergoes a 4-week Titration Period (defined as *OPTW5TIT* variable in *OPTTITR* DM Dataset being "N") then:

- The Maintenance Period is considered to have started on the start date recorded in the eCRF for Week 4 (using *EXSTDT* variable in *EXPOSURE* DM Dataset).

If the subject undergoes a 5-week Titration Period (defined as *OPTW5TIT* variable in *OPTTITR* DM Dataset being "Y") then:

- The Maintenance period is considered to have started on the start date recorded in the eCRF for Week 5 (using *EXSTDT* variable in *EXPOSURE* DM Dataset).

The Titration Period is considered to have ended the day before the start of the Maintenance Period. However, if no Maintenance Period data is available, the Titration period will be considered to have ended at the latest available study medication dosing date available and no Maintenance Period start and stop dates will be defined for the subject.

The Maintenance Period ends at the same time as the Treatment Phase ends as defined in Section 9.2.5.

9.2.7. MRI – MTR Data

MTR data will be provided to GSK in a format of one row of data per subject per lesion per MRI assessment. A value of 1 is considered to be a normal White Matter MTR value, a value of 0 is considered to be a normal Grey Matter MTR Value. Lesions can occur at different visits in the study, to transform lesions to the same scale additional timepoint variables will be derived. For each unique lesion identified by NeuroRx, the MRI on which it is identified will be set to be "Lesion Day 0" and the MRI will be defined as the "Reference MRI". Therefore for a subject with more than one lesion they will have more than one "Reference MRI" and more than one "Lesion Day 0"; i.e. one set per lesion identified. For all other MTR MRI scans for each lesion their "Lesion Day" will be calculated as MRI date minus Reference MRI date (plus 1 day for post Reference MRI scans) and MRIs will be identified relative to the "Reference MRI" such post lesion MTR MRI scans will be identified as "Post Lesion Scan 1", "Post Lesion Scan 2" etc and MTR MRI scans prior to the "Reference MRI" will be identified as "Pre Lesion Scan 1", "Pre Lesion Scan 2" etc as shown in Table 4 for a subject with a gadolinium-enhancing lesion observed at the Week 18 MRI assessment. A lesion phase ID variable categorising "Pre-Lesion", "Reference MRI" and "Post-Lesion" will also be defined.

Table 4 Worked Example for Defining MTR Scans

Study MRI	Lesion ID	Lesion Day	Relative MRI	Lesion Phase
Screening	GdE_Wk18	-126	Pre-Lesion MRI 3	Pre-Lesion
Week 6	GdE_Wk18	-84	Pre-Lesion MRI 2	Pre-Lesion
Week 12	GdE_Wk18	-42	Pre-Lesion MRI 1	Pre-Lesion
Week 18	GdE_Wk18	0	Reference MRI	Reference MRI
Week 24	GdE_Wk18	42	Post-Lesion MRI 1	Post-Lesion
Week 30	GdE_Wk18	84	Post-Lesion MRI 2	Post-Lesion
Week 36	GdE_Wk18	126	Post-Lesion MRI 3	Post-Lesion
Week 42	GdE_Wk18	168	Post-Lesion MRI 4	Post-Lesion
Week 48	GdE_Wk18	210	Post-Lesion MRI 5	Post-Lesion

The change in MTR from the Reference MRI is calculated as the non-Reference MRI MTR Value minus the Reference MRI MTR Value. The change in MTR for Post-Lesion MRI scans from Pre-Lesion is the change in each Post-Lesion MRI scans from the average Pre-Lesion MRI scans.

All these derivations are conducted at the lesion level. As a potential for a sensitivity analysis and to reduce the variability the MTR Value at each MRI and the change from

Reference MRI MTR Value will be derived at the subject level by taking the average (mean) of all lesions (gadolinium-enhancing lesions and delta MTR lesions separately). In addition, the mean of all Pre-Lesion MRI scan MTR Values and Post-Lesion MRI Scan MTR Values will be derived at the lesion level and the subject level.

As there is expected to be increased variability in MTR Values around the time of lesion occurrence sensitivity analyses to assess the impact of inclusion of MTR data close to lesion occurrence may be required to fully assess the robustness of the analyses and to be able to fully interpret the study. At the time of writing the protocol it was advised that MTR data from MRI scans within 12 weeks of a lesion being observed should be excluded due to increased variability. This analysis is defined as the primary dataset as per the study protocol. Additional sensitivity analyses are also included in Table 5 which will allow additional investigations of these novel endpoints to be conducted. For all definitions, consideration will be made as to whether there must be a minimum of 2 MRIs pre-lesion and post-lesion as sensitivity analyses.

Table 5 Summary of Sensitivity Analyses Definitions

Analysis ID	Pre-Lesion MRI Scans to Exclude	Post-Lesion MRI Scans to Exclude
1 [Protocol-specified analysis]	Within 70 days of Reference MRI Scan	Within 70 days of Reference MRI Scan
2	Within 28 days of Reference MRI Scan	Within 70 days of Reference MRI Scan
3	No scans excluded	No scans excluded
4	Within 28 days of Reference MRI Scan	Within 28 days of Reference MRI Scan
5	Within 70 days of Reference MRI Scan	Within 28 days of Reference MRI Scan

Note, for all sensitivity analyses specified in Table 5 the average MTR Values should be derived as specified in this section at the lesion and subject level for both gadolinium-enhancing lesions and delta-MTR lesions.

9.2.8. MRI – Other MRI Endpoints

9.2.8.1. Conventional MRI endpoints from Central Reader

The following MRI endpoints will be collected by NeuroRx, the central MRI reader and sent to GSK in the *MRIRES* DM Dataset for each visit (Weeks 6, 12, 18, 24, 30, 36, 42 and 48) unless otherwise specified:

- Number of gadolinium-enhancing lesions (Also reported at Screening)
- Number of new gadolinium-enhancing lesions
- Number of new and enlarging T2 lesions

- Number of new unenhancing T1 lesions
- Number of unique actions lesions (new GdE lesions and new enlarging T2 lesions not associated with Gd)
- Number of new gadolinium-enhancing lesions evolving into black holes at end of study from each on-treatment MRI
- Normalised brain volume (Screening only*)
- Whole brain atrophy volume change relative to Screening (Week 48 only)
- White matter volume (Screening only*)
- White matter volume change relative to Screening (Post Screening MRIs only)
- Grey matter volume (Screening only*)
- Grey matter volume change relative to Screening (Post Screening MRIs only)

* [redacted] Instead, Week 6 has been used as the scan that will be used for assessing relative changes at other study visits. Changes from Screening as described in this RAP will mean changes from Week 6 for this single subject.

In addition, the number of gadolinium-enhancing lesions observed on the Screening MRI will also be databased.

9.2.8.2. MRI endpoints to be Derived Programmatically

As defined in Section 6.1.1, 3 datasets are defined for conventional MRI endpoints. The Observed Case (OC) dataset is the data reported by NeuroRx and will be used in listings and for summary statistics for actual MRI lesion counts tables only. The All Evaluable Scans (AES) and Imputed Missing Values (IMV) datasets will be used for cumulative MRI counts for summary statistics and statistical analyses; they will also be listed. The AES dataset is derived by carrying down cumulative counts and number of evaluable scans from previous visits to ensure all patients with at least 1 MRI prior to that visit are included in the summary table as demonstrated in Table 6. The IMV dataset is derived by imputing missing MRI scan lesion counts with the average number of lesions for non-missing MRIs for that subject as demonstrated by Table 6. These derivations will be conducted for the following endpoints only:

- Number of new gadolinium-enhancing lesions
- Number of new and enlarging T2 lesions
- Number of new unenhancing T1 lesions

- Number of unique actions lesions (new GdE lesions and new enlarging T2 lesions not associated with Gd)

- Number of new gadolinium-enhancing lesions evolving into black holes at end of study from each on-treatment MRI

Table 6 Worked Example for Deriving Lesion Counts

		Observed Case		All Evaluable Scans		Imputed Missing Values	
Week	Observed Lesion Count	Cum. Count	No. of Scans	Cum. Count	No. of Scans	Cum. Count	No. of Scans
Example 1: Missing MRI at Week 24							
6	1	1	1	1	1	1	1
12	2	3	2	3	2	3	2
18	0	3	3	3	3	3	3
24	Missing	Missing	Missing	3	3	4	4
30	1	4	4	4	4	5	5
36	2	6	5	6	5	7	6
42	1	7	6	7	6	8	7
48	0	7	7	7	7	8	8
Example 2: Subject Withdrawal prior to Week 24 MRI							
6	1	1	1	1	1	1	1
12	2	3	2	3	2	3	2
18	0	3	3	3	3	3	3
24	Missing	Missing	Missing	3	3	4	4
30	Missing	Missing	Missing	3	3	5	5
36	Missing	Missing	Missing	3	3	6	6
42	Missing	Missing	Missing	3	3	7	7
48	Missing	Missing	Missing	3	3	8	8

The normalised brain volume, white matter volume and grey matter volume at each post Screening MRI will be derived using the following formula:

Post Baseline Volume=Baseline Volume plus (Baseline Volume x Relative Change Post Baseline)

The Change from Baseline in each volume is then derived as:

Change from Baseline=Post Baseline Volume minus Baseline Volume

All Brain Volume measurements have been sent to GSK in mm^3 units. Reporting will be performed using cm^3 and will be converted by dividing the data provided by NeuroRx by 1,000.

9.2.9. CogState Battery

The CogState battery used in this study consists of 7 'tasks', for which multiple outcomes 'measures' are collected. As defined in Table 7 for each task, an outcome 'measure' is defined for each 'task' and that 'measure' is used in the summary statistics/statistical analyses for that 'task', as applicable, and used in the derivation of Domain Scores and the CogState Battery Total Score. This measure (denoted as X) is 'standardised' by subtracting the overall ITT population mean of the derived baseline values (m_{bt}) and then dividing by the overall ITT population standard deviation of the derived baseline values (σ_{bt}) . If an improvement in an individual task is defined as a negative change from baseline, this effect size will be multiplied by -1 (see Table 7). A **standardised** Task Score is therefore the direction-adjusted, standardised measure and at each assessment is calculated as follows:

$$\frac{-1 * (X_{jt} - \overline{m}_{bt})}{\sigma_{bt}}$$

Where b=baseline, t=task and j= week 12 or 24 NB: the -1 is only applicable where a lower score indicates an improvement.

Individual tasks which are flagged by CogState as not having been completed will not be used in the derivation of the standardised 'task' scores, and consequently any composite scores or be analysed. This step will be completed before any derivations of the data are conducted. This data will be listed only. If >10% of tasks are defined as integrity failures by CogState, a post-hoc sensitivity analysis may be conducted to assess the impact of these integrity failures on the study results.

The baseline is derived as the average of the second CogState Battery assessment conducted at the Screening visit and the Baseline (Week 0) CogState Battery assessment. If either assessment is missing then the derived baseline will be the non-missing assessment. If both assessments are missing then the derived baseline will also be missing.

Table 7 Measures for Individual CogState Tasks

Task	Short Task Label for reporting	Cognitive Testing Domain	Task Code on COGTEST dataset	Primary Measure	Measure Code on COGTEST dataset	Multiply by -1?
ISLT task	ISLT	Verbal Learning	23	Number of correct responses	COR	No
ISLT delayed recall	ISLT-R	Executive Function / Spatial Problem Solving	15	Number of correct responses	COR	No
Groton Maze	GML	Psychomotor	24	Total	TER	Yes

Task	Short Task Label for reporting	Cognitive Testing Domain	Task Code on COGTEST dataset	Primary Measure	Measure Code on COGTEST dataset	Multiply by -1?
Learning Test		Function / Speed of Processing		Number of Errors		
Detection	DET	Visual Attention / Vigilance	19	Speed of Performance (log msec)	LMN	Yes
Identification	IDN	Visual Learning & Memory	18	Speed of performance (log msec)	LMN	Yes
One-Card Learning	OCL	Attention / Working Memory	42	Accuracy of Performance	ACC	No
One-Back	OBL	Verbal Learning and Memory	20	Speed of Performance (log msec)	LMN	Yes

Table 8 summarises the 'tasks' to be included in each Composite Domain. Each Composite Domain Score and the CogState Battery Total Score (uses all 'tasks') is derived by taking the average of all 'standardised' directionally-adjusted 'task' scores. In order to calculate any Composite Domain Score or the CogState Battery Total Score there must be at least 2 'tasks' that have non-missing data to be used in the calculation. If this criterion is not met then a subject will have a missing Composite Domain or CogState Battery Total Score for that visit.

Table 8 Summary of CogState Battery Composite Domain Tasks

Domain	Task
Executive Function	Groton Maze Learning Test (GMLT)
	One-Back Test (OBT)
Memory	International Shopping List Task – Immediate Recall (ISLT)
	International Shopping List Task – Delayed Recall (ISLT-R)
	One Card Learning (OCL)
Attention	Detection
	Identification

If the amount of missing data at a particular visit means that various composite scores cannot be calculated but there is another assessment which slots to the same visit from which you can calculate more composite scores, then the assessment with the largest number of composite scores available will be used in all summaries. Data from both

visits would be listed. CogState have provided age-controlled summary statistics for normative data for each CogState Battery task included in this study (see Section 16 for file location on BPT shared area network drive). Subjects recruited into this study were not required to have any specific cognitive impairment. To enable an assessment of the potential baseline impairment of subjects entering this study, for the derived Baseline assessment the score for each task will be compared to the normative data using the following formula:

Baseline Impairment = (Baseline Value minus Normative Mean) divided by Normative Standard Deviation (SD)

Negative values will suggest that a subject's test result at Baseline is below that of a normal population and positive values will indicate a Baseline result that is above average for a normal population for that task. A categorical variable for each task will then be defined expressing the Baseline Value relative to the Normative data: ≥2 SDs,<2 and ≥1 SD, <1 ≥0.5 SDs and <0.5 SDs above and below the Normative Mean.

9.2.10. MSQoL54

The MSQoL54 is a tool for assessing health related quality of life outcomes in studies of patients with MS [Vickrey, 1995]. It involves 54 questions for the subject to respond to; 52 items are used to derive two summary composite scores composed of 12 subscales and there are also 2 individual items. The subscales are: physical function, role limitations-physical, role limitations-emotional, pain, emotional well-being, energy, health perceptions, social function, cognitive function, health distress, overall quality of life, and sexual function. The summary scores are the physical health composite summary and the mental health composite summary. The single item measures are satisfaction with sexual function and change in health.

The 'response' to each question is transformed into a 'score' based on Table 9. Subscale scores are then derived by averaging (means) the 'responses' for each question in that subscale ('divisor' defined in Table 9). If a 'response' is missing then the denominator is corrected to exclude any questions with a missing 'response'. A minimum of one non-missing 'response' is required to derive a sub-scale score.

Table 9 Summary of Response Scoring for MSQoL54

Item Numbers#	Response						
	1	2	3	4	5	6	7
Subscale: Physical Health (Divisor=10)							
3-12	0	50	100	N/A	N/A	N/A	N/A
Subscale: Role Limitations due to Physical Problems (Divisor=4)							
13-16	0	100	N/A	N/A	N/A	N/A	N/A
Subscale: Role Limitations due to Emotional Problems (Divisor=3)							
17-19	0	100	N/A	N/A	N/A	N/A	N/A
Subscale: Pain (Divisor=3)							
21	100	80	60	40	20	0	N/A

Item Numbers[#]	Response					
22, 52	100	75	50	25	0	N/A	N/A
Subscale: Emotional Well-Being (Divisor=5)							
24, 25, 28	0	20	40	60	80	100	N/A
26, 30	100	80	60	40	20	0	N/A
Subscale: Energy (Divisor=5)							
23, 27, 32	100	80	60	40	20	0	N/A
29, 31	0	20	40	60	80	100	N/A
Subscale: Health Perceptions (Divisor=5)							
1, 35, 37	100	75	50	25	0	N/A	N/A
34, 36	0	25	50	75	100	N/A	N/A
Subscale: Social Function (Divisor=3)							
20, 51	100	75	50	25	0	N/A	N/A
33	0	25	50	75	100	N/A	N/A
Subscale: Cognitive Function (Divisor=4)							
42-45	0	20	40	60	80	100	N/A
Subscale: Health Distress (Divisor=4)							
38-41	0	20	40	60	80	100	N/A
Subscale: Sexual Function (Divisor=4)							
46-49	100	66.7	33.3	0	N/A	N/A	N/A
Change in Health*							
2	100	75	50	25	0	N/A	N/A
Satisfaction with Sexual Function*							
50	100	75	50	25	0	N/A	N/A
Subscale: Overall Quality of Life (Divisor=2)							
53	Multiply response by 10						
54	0	16.7	33.3	50	66.7	83.3	100

[#] Divisor for each subtotal denotes the number of items for each subscale assuming no missing data for the subscale. The divisor should be adjusted for missing data such that the divisor becomes the number of items for which there is non-missing data. If all items are missing the subscale score is also missing.
* Single item measures that are not defined as an MSQoL54 Subscale

Two Composite Scores are defined for MSQoL54 using the subscale scores defined in Table 10. The MSQoL54 Physical Health Composite Score and the MSQoL54 Mental Health Composite Score are calculated as a weighted-sum using the subscale scores and the weights defined in Table 10. If a subscale score is missing then it will not contribute to the Composite Score calculation (in effect, it is treated as a score of 0).

Table 10 Derivation of MSQoL54 Composite Scores

Composite Score	Subscale	Weight for Composite
Physical Health	Physical Function	0.17
	Health Perceptions	0.17
	Energy	0.12

Composite Score	Subscale	Weight for Composite
	Role Limitations – Physical	0.12
	Pain	0.11
	Sexual Function	0.08
	Social Function	0.12
	Health Distress	0.11
Mental Health	Health Distress	0.14
	Overall Quality of Life	0.18
	Emotional Well-Being	0.29
	Role Limitations – Emotional	0.24
	Cognitive Function	0.15

The change from baseline for composite scores, subscale scores and the two single items are defined as the post-baseline score minus the baseline score.

9.2.11. Electrocardiograms (ECGs)

Single ECG measurements were scheduled to be taken at Screening, Weeks 2, 4, 12, 24, 36 and 48. If more than one ECG was collected at a visit, the last ECG performed will be used as the evaluable ECG. All ECGs will be listed.

9.3. Assessment Windows

9.3.1. Study Phases

The study consists of three phases: **Pre-Treatment**, **Treatment**, and **Follow-Up**. The Treatment Phase is further divided into a **Titration** Period (lasting up to 5 weeks) and a **Maintenance** Period. All visits in the treatment and follow-up phases should be scheduled relative to the Baseline (Week 0) visit. The treatment phase will last for 48 weeks and follow up phase will last for two weeks. Therefore, the study phase from the start of treatment to the end of the follow up phase will be approximately 50 weeks in duration.

AEs will be phased based on imputed dates and the definition of imputed AE dates is considered in Section 12.1.1.

The **Pre-Treatment phase** is defined as the period of time prior to the first dose of randomised investigational product.

As per the protocol, the first dose of study medication is taken on the same day as the baseline assessments, such that baseline will be Day 1. Note, it is assumed that baseline assessments were completed before the first dose of study medication was taken; therefore, Day 1 for visit based data will be assigned to the pre-treatment phase.

The **Treatment phase** starts on the date of first dose of randomised study medication (defined as Day 1) for non-visit based data and ends three days after the last dose date.

See Section 9.2.6 regarding the definitions associated with the **Titration** Period and **Maintenance** Period.

For visit based data when the date is the same as the Maintenance period start date, it is assumed that such assessments were completed before the Maintenance period dosing began, therefore such data is considered to have occurred during the Titration period with the exception of phasing of the compliance dataset which has the period attributed to the Maintenance period.

All MRI and MSQoL data occurring after Day 1 will be considered to have occurred during the Treatment phase.

The **Follow-Up** phase starts on the fourth day after the last dose date. All visit based assessments in this phase will be assigned a visit description of ‘Follow-up’.

9.3.2. Assessment Windows

The protocol had no defined assessment windows.

Data collected at assessment visits provide information of the status at that point in time (e.g. efficacy measures, vital signs, laboratory parameters, etc.) and may provide biased results if the visit is attended early or late, in which case the subject will have received more or less treatment than scheduled. For this reason, visits within the Treatment Phase’s Maintenance Period will be slotted with similar study drug medication exposure and assessments conducted outside of the windows specified in the RAP will be considered as “Out of Visit slot”.

Assessments (scheduled and unscheduled as well as early withdrawal) will be first phased as defined in Section 9.3.1. Pre-treatment unscheduled assessments will be assigned to either Screening or Baseline (Week 0), according to whichever visit is closest. e.g. unscheduled *visitnum* variable assignments like 10.01, 10.02 etc. will be slotted to Screening (*avisnum* of 10), with *visitnum* variable assignments like 20.01, 20.02 etc. being slotted to Baseline (Week 0), *avisnum* of 20.

Titration Period visits will not be slotted but reported as per the eCRF. However, for Columbia Suicide Severity Rating Scale data, which is all provided as Unscheduled visits and visit numbers, these are considered as occurring at the nearest earlier integer visit number and reported at the corresponding visit. Other assessments with Unscheduled visit data reported during this Titration Period will be reported as “Out of Visit slot” data.

Non-MRI visit based assessments from the Maintenance Period will be visit slotted according to the time intervals shown in Table 11 with days relative to the date of first dose of double blind study medication (Day 1). It should be noted that assessments can only be slotted to visits that are specified in the protocol for that assessment to be conducted, as those are the assessments that will be summarised in data tables and figures. Data will be analysed according to the analysis window to which it is assigned.

MRI data at scheduled assessments will not be slotted. Early withdrawal MRIs will be slotted according to the time intervals shown in Table 12.

MSQoL data will not be slotted and early withdrawal data for this assessment will be identified as Week 48 data. However, if more than 15% of subjects have their MSQoL assessment conducted more than 14 days after the last dose of study medication, additional sensitivity analyses using assessments within 14 days of the last dose of study medication may be conducted (see Section 13).

Scheduled PK data will not be phased or slotted. Unscheduled PK data will be slotted by using the *floor* of the associated visit identifier.

Visit Slotting Intervals

Table 11 Assessment Windows for Maintenance Period assessments (Non-MRI data)

Visit	Protocol target visit day [interval] relative to day 1	Analysis window (Days)
Week 8	56	43-70
Week 12	84	71-98
Week 16	112	99-126
Week 20	140	127-154
Week 24	168	155-182
Week 28	196	183-210
Week 32	224	211-238
Week 36	252	239-266
Week 40	280	267-294
Week 44	308	295-322
Week 48	336	323-343
Follow-Up	350	≤28 days post Week 48 Visit/Early Withdrawal Visit

Table 12 Assessment Windows for Early Withdrawal visit MRIs

Visit	Protocol target visit day [interval] relative to day 1	Analysis window (Days)
Week 6	42	1-63
Week 12	84	64-105
Week 18	126	106-147
Week 24	168	148-189
Week 30	210	190-231
Week 36	252	232-273
Week 42	294	274-315
Week 48	336	≥316

Evaluability

If an Early Withdrawal MRI slots to a window where a scheduled MRI was conducted then the Early Withdrawal data will not be considered to be evaluable.

For data other than MRI, if there is more than one assessment of a given type that is assigned to a given analysis window then the evaluation closest to the target visit day will be used in the data summary tables and analyses, although all values within a window will be included in the data listings. The last pre-treatment value, however, will be used as the baseline assessment unless specified otherwise.

Summaries provided for ‘any time on-treatment’, such as laboratory values of potential clinical concern, will use any data that slots into the treatment phase. A subject will be counted once in each of the categories that they fall into within this treatment phase, therefore a subject may be counted in the table more than once.

For safety data where two or more assessments are slotted to the follow-up visit, then summary tables will use the assessment recorded closest to the target day (14 days after last dose of double-blind medication).

Evaluability for Cogstate assessments is summarised separately under Section 9.2.9.

Efficacy data that slots to the visit of Follow-up, will be set to non-evaluable as it will be an assessment conducted off-study medication for a long period and therefore will not be summarised; all data will however be listed.

9.4. Values of Potential Clinical Importance

9.4.1. Laboratory Data

The following flagging criteria are used for the selected laboratory parameters:

- *Above or below normal range*: These flags denote values that fall outside the normal range. Values above the normal range are flagged 'F1 High' and those below as 'F1 Low'.

- *Values of clinical concern*: These flags denote values that fall outside the clinical concern values. Values above the upper cut-off for clinical concern are flagged 'F3’ High' and those below the lower limit of clinical concern are flagged as 'F3 Low'.

The F3 range for a particular laboratory measurement is calculated in one of two ways:

1. ***Absolute*****:** Pre-specified limits.
2. ***Normal Range Limit***: The upper limit is the normal range upper limit multiplied by a factor. The lower limit is the normal range lower limit multiplied by a factor.

The lab normal ranges, and values of potential clinical concern are given in Section 16.3.

9.4.2. Vital Signs

Table 13 shows the criteria that are used to determine whether a subject's vital signs (blood pressure and pulse) lie outside of a pre-determined normal range and have a change from baseline of potential clinical importance.

Table 13 Definition of Criteria for Blood Pressure and Heart Rate of Potential Clinical Concern (PCC)

Parameter	Safety Summary Criteria for Values of Potential Clinical Concern[1]		
	Reference Range	Increase from Baseline	Decrease from Baseline
Systolic Blood Pressure, mmHg	90-140	≥ 30	≥ 30
Diastolic Blood Pressure, mmHg	50-90	≥ 20	≥ 20
Heart Rate, bpm	50-100	≥ 30	≥ 30
Weight, kg	N/A	≥ 7%	≥ 7%

1. Values identified as of potential clinical concern are BOTH outside of the reference range AND meet a change from baseline criterion. Note: Weight just needs to meet the change from baseline criterion

9.4.3. ECGs

Table 14 shows the criteria that are used to determine whether a subject's ECG measurements are of potential clinical importance.

Table 14 Definition of Criteria for Potential Clinical Importance (PCI) for ECG Measurements

Grade	Absolute QTc Values	QTc increase from baseline
1	≤450	≤30
2	>450 -≤480	>30 - ≤60
3	>480 -≤500	>60
4	>500	

10. STUDY POPULATION

Subjects who were randomised but failed to meet the Intent-to-Treat (ITT) definition will be included in the Randomised Population. For these subjects, all data will be listed under the treatment group they were randomised to receive. Similarly data from subjects who receive incorrect study medication, at any point during the study, will be summarised under their randomised treatment group.

10.1. Disposition of Subjects

Disposition of subjects will be covered by the following summary tables. These tables will be presented for All Subjects and by Stratum (Background Disease Modifying Therapy: Avonex/Copaxone).

- Summary of Subjects by Population (for the Randomised population; Table 6.01)
- Summary of Inclusion/Exclusion Deviations (for the ITT population; Table 6.02)
- Summary of Subject Disposition (for the ITT population; Table 6.03)

Table 6.01 will give details of the number of subjects randomised, and the number included in the ITT, PK and PGx populations. A listing of subjects excluded from the populations, and the reasons for the exclusion, will be presented by treatment group and subject (Listing 6.01). Table 6.02 will detail the number of all inclusion and exclusion criteria violations and will be split by treatment group. Inclusion/exclusion criteria deviations will be listed in Listing 6.02.

The number and percentage of subjects who completed the study and who withdrew prematurely from the study will be presented by treatment group for All Subjects and by Stratum. Reasons for premature withdrawal will be presented in the order they are displayed in the eCRF. In addition, a summary of the number of subjects completing the Titration Period (defined as starting the Maintenance Period) will be included. This table will be presented for the ITT population (Table 6.03). A listing of all subjects that withdrew will be provided (Listing 6.04). A summary table of the number of subjects who completed each protocol-specified visit will be provided (Table 6.04); it will also include each up-titration phone call that was specified in the protocol. All study visits, including telephone calls will be listed with the exception of unscheduled-visits post-randomisation (Listing 6.05).

A summary table of the reasons for Screening Failures recorded in the eCRF will be provided (Table 6.05).

10.2. Protocol Deviations

A summary of all protocol deviations recorded in the eCRF will be produced for the ITT population (Table 6.06). These data will be listed, for all randomised subjects, in Listing 6.06. This listing will identify the study phase in which the deviation occurred.

Subjects for whom the treatment blind was broken (as recorded on the “Blind” page of the eCRF) will be listed in Listing 6.07, and subjects who were randomised to the incorrect strata will be listed in Listing 6.08.

10.3. Demographic and Baseline Characteristics

10.3.1. Demographics

Demographic data will be summarised by treatment group for the ITT Population (Table 6.07). Summary statistics (number of subjects, mean, and standard deviation, median, minimum and maximum) will be presented for Height, Weight, BMI, age (at baseline), and the number and percentage of subjects will be presented for each sex and ethnic origin category. Demographic data will be listed for all randomised subjects (Listing 6.09).

Two tables will summarise race and racial combinations for the ITT population and the data will be listed (Listing 6.10) for all randomised subjects. Table 6.08 will summarise race by highest level race categories and racial combinations. The high-level race categories and designated Asian sub-categories that will be summarised are:

1. African American/ African Heritage
2. American Indian or Alaska Native
3. Asian
   - Central/South Asian Heritage
   - Japanese/East Asian Heritage/South East Asian Heritage
   - Mixed Asian Heritage (only required if data exists)
4. Native Hawaiian or other Pacific Islander
5. White

Combinations will be represented as the concatenation of the high level category terms. A subject will only be represented in a single category. A subject who selects a combination of races will be counted in the combination of terms, not in each of the constituent terms. Therefore the counts will add up to the total number of subjects with a response and the percentages will add up to 100%.

The second of these two race summaries will summarise race by second-level race categories and racial combinations (Table 6.09). The second-level race categories that will be summarised are:

1. African American /African Heritage
2. American Indian or Alaska Native
3. Asian – Central/South Asian Heritage
4. Asian – East Asian Heritage
5. Asian – Japanese Heritage
6. Asian – South East Asian Heritage
7. Asian – Mixed Race
8. Native Hawaiian or other Pacific Islander
9. White – Arabic/North African Heritage
10. White – White/Caucasian/European Heritage
11. White – Mixed Race
12. Mixed Race

A subject will only be represented in a single category. A subject who selects a combination of races will be counted as 'Asian – Mixed Race', 'White – Mixed Race' or 'Mixed Race' but not in each of the constituent terms.

10.3.2. Medical History

Medical history data will be summarised. Past Medical conditions will be summarised in Table 6.10 and Current Medical conditions will be summarised in Table 6.11, for the ITT population and listed for all randomised subjects (Listing 6.11). Conditions will be summarised under each category specified in the eCRF, with an "Other Conditions" category for other conditions entered by the Investigators. The number of subjects with a family history of cardiovascular risk-factors will be summarised (Table 6.12) and a listing will be provided (Listing 6.12).

10.3.3. MS Disease History

MS disease history is collected at Screening. A summary table displaying summary statistics (for disease duration, time since onset of symptoms, the number of relapses in the previous 12 months, number of relapses in the previous 24 months, total number of relapses, number of days since the last relapse and frequency counts for total number of relapses, number of relapses in the last 12 months , number of relapses in the last 24 months, the proportion of subjects who had a MRI scan in the last twelve months will be produced (Table 6.13) for the ITT Population. For subjects that had an MRI in the last 12 months, frequency counts of the number of MRI scans, number of scans showing active GdE lesions and the number of unique active GdE lesions detected in the MRI scans will also be presented in Table 6.27; these summaries will use the proportion of subjects with an MRI scan in the past 12 months as the denominator. As well as the overall ITT population summary Table 6.13 will show a summary of MS disease history by randomisation stratum. The frequency count of relapses will be categorised as follows: 0, 1, 2, 3-5,>5 relapses. Data will also be listed for all randomised subjects (Listing 6.13), with a separate listing for data from brain MRI scans in the 12 months prior to Screening (Listing 6.14).

Time since first symptoms/disease duration/last steroid use will be determined using the date of the Screening visit and the date of first symptoms/date of diagnosis/date of last steroid use as recorded on the eCRF. If the date of diagnosis or the date of first symptoms is a partial date then a '01' will be used for the day and 'Jan' will be used for the month. If the year of first symptoms/diagnosis/steroid use is missing the date will not be imputed.

10.3.4. Smoking History

History of tobacco use will be summarised by treatment group (Table 6.14) for the ITT population.

A listing of tobacco use will also be provided (Listing 6.16) for all randomised subjects.

10.4. Concomitant Medications

All prior/concomitant medications taken during the study will be recorded on the eCRF. Medications will be coded using the GSK Drug coding dictionary, and will be reported by Anatomical Therapeutic Chemical (ATC) class and medication.

Prior MS disease modifying medications collected on the separate eCRF page at Screening will be summarised in Table 6.15 and listed in Listing 6.17.

Summaries of prior medications in the pre-treatment phase (Table 6.16), concomitant medications during the Titration Period (Table 6.17), concomitant medications during Maintenance Period (Table 6.18) and an overall summary of concomitant medications during the treatment phase will be produced by ATC level 1 and ingredient combinations (Table 6.19). These Treatment Phase displays will exclude the protocol-specified MS Disease Modifying Background Therapy (Avonex/Copaxone). A summary of medications initiated post-treatment will be provided (Table 6.20).

All prior/concomitant medications will be listed (Listing 6.18). A listing detailing the relationships between GSK-Drug ATC classification level 1 (Body System), ingredient and the verbatim text will be provided for all verbatim medications administered (Listing 6.19).

Prohibited Medications

For prohibited medications please refer to the Protocol Section 5.10.2. A summary table (Table 6.21) and listing (Listing 6.20) of prohibited medications taken during the Treatment Phase will be provided. Prohibited medications will be identified via a blinded clinical review.

Medication Classification

Prior and concomitant medications will be defined using the start and stop dates, and ongoing fields recorded in the CRFs relative to the first and last dose dates of randomised study medication. The definitions for prior, concomitant and core follow-up medication periods are shown schematically in the diagram below. Table 15 illustrates how a medication is classified as Prior, Concomitant (during Titration Period), Concomitant (during Maintenance Period) or Post-treatment. A medication is considered to be concomitant Treatment Phase if is assigned to either the concomitant Titration or concomitant Maintenance Periods (i.e. Defined as "Yes" in one of the two corresponding columns in Table 15).

Table 15 Example Medications

	Pre-treatment	Titration Treatment Start Date	On-treatment (Titration Period)	Maintenance Treatment Start Date	On-Treatment (Maintenance Period)	Treatment Stop Date	Post-Treatment Follow-Up	Prior	Concomitant (Titration)	Concomitant (Maintenance)	Post
(a)	x——x							Y	N	N	N
(b)	x——		——x					Y	Y	N	N
(c)	x——		——		——x			Y	Y	Y	N
(d)	x——		——		——		——x	Y	Y	Y	Y
(e)			x——x					N	Y	N	N
(f)			x——		——x			N	Y	Y	N
(g)			x——		——		——x	N	Y	Y	Y
(h)					x——x			N	N	Y	N
(i)					x——		——x	N	N	Y	Y
(j)							x——x	N	N	N	Y
(k)	?——x							Y	N	N	N
(l)	?——		——x					Y*	Y	N	N
(m)	?——		——		——x			Y*	Y*	Y	N
(n)	?——		——		——		——x	Y*	Y*	Y*	Y
(o)	x——		——		——		——?	Y	Y**	Y**	Y**
(p)			x——		——		——?	N	Y	Y**	Y**
(q)					x——		——?	N	N	Y	Y**
(r)							x——?	N	N	N	Y
(s)	?——		——		——		——?	Y***	Y***	Y***	Y***

x = start/stop date of prior/concomitant medication
Y=Yes; N=No
? = missing start/stop date of prior/concomitant medication
* If a medication is stopped on-treatment (either Titration or Maintenance) or post-treatment and no start date is recorded it will be assumed that the medication was ongoing from the pre-treatment phase
** If a medication is started pre-treatment or on-treatment or during the post-treatment Follow-Up and no stop date is recorded then usage will be assumed to be ongoing for the remainder of the study
*** If a medication has no start or stop date it will be assumed that the medication was ongoing from the pre-treatment phase to the post-treatment Follow-Up phase

If a medication is started or stopped on the same day of taking the first or the last dose of randomised study medication plus 3 days then this will be considered a concomitant medication. It should be noted that the same medication can be counted in more than one phase.

If a medication is started or stopped on the same day of taking the first or, up to and including, the day of the last dose of randomised study medication then this will be

considered a concomitant medication. It should be noted that the same medication can be counted in more than one phase/period.

Partial Dates

Note that if either of the start dates or stop dates of double-blind study medication are missing, the worst, or most conservative, case should be considered when slotting medications (i.e. the medications should slot into all possible phases).

If a partial date is recorded in the CRF, the following convention will be used to assign the medication:

- if the partial date is a start date, a '01' will be used for the day and 'Jan' will be used for the month
- if the partial date is a stop date, a '28/29/30/31' will be used for the day (dependent on the month and year) and 'Dec' will be used for the month.

The recorded partial date will be displayed in listings.

Counting Rules

In the tables, the following rules apply when counting prior / concomitant / follow-up medications:

1. If a subject receives the same prior / concomitant / follow-up medication (i.e. same ATC level 1 class and ingredient) more than once, they are only counted once under the count for that ingredient.
2. If a subject receives more than one prior / concomitant / follow-up medication in a particular ATC class, they will only be included once in the count for the ATC class, but will appear in the count for each appropriate ingredient within the ATC class (unless it is the same ingredient, see 1. above).

Therefore, the sum of the numbers of subjects with each ingredient within an ATC class may exceed the number of subjects with medications within that ATC class. Similarly, the sum of the ATC class totals may exceed the total number of subjects with at least one medication.

Multi-ingredient Medications

Multi-ingredient medications will be summarised according to their combination ATC classification rather than the ATC classifications of the ingredients.

Definition of Onset

The following convention will be used when calculating study day:

The start of medication relative to study medication is the number of days between the start of the medication and the start of study medication, i.e.

1) If the date of interest is on or after the first dose date of study medication:

Start of medication relative to start of study medication = start date of medication – first dose date of study medication + 1

2) If the date of interest is before the first dose date of IP:

Start of medication relative to start of study medication = start date of medication – first dose date of study medication

10.5. Exposure and Treatment Compliance

A listing of randomised treatment by centre will be presented in Listing 6.21.

10.5.1. Exposure

A summary of exposure to study medication will be presented (Table 6.22) for the ITT population. This table will display summary statistics for the duration of exposure and cumulative exposure by treatment group. The summary statistics used will be the number of subjects, mean, standard deviation, median, minimum and maximum. The duration of exposure will also be summarised by presenting the number (and percentage) of subjects in each treatment group with duration of exposure in the following categories: 0 days, 1-28 days, 29-56 days, 57-84 days, 85-168 days, 169-252 days, 253-343 days and 344+ days. All exposure data will also be listed (Listing 8.22) for all randomised subjects.

The expected duration is 336 days (7 days x 48 weeks). The duration of exposure in days will be based on the formula:

Duration of exposure in days = date of last study medication dose –date of first study medication dose + 1

The duration of exposure will not be adjusted for breaks in dosing of study medication.

The cumulative exposure to GSK239512 will be based on the formula:

$$Totaldose = \sum_{i=1}^{4} d_i * t_i$$ where i = dose level; t_i = duration of exposure at dose level.

For a subject on GSK239512 that progresses through the study as planned, the expected cumulative exposure would therefore be 25,690µg:

[10µg * 7 (days)] + [20µg * 7 (days)] + [40µg * 7 (days)] + [80µg * 315 (days [45 weeks * 7 days)] = 25,690µg

The duration of exposure will also be plotted by treatment group (Figure 6.01) for the ITT population.

10.5.2. Treatment Compliance

Each subject was planned to be dispensed one bottle of IP at each visit during the Titration and Maintenance Periods of the study. Each bottle dispensed during the Titration Period contained 10 tablets and 4 or 5 bottles were dispensed per subject during the Titration Phase depending on whether an additional week of titration from Week 4 to 5 was required. Bottles dispensed during the Maintenance Period contained 33 tablets. All bottles were labelled with a unique container number. The subject was instructed to take one tablet once daily, i.e., a total of 1 tablet per day. Subjects returned their medication for a compliance check at each study visit and the number of tablets returned in each bottle was recorded in the eCRF.

Compliance for the Treatment Phase, the Titration Period and the Maintenance Period will be calculated separately using the following formula:

$$\left(\frac{\{\text{total no. of tablets dispensed - total no. of tablets returned}\}}{\text{no. of days scheduled to be on treatment}}\right)*100\%$$

where no. of days scheduled to be on treatment = (date of last dose – date of first dose) + 1 day. Note: this is calculated separately for each Phase/Period.

Subjects who have an unknown number of tablets taken at a specific visit due to missing data in the CRF, will be assumed to have taken 0 tablets between visits in the calculation of compliance.

A listing will be provided of all investigational product dispensed and the number of tablets returned at each visit (Listing 6.24) for all randomised subjects.

Subjects are considered to be overall compliant if the calculation for compliance is ≥80% and ≤120% and they have no evidence of any interruption of study medication for 4 or more consecutive days.

Study medication compliance (Treatment Phase, Titration Period and Maintenance Period) and Overall Compliance will be listed (Listing 6.24) for the randomised population and summarised in Table 6.23, Table 6.25 and Table 6.26 for the ITT Population respectively. The summary of compliance will also show the frequency counts for the number of subjects with compliance <80%, ≥80% to ≤120% and >120%.

A subject who received incorrect study medication (incorrect dose level or incorrect study medication) will be listed in Listing 6.25. The information for this second listing will be provided by the RAMOS co-ordinator following DBF which should be interpreted alongside the listing of Protocol Deviations (Listing 6.06) to fully interpret the data.

10.5.3. Dose Titration

A frequency table will be provided to show the number of subjects at each dose level at the start of each week of the titration period. In addition, the number of subjects at each dose level at each visit during the Maintenance period, and the start and the end of the

Maintenance period, and the maximum dose level attained, and maintained for at least 7 days in the Maintenance period, will also be presented (Table 6.24).

11. EFFICACY ANALYSES

Inferences will be made using the intention-to-treat (ITT) population, no other population is defined for efficacy analyses.

11.1. Primary Efficacy Analyses

11.1.1. Summary Displays

A summary table that shows the number of lesions with MTR MRI assessments for subjects at each MRI assessment will be provided. The lesion categories will be 0 lesions, 1 lesion, 2, lesions, 3 lesions, 4 lesions, 5 lesions, 6-10 lesions, and >10 lesions (Table 7.01 & Table 7.02). Lesions occur at different times during the study so summary statistics will be provided by the Relative MRI scan for each lesion rather than study visit to enable similar data to be grouped appropriately. The number of subjects with lesion measurements, the number of lesions, and summary statistics of MTR Value (mean, standard deviation, median, minimum and maximum) will be provided at each MRI scan for GdE lesions and delta-MTR lesions separately (Table 7.03 & Table 7.04). Summary statistics for the change from Reference MRI at each relative MRI for GdE lesions and delta MTR lesions will be presented in Table 7.05 & Table 7.06. It should be noted that the number of subjects and the number of lesions will vary depending on when the lesion occurred. All these summary tables will be presented for all subjects and by Background MS Disease Modifying Medication. The raw mean and 90% CI for MTR values and change from reference MRI in MTR Values and 90% CI will also be plotted (Figure 7.01- Figure 7.04).

Summary statistics for MTR Values and change from Reference MRI MTR Values will also be provided at each MRI scan for GdE lesions and delta-MTR lesions separately at the subject level where all lesions for a subject are averaged as per Section 9.2.7 (Table 7.07- Table 7.10).

Profile plots of lesion MTR values will also be provided for GdE Lesions (Figure 7.05) and delta-MTR Lesions (Figure 7.57). These will be plots of 1 page per subject and will use the study MRI visits such that the timing of lesion occurrence for each subject can be observed. Gadolinium-enhancing lesions will be presented using solid red lines and delta-MTR lesions will be presented using dotted blue lines. The plots will be ordered by treatment group, Background MS Disease Modifying Medication, centre and subject number.

All MTR listings will be sorted by lesion type (gadolinium-enhancing lesion or delta MTR lesion), treatment group, centre, subject, lesion ID (lesions at earliest MRI scans will be listed first). All MTR data received from NeuroRx will be listed (Listing 7.01), this listing will include defining the Reference MRI scan and the change from Reference Scan in MTR Value for each MRI. A separate listing (Listing 7.02) will be provided that

for each subject lesion summarises the average pre-lesion MRI MTR Values and post-Lesion MRI MTR Values for each sensitivity analysis. Listing 7.03 will then repeat Listing 7.02 but using subject level data (averaging across all lesions for a subject).

A listing of subjects that had no GdEs or no delta-MTR lesions such that they did not contribute to either co-primary endpoint will be provided (Listing 7.04).

11.1.2. Statistical Analysis

All analyses described in this section may be conducted for both co-primary endpoints and the corresponding data displays are detailed in Table 16 for ANCOVA analyses and Table 17 for MMRM analyses. However, whilst each potential sensitivity analysis is defined, an iterative approach will be taken to look at different analyses based on the data such that not all specified analyses may necessarily be conducted.

An analysis of covariance will be conducted to assess the change in MTR Recovery from pre-lesion to post-lesion MTR at the lesion level. The ANCOVA model will include fixed categorical terms for treatment group, pre-lesion average MTR Value level and Background MS Disease Modifying Therapy. To account for multiple lesions occurring per subject a “REPEATED=SUBJECT” term will be specified to account for potential correlation in lesion recovery within a subject, a compound symmetry covariance structure will be specified. However, if on review of data it appears that lesions within a subject are heterogeneous, or the restriction results in this analysis or the Bayesian analysis not being able to converge, then this restriction may be removed. The impact of the removal of this restriction will be investigated. If a subject has no lesions then that subject will not contribute to the analysis.

An analysis of covariance will be conducted to assess the change in MTR Recovery from pre-lesion to post-lesion MTR at the subject level. The ANCOVA model will include fixed categorical terms for treatment group, pre-lesion average MTR Value level for all lesions for the subject and Background MS Disease Modifying Therapy. If a subject has no lesions then that subject will not contribute to the analysis.

In order to aid clinical interpretation of the analyses, the results from the ANCOVA statistical modelling will also be calculated as standardised Effect Sizes; with associated 90% confidence interval as per the definition in Section 7.

The Bayesian analysis will be based on a non-informative prior, using the prior statement within the mixed procedure in SAS (with the default Jeffreys prior option). A table showing the posterior probabilities, obtained from 500,000 simulations and 90% credible intervals for the standardised Effect Size for the change in MTR Value post-lesion relative to pre-lesion at End of Study being greater than 0, 0.25 and 0.5 (see Table 16) will be produced along with a figure of the posterior distribution for the ITT population.

Table 16 Summary of ANCOVA Analyses for Co-Primary Endpoints

Sensitivity Analysis*	Gadolinium Enhancing Lesions		Delta-MTR Lesions	
	Lesion Level	Subject Level	Lesion Level	Subject Level
1 - ANCOVA	Table 7.07	Table 7.23	Table 7.15	Table 7.31
- Bayesian^	Table 7.08	Table 7.24	Table 7.16	Table 7.32
	Figure 7.06	Figure 7.10	Figure 7.08	Figure 7.12
1# - ANCOVA	Table 7.09	Table 7.25	Table 7.17	Table 7.33
- Bayesian^	Table 7.10	Table 7.26	Table 7.18	Table 7.34
	Figure 7.07	Figure 7.11	Figure 7.09	Figure 7.13
2	Table 7.11	Table 7.27	Table 7.19	Table 7.35
3	Table 7.12	Table 7.28	Table 7.20	Table 7.36
4	Table 7.13	Table 7.29	Table 7.21	Table 7.37
5	Table 7.14	Table 7.30	Table 7.22	Table 7.38

* Refer to RAP Section 9.2.9 for full definition
\# Including Lesions with at least 2 MRI MTR Assessments Pre- and Post-Lesion only
^ Denotes sensitivity analyses where Bayesian analyses will also be conducted.
Note: Analyses defined as Numbers 1, 1# and 3 will be conducted initially only.

The co-primary endpoints will be analysed using a mixed model for repeated measures (MMRM). However a slightly nonstandard approach is initially proposed. First the post-lesion MTR Value change from average pre-lesion MTR Value for each lesion is modelled separately across visits within each subject. This means there are three hierarchic levels, subject, lesion within subject, and visit within lesion-by-subject. If this hierarchic approach means that the MMRM or Bayesian analyses are unable to run, and it is a reasonable assumption to consider lesions within a subject to be independent (i.e. heterogeneity in lesions is observed) this restriction will be removed such that the levels within the model will be lesion and visit within lesion. An explanation for the need to update the model will be investigated within the data structure.

At the lesion within subject level, the date of identification of the lesion is seen as the focus or origin, and all visits are seen relative to that visit, described as the *Relative MRI*. The fixed effects part of the model is defined as treatment group, average pre-lesion MTR value, Relative MRI, lesion size, Background Disease Modifying Therapy; interactions between Relative MRI and treatment group, between average pre-lesion MTR value and Relative MRI, between Background Disease Modifying Therapy and Relative MRI and between lesion size and Relative MRI. The covariance structure at this

level is modelled using a simple form as we expect the variability to be constant across time and the autocorrelation to be non-complex. As such, we introduce an AR(1) covariance along with a simple random effect at the subject-by-lesion level. One reason for this simple structure is that it is consistent with the fixed effects part of the model focusing on the Relative MRI. At the subject level we introduce an additional random subject effect. In the situation where lesions within a subject are treated as independent the model will be defined as above but the covariance structure at the lesion level will be specified as unstructured.

The model will be used to estimate treatment effects of GSK239512 relative to placebo. The adjusted mean, standard error, adjusted treatment difference; and associated 90% confidence interval will be displayed. The least squares mean MTR Value at each Relative MRI will be plotted by treatment group. Relative MRI will be presented on the x-axis and the adjusted mean MTR Value will be presented on the y-axis. The mean at each Relative MRI will be presented with an associated nominal 90% confidence interval. If the term for Background Disease Modifying Therapy is statistically significant for an analysis, post-hoc analyses looking at each stratum separately may be conducted.

In order to aid clinical interpretation of the analyses, the results from the Mixed Model Repeated Measure (MMRM) statistical modelling will also be calculated as standardised Effect Sizes; with associated 90% confidence interval. The effect size will be calculated as per the definition in Section 7.

The Bayesian analysis will be based on a non-informative prior, using the prior statement within the MCMC procedure using the inverse Wishart prior option in SAS v9.3 on either a Windows or Linux platform. A table showing the posterior probabilities, obtained from 500,000 simulations and 90% credible intervals for the standardised Effect Size for the change in MTR Value post-lesion relative to pre-lesion at End of Study being greater than 0, 0.25 and 0 will be produced along with a figure of the posterior distribution for the ITT population.

Table 17 Summary of MMRM Analyses to be Performed

Sensitivity Analysis*	Gadolinium Enhancing Lesions		Delta-MTR Lesions	
	Lesion Level	Subject Level	Lesion Level	Subject Level
1⁻ MMRM (incl. Adj Mean plots)	Table 7.39	Table 7.55	Table 7.47	Table 7.63
	Figure 7.14	Figure 7.22	Figure 7.18	Figure 7.26
- Bayesian^	Table 7.40	Table 7.56	Table 7.48	Table 7.64
	Figure 7.15	Figure 7.23	Figure 7.19	Figure 7.27
1[#] - MMRM (incl. Adj Mean plots)	Table 7.41	Table 7.57	Table 7.49	Table 7.65
	Figure 7.16	Figure 7.24	Figure 7.20	Figure 7.28
- Bayesian^	Table 7.42	Table 7.58	Table 7.50	Table 7.66
	Figure 7.17	Figure 7.25	Figure 7.21	Figure 7.29

Sensitivity Analysis*	Gadolinium Enhancing Lesions		Delta-MTR Lesions	
	Lesion Level	Subject Level	Lesion Level	Subject Level
2	Table 7.43	Table 7.59	Table 7.51	Table 7.67
3	Table 7.44	Table 7.60	Table 7.52	Table 7.68
4	Table 7.45	Table 7.61	Table 7.53	Table 7.69
5	Table 7.46	Table 7.62	Table 7.54	Table 7.70

* Refer to RAP Section 9.2.9 for full definition
\# Including Lesions with at least 2 MRI MTR Assessments Pre- and Post-Lesion only
^ Denotes sensitivity analyses where Bayesian analyses will also be conducted. Figures will be plots of posterior probabilities.
Note: Analyses defined as Numbers 1, 1# and 3 will be conducted initially only.

11.2. Secondary MRI Efficacy Analyses

A listing of comments from NeuroRx provided with the MRI data will be provided (Listing 6.26).

11.2.1. T2 Lesion MTR

T2 Lesion MTR will be summarised using summary statistics (n, mean, standard deviation, median, minimum and maximum) at each visit (Table 7.147) and for change from Screening at each visit (Table 7.148). A plot of raw mean and 90% CI will also be provided (Figure 7.54). All T2 Lesion MTR data will be listed (Listing 7.16)

Change from Screening in T2 Lesion MTR will be analysed using a mixed model for repeated measures (MMRM) with restricted maximum likelihood estimation and an unstructured covariance matrix. The statistical model will adjust for the following covariates: treatment group, visit, Screening T2 Lesion MTR Value, Background MS Disease Modifying Therapy, interactions between visit and treatment group, between visit and Screening T2 Lesion MTR Value and between visit and Background Disease Modifying Therapy will also be included in the model. Subject will be fitted as a random effect.

In the circumstance that there are convergence problems with the MMRM analysis the SCORING=4 option could be used in the MIXED statement, which makes SAS use Fisher scoring for the first 4 iterations. If the convergence problem cannot be resolved, the unstructured covariance matrix will be replaced by ANTE(1) covariance structure in combination with a random subject effect.

The model will be used to estimate treatment effects of GSK239512 relative to placebo. The adjusted mean, standard error, adjusted treatment difference; and associated 90% confidence interval will be displayed for the ITT population (Table 7.149). In order to aid clinical interpretation of the analyses, the results from the Mixed Model Repeated Measure (MMRM) statistical modelling will also be calculated as standardised Effect Sizes; with associated 90% confidence interval. The effect size will be calculated as per the definition in Section 7.

The least squares mean for the change from Screening in T2 Lesion MTR will be plotted by treatment group over the treatment period (Figure 7.55). Week of study will be presented on the x-axis and the adjusted mean change in T2 Lesion MTR on the y-axis. The mean at each assessment will be presented with an associated nominal 90% confidence interval.

The normality assumptions will be assessed by inspection of the following plots:

- Histogram of marginal studentised residuals derived from the MMRM model
- Normal probability plot with simulation envelope

The Bayesian analysis, which will only be performed if the MMRM analyses demonstrate treatment differences between GSK239512 and placebo will be based on a non-informative prior, using the prior statement within the MCMC procedure using the inverse Wishart prior option in SAS v9.3 on either a Windows or Linux platform. A table showing the posterior probabilities, obtained from 500,000 simulations and 90% credible intervals for the standardised Effect Size for the change from Screening in T2 Lesion MTR at End of Study being greater than 0, 0.25 and 0.5 will be produced (Table 7.150) along with a figure of the posterior distribution for the ITT population (Figure 7.56).

11.2.2. MRI Lesion Count Variables

11.2.2.1. Summary Displays

The actual number and cumulative number of new lesions will be listed for the Randomised population, and summarised using summary statistics (n, mean, standard deviation, median, minimum and maximum) and frequency counts (0 lesions, 1 lesion, 2 lesions, 3 lesions, 4-10 lesions, >10 lesions) for each MRI parameter at each visit for the ITT population using the AES dataset (summary statistics only) as defined in Table 18.

Table 18 Summary of Data Displays for Summarising MRI Lesion Count Endpoints

		New GdE lesions	New enlarging T2 lesions	Cumulative Unique Active Lesions	New Unenhancing T1 Lesions	New GdE Evolving to Black Holes
Actual Lesion Count	Summ. Stats (OC)	Table 7.71	Table 7.75	Table 7.79	Table 7.83	Table 7.87
	Freq. Counts (OC)	Table 7.72	Table 7.76	Table 7.80	Table 7.84	Table 7.88
Cum. Lesion Counts	Summ. Stats (AES)	Table 7.73	Table 7.77	Table 7.81	Table 7.85	Table 7.89

		New GdE lesions	New enlarging T2 lesions	Cumulative Unique Active Lesions	New Unenhancing T1 Lesions	New GdE Evolving to Black Holes
	Freq. Counts (AES)	Table 7.74	Table 7.78	Table 7.82	Table 7.86	Table 7.90
	Mean Profile Plot (AES)	Figure 7.30	Figure 7.31	Figure 7.32	Figure 7.33	Figure 7.34
Actual & Cum. Lesion Counts	Listing	Listing 7.05	Listing 7.06	Listing 7.07	Listing 7.08	Listing 7.09

11.2.2.2. Statistical Analysis

The cumulative number of new lesions for each MRI endpoint at Week 48 will be analysed using the AES dataset and the ITT population. These analyses will fit treatment group and Background Disease Modifying Therapy and baseline count of gadolinium-enhancing lesions as categorical variables using a generalized linear model (GLM) assuming an underlying negative binomial distribution with a log-link function, the log of the number of MRI scans will be included as an offset variable. The rate ratio and associated 90% confidence interval will be presented as per Table 19.

The proposed analysis using the negative binomial model assumes that missing data is missing at random (MAR). To examine the sensitivity of the results of the analysis to departures from this assumption, further sensitivity analyses are defined below, and, dependent on the observed data, may be explored using multiple imputation methods based on pattern mixture models [Carpenter, 2013] using the OC dataset.

Using an underlying negative binomial model post withdrawal MRI lesion counts will be imputed conditional upon the subjects own observed number of events prior to withdrawal. This approach allows alternative assumptions about the missing data to be investigated by modifying the post-withdrawal model.

This approach involves firstly fitting the primary analysis negative binomial generalised linear model to the data, and sampling from the posterior distribution (likelihood function multiplied by a non-informative prior) of the estimated parameters (i.e. the betas) associated with the independent variables.

The number of lesions that would have been seen on missed MRI scans based on various assumptions is then estimated for subjects who withdraw early. This number is combined with the observed lesions on available MRI scans and the data is analysed as specified at

the start of this Section. This analysis is repeated multiple times and the results combined using Rubin's formulae [Barnard, 1999].

The assumptions used to impute the missing part of the data for subjects who withdraw early will be as follows:

a) Unconditional Reference. The basis of this approach is that withdrawal from the GSK239512 treatment group represents a new period for the subject and the previous MRI results are not used in the imputation model for lesions post-withdrawal. Instead missing MRI scan lesion results for GSK239512 are imputed using the overall mean number of lesions per MRI scan for the placebo treatment group, conditional only on baseline covariates. Missing data in the placebo treatment group are imputed under randomised-arm MAR assumptions.

b) Jump to Reference. Missing counts will be imputed conditional upon the subjects own observed number of lesions per MRI scan prior to withdrawal. The impact of sampling from this conditional distribution is that if their lesion rate prior to withdrawal is worse than would be expected (positive residual) on GSK239512, their imputed event rate after withdrawal will be worse than the expected lesion rate on placebo. Missing data in the placebo treatment group are imputed under randomised-arm MAR.

c) Copy Reference. The whole distribution even prior to withdrawal is assumed to be the same as the placebo group. This mimics the case where those withdrawing are in effect non-responders. This has a less extreme impact than the Jump to Reference approach. If a subject in the GSK239512 treatment group is higher than the placebo mean then this positive residual will feed through into subsequent observations, to a degree determined by the correlation in the placebo treatment group. Missing data in the placebo treatment group are again imputed under randomised-arm MAR.

Table 19 Summary of Data Displays for Statistical Analyses of MRI Lesion Count Endpoints

	New GdE lesions	New enlarging T2 lesions	Cumulative Unique Active Lesions	New Unenhancing T1 Lesions	New GdE Evolving to Black Holes
GLM Analysis (AES)	Table 7.91	Table 7.93	Table 7.95	Table 7.97	Table 7.99
MI Analysis (OC)	Table 7.92	Table 7.94	Table 7.96	Table 7.98	Table 7.100

11.2.3. MRI Volume Measures

11.2.3.1. Summary Displays

Summary statistics (n, mean, standard deviation, median, minimum and maximum) will be presented for the actual volume at each scheduled MRI assessment and change from baseline (Screening MRI) at each scheduled MRI assessment as defined in Table 20 for the ITT population using the OC dataset and the IMV dataset. Listings of each MRI volume measure for the Randomised population are also defined in Table 20.

Table 20 Summary of Data Displays for Summarising MRI Volume Endpoints

		Norm. Brain Volume (Atrophy)	White Matter Volume	Grey Matter Volume
Actual Volumes	Summary Statistics (OC)	Table 7.101	Table 7.103	Table 7.105
Change from Baseline	Summary Statistics (OC)	Table 7.102	Table 7.104	Table 7.106
	Mean Profile Plot (OC)	Not to be created	Figure 7.35	Figure 7.36
Actual & Changes	Listing	Listing 7.10	Listing 7.11	Listing 7.12

11.2.3.2. Statistical Analysis

The change in whole brain atrophy relative to Screening at Week 48 will be analysed using the OC dataset and the ITT population. The analyses will fit treatment group and Background Disease Modifying Therapy as categorical variables and baseline normalised brain volume at Screening as covariates using a generalized linear model (GLM). The pairwise comparison, and associated 90% confidence interval will be presented (Table 7.107).

The change in White Matter and Grey relative to Screening at Week 48 will be analysed using the OC dataset and the ITT population. The analyses will fit treatment group and Background Disease Modifying Therapy as categorical variables and appropriate baseline brain volume at Screening as covariates using a generalized linear model (GLM). The pairwise comparisons, and associated 90% confidence intervals will be presented (Table 7.108 and Table 7.109).

In addition, the change from baseline for each White and Matter volume endpoint will be analysed using a mixed model for repeated measures (MMRM) with restricted maximum likelihood estimation and an unstructured covariance matrix and for the OC dataset. The statistical model will adjust for the following covariates: treatment group, visit, Baseline volume, Background Disease Modifying Therapy, interactions between visit and treatment group, between visit and Baseline volume and between Background Disease Modifying Therapy and visit will also be included in the model. Subject will be fitted as a

random effect. In the circumstance that there are convergence problems with the MMRM analysis the SCORING=4 option could be used in the MIXED statement, which makes SAS use Fisher scoring for the first 4 iterations. If the convergence problem cannot be resolved, the unstructured covariance matrix will be replaced by ANTE(1) covariance structure in combination with a random subject effect.

The model will be used to estimate treatment effects of GSK239512 relative to placebo at each MRI visit. The adjusted mean, standard error, adjusted treatment difference; and associated 90% confidence interval will be displayed (Table 7.110 & Table 7.111). The least squares mean for the change from baseline will be plotted by treatment group over the treatment period (Weeks 0-48) for all subjects in the ITT population (Figure 7.37-Figure 7.38). Week of study will be presented on the x-axis and the adjusted cumulative number of new lesions will be presented on the y-axis. The mean at each assessment will be presented with an associated nominal 90% confidence interval. If the term for Background Disease Modifying Therapy is statistically significant for an analysis, post-hoc analyses looking at each stratum separately may be conducted.

11.3. Other Efficacy Analyses

11.3.1. CogState Battery

11.3.1.1. Summary Displays

The individual Cogstate tasks data will be listed in Listing 7.13, a "*" shall be used to denote the primary measure for each CogState 'task' and a "^" will be used to denote tasks that were completion or integrity failures. The CogState Battery standardised task scores will be listed by treatment group, visit and subject for the all randomised population (Listing 7.14). This listing will include the comparison to a healthy population for the Baseline assessment. The CogState Battery Total score and composite scores will be listed by treatment group, visit and subject for the all randomised population (Listing 7.15).

A summary table of each CogState Battery task at baseline relative to the age-matched normative scores will be provided to aid interpretation of CogState Battery data by assessing deficits at Baseline (Table 7.112). This table will include summary statistics and the number of subjects ≥2 SDs,<2 and ≥1 SD, <1 ≥0.5 SDs and <0.5 SDs above and below the normative scores. This display will also include a "Total" column for the full study population.

A frequency count of assessments meeting either completion or integrity failure criteria will be summarised for each CogState Battery task by visit (Table 7.113).

The actual value at all scheduled visits and change from baseline value at Weeks 12, 24 and 48 for the primary response variable (raw and standardised) for each Task in the CogState Battery (see Section 9.2.9) will be summarised by treatment group at Weeks 12, 24 and 48 for the ITT Population for the OC dataset (Table 7.114 & Table 7.115). The actual value at all scheduled visits and change from baseline value at Weeks 12, 24 and

48 in the CogState Battery Total score and composite scores will be summarised by treatment group for the ITT Population for the OC dataset (Table 7.116- Table 7.119).

11.3.1.2. Statistical Analysis

Change from Baseline for CogState endpoints will be analysed using a mixed model for repeated measures (MMRM) with restricted maximum likelihood estimation and an unstructured covariance matrix. The statistical model will adjust for the following covariates: treatment group, visit, Baseline score of endpoint, Background MS Disease Modifying Therapy, interactions between visit and treatment group, between visit and Baseline score and between visit and Background Disease Modifying Therapy will also be included in the model. Subject will be fitted as a random effect.

In the circumstance that there are convergence problems with the MMRM analysis the SCORING=4 option could be used in the MIXED statement, which makes SAS use Fisher scoring for the first 4 iterations. If the convergence problem cannot be resolved, the unstructured covariance matrix will be replaced by ANTE(1) covariance structure in combination with a random subject effect.

The model will be used to estimate treatment effects of GSK239512 relative to placebo. The adjusted mean, standard error, adjusted treatment difference; and associated 90% confidence interval will be displayed in for the ITT population as per Table 21. In order to aid clinical interpretation of the analyses, the results from the Mixed Model Repeated Measure (MMRM) statistical modelling will also be calculated as standardised Effect Sizes; with associated 90% confidence interval. The effect size will be calculated as per the definition in Section 7. In addition, this model may also be run for each Background Disease Modifying Therapy stratum using the same model as above (excluding terms for Background Disease Modifying Therapy and the visit by Background Disease Modifying Therapy interaction) for the CogState Battery Total Score and each Composite Domain Score.

The least squares mean for the change from baseline in the value of the CogState Battery Total score and composite scores will be plotted by treatment group over the treatment period (Week 12, Week 24 and Week 48) for all subjects in the ITT population as per Table 21. Week of study will be presented on the x-axis and the adjusted mean value of the CogState Battery Total score or composite score will be presented on the y-axis. The mean at each assessment will be presented with an associated nominal 90% confidence interval.

The normality assumptions will be assessed by inspection of the following plots:

- Histogram of marginal studentised residuals derived from the MMRM model
- Normal probability plot with simulation envelope

The Bayesian analysis will be based on a non-informative prior, using the prior statement within the mixed procedure in SAS (with the default Jeffreys prior option) and will be

conducted for the CogState Battery Total Score and Composite Domain Scores only. A table showing the posterior probabilities, obtained from 500,000 simulations and 90% credible intervals for the standardised Effect Size for the change from baseline in CogState Total Score at End of Study being greater than 0, 0.25 and 0.5 will be produced along with a figure of the posterior distribution for the ITT population.

Note: Analysis of individual CogState tasks, the Bayesian analyses and analyses by randomisation stratum (Background MS Disease Modifying Therapy) will only be conducted if potential effects of clinical interest are observed for GSK239512 from the MMRM analyses of the CogState Battery Total and Composite Scores.

Table 21 Summary of Statistical Analyses of CogState Battery Endpoints

Endpoint	Statistical Analyses		Graphical Displays	
	MMRM	Bayesian	Mean Profile	Posterior Distribution
CogState Battery Total Score	Table 7.120	Table 7.121	Figure 7.39	Figure 7.40
CogState Battery Total Score by Stratum	Table 7.122	N/A	N/A	N/A
CogState Composite Domains				
Executive Function	Table 7.125	Table 7.126	Figure 7.41	Figure 7.42
Executive Function by Stratum	Table 7.127	N/A	N/A	N/A
Memory	Table 7.130	Table 7.131	Figure 7.43	Figure 7.44
Memory by Stratum	Table 7.132	N/A	N/A	N/A
Attention	Table 7.135	Table 7.136	Figure 7.45	Figure 7.46
Attention by Stratum	Table 7.137	N/A	N/A	N/A
Note: Whilst displays by randomisation stratum are defined, the analyses will only be conducted post-SAC following review of the main study results.				
CogState Tasks				
	MMRM Analyses		Adjusted Mean Profile Plot	
Groton Maze Learning Test	Table 7.140		Figure 7.47	
One Back Test	Table 7.141		Figure 7.48	
International Shopping List	Table 7.142		Figure 7.49	

Endpoint	Statistical Analyses	Graphical Displays
Task – Immediate Recall		
International Shopping List Task – Delayed Recall	Table 7.143	Figure 7.50
One Card Learning	Table 7.144	Figure 7.51
Detection	Table 7.145	Figure 7.52
Identification	Table 7.146	Figure 7.53

11.3.1.3. Sensitivity Analysis around Missing Data

It should be noted that analyses described in this Section will only be conducted post-SAC if cognition data shows trends that warrant additional investigations. If the analyses described below are not conducted, an explanation will be provided in the CSR. As a supportive analysis, an analysis of covariance (ANCOVA) may be conducted for Week 48 data as per Table 22. The ANCOVA model will include fixed categorical terms for treatment group, baseline score and Background MS Disease Modifying Therapy.

As an additional sensitivity analysis to assess the impact of missing data, multiple imputations will be drawn from a multivariate normal model for the data (including covariates) with a Markov Chain Monte Carlo (MCMC) approach used to estimate posterior distributions. A non-informative prior will be used. Where a subject has a monotone or non-monotone pattern of missingness, all of their missing observations can be imputed under this approach. Note that in this study it is expected that missingness will be predominantly monotone.

Imputations will be drawn separately for subsets of subjects defined according to their treatment group. Within these subsets, the imputations will be drawn separately for each missingness pattern based upon assumptions for the patterns of means post withdrawal and conditioning on observed covariates. Three methods are proposed:

1. Based on means and variance-covariances from the same treatment group (MAR approach, i.e. comparable to MMRM)

2. Based on placebo group changes in mean from visit to visit and the placebo group variance-covariances (Copy Difference from Control (CDC) approach)

3. Based on means and variance-covariances from the same treatment group. The MAR values will be adjusted to incorporate decline associated with disease progression (Delta approach)

Analyses of Week 48 complete data based on these methods will be conducted for the CogState Battery Total Score and each Composite Domain Score using the same ANCOVA model described above.

Table 22 Summary of Sensitivity Analyses of CogState Battery Composite Scores

	ANCOVA at Week 48	ANCOVA using MI Methods
CogState Battery Total Score	Table 7.123	Table 7.124
Executive Function	Table 7.128	Table 7.129
Memory	Table 7.133	Table 7.134
Attention	Table 7.138	Table 7.139

11.3.2. Exploratory Analyses: Understanding Relationships

Exploratory work is required to understand the relationships between the clinical and MRI endpoints collected in this study to understand the potential clinical impacts of lesion remyelination to aid interpretation of the study and to plan potential future studies with this compound. These investigations are considered totally exploratory and a separate document will specify planned analyses to be conducted after the iSRC have conducted the interim analysis. Based on these initial investigations, additional analyses may be specified to be conducted once the study has completed, these analyses will be defined in another document with analyses defined post-unblinding defined as such in the document.

12. SAFETY ANALYSES

The ITT Population will be used for all safety summaries unless otherwise specified (Note: As per Section 6.1, the ITT Population and the Safety population have the same definition). The listings will use the all randomised population and will be sorted by treatment group, centre and subject unless otherwise stated.

12.1. Adverse Events

Adverse events are captured in the eCRF as verbatim text and are then coded using the MedDRA dictionary such that each AE has a System Organ Class (SOC), Higher Level Group term (HLGT) and a Preferred Term (PT).

For this study, AE reporting will be divided into five phases: 'Pre-treatment', ‘Treatment Phase’, ‘Titration Period’, 'Maintenance Period' and 'Follow-Up' (see Section 9.3). The ‘Titration Period’ and ‘Maintenance Period’ are subsets of the ‘Treatment Phase’. The protocol states that AE reporting should commence after Screening.

12.1.1. General Rules for AEs

1. AEs are to be classified as 'Pre-treatment', 'On-treatment' and 'Follow-Up' using the following classification rules:

   If the AE onset date is before the start date of initiation of investigational product then the AE will be considered a 'Pre-treatment' AE.

   If the AE onset date is any date from the date of initiation of investigational product to 3 days after the date investigational product was permanently discontinued, then it will be considered an 'On-treatment' AE.

   If only the AE end date is known and occurs before the start date of initiation of investigational product, then the AE will be considered a 'Pre-treatment' AE."

   If the AE onset date is more than 3 days after the date investigational product was permanently discontinued then it will be considered a 'Follow-Up' AE.

On-treatment AEs will be classified to be in the Titration Period' or 'Maintenance Period' using the following classification rules:

   If the AE onset date is on or before the date defined in the MSTONE Analysis Dataset as the end of the Titration Period then the AE will be considered a 'Titration Period' AE.

   If the AE onset date is on, or after, the date defined in the MSTONE Analysis Dataset as the start of the Maintenance Period to 3 days after the date investigational product was permanently discontinued, then it will be considered a 'Maintenance Period' AE.

2. The time to occurrence is defined as

If the AE started pre-treatment: AE start date – initiation of investigational product date

If the AE started on or after the first dose of investigational product: AE start date – initiation of investigational product date +1 day

The following rules will be followed for partial AE start dates and AE stop dates to allow relative times of all AEs to be calculated.

AE Start Dates: If a partial AE start date exists and only the day part is missing and the month is the same as the starting month for study medication and the AE end date is either missing or the AE end date is greater than or equal to the start of study medication then the imputed AE start date is set to be the same as the day of first study medication. Otherwise set the AE start date to the 1st of the month. If a partial AE start date exists and only the start year of the AE is known and the year is the same as the start of study medication and the AE end date is either missing or the AE end date is after the start of study treatment then set the AE start date to be the same as first study medication. Otherwise set the date to 01JAN of the known year.

AE Stop Dates: If a partial AE stop date exists then if only the day part is missing, then set it to the end of that month. Otherwise if both the day and month are missing then set it to the 31DEC of the year entered.

3. For the AE listings, the duration of the adverse event will be formatted as follows:

duration = (AE end date – AE start date) +1 = 'X d’

Hence if the dates of onset and resolution for an event are the same the duration will appear as '<1 d'. Where no date of resolution is given, the duration will appear as 'Continuing'.
The time since first dose is defined as:

AE start date – study medication start date + 1 day

The time since last dose is based on the final stop date of study medication and is defined as:

AE start date – study medication stop date + 1 day

Note: the ‘+ 1 day’ is not added in situations where the AE start date is before the study medication start/stop date.

12.1.2. Counting Rules for AEs

In the summary tables, the following rules apply when counting adverse events:

1. If a subject experiences the same AE (i.e. same preferred term) more than once, they are only counted once under the count for preferred term. NB: If a subject reported two identical AEs in the Titration period and in the Maintenance period they will be included in both of these periods but will only be included once in the count of that preferred term in summaries of the Treatment phase.
2. If a subject experiences more than one AE in a particular SOC, they will only be included once in the count for the SOC, but will appear in the count for each appropriate preferred term within the SOC (unless it is the same preferred term, see Point 1. above).

Therefore, the sum of the numbers of subjects with each preferred term event within a SOC may exceed the number of subjects with events within that SOC. Similarly, the sum of the SOC totals may exceed the total number of subjects with at least one event.

3. AEs related to study medication tables include only those AEs with a “Y” response or missing response to the eCRF question asking if the AE is possibly related to study medication.
4. For AE intensity the following rules will be applied in determining the counts of AEs:

An individual subject who experiences two AEs of the SAME preferred term of the SAME intensity will be included ONCE in the appropriate intensity count for the particular AE.

An individual subject who experiences two AEs of the SAME preferred term but DIFFERENT intensities will be included ONCE in the severity count for the higher intensity but not in the count for the lesser intensity.

In the total counts for each intensity classification, an individual subject who experiences two AEs of DIFFERENT preferred terms of the SAME intensity will be included ONCE in the total for the appropriate intensity.

In the total counts for each intensity classification, an individual subject who experiences two AEs of DIFFERENT preferred terms of DIFFERENT intensities will be included ONCE in the total FOR EACH of the appropriate intensities.

12.1.3. AE Collapsing Algorithm

Multiple AEs are collapsed to a single record when the following fields are identical:

1. MedDRA code
2. onset date
3. onset time
4. outcome
5. maximum intensity
6. relationship to drug
7. 'was subject withdrawn?' flag
8. action

The resultant collapsed record will have the latest non-missing stop date. If any of the seven fields above differ, the AEs are not collapsed.

Note: When an AE and SAE are collapsed, the resultant single record will be considered an SAE. All additional fields on the SAE record will be retained.

12.1.4. Adverse Event Reporting

Adverse events will be sorted by SOC and then by preferred term in descending order from the highest total incidence to the lowest total incidence. In addition, the total incidence of adverse events for each treatment group over all SOC and within each SOC will be shown.

Adverse events will be sorted by system organ class and then by preferred term in descending order from the highest total incidence to the lowest total incidence unless otherwise stated.

The following output will be produced:

- Summary of all adverse events split by MS Background Disease Modifying Therapy and by study phase: Pre-Treatment, Treatment, Titration, Maintenance and Follow-Up (Table 8.01)
- Summary of (Investigator assessed) drug-related adverse events split by MS Background Disease Modifying Therapy and by study phase: Pre-Treatment, Treatment, Titration, Maintenance and Follow-Up (Table 8.02)
- Summary of adverse events by maximum intensity and split by MS Background Disease Modifying Therapy and by study phase: Pre-Treatment, Treatment, Titration, Maintenance and Follow-Up (Table 8.03)
- Summary of (Investigator assessed) drug related adverse events by maximum intensity split by MS Background Disease Modifying Therapy and by study phase: Pre-Treatment, Treatment, Titration, Maintenance and Follow-Up (Table 8.04)
- Summary of common adverse events (≥5% incidence in either treatment group) split by MS Background Disease Modifying Therapy and by study phase: Pre-Treatment, Treatment, Titration, Maintenance and Follow-Up (Table 8.05). Note: this display will be sorted by the frequency of Preferred Terms in the GSK239512 group then the Placebo group.
- Summary of (Investigator assessed) drug related common adverse events (≥5% incidence in either treatment group) split by MS Background Disease Modifying Therapy and by study phase: Pre-Treatment, Treatment, Titration, Maintenance and Follow-Up (Table 8.06). Note: this display will be sorted by the frequency of Preferred Terms in the GSK239512 group then the Placebo group.
- Summary of characteristics of adverse events by MS Background Disease Modifying Therapy and by study phase: Pre-Treatment, Treatment, Titration, Maintenance and Follow-Up (Table 8.07). Note: This table will be presented at the Adverse Event level rather than the Subject Level.
- Summary of Adverse Events by MS Background Disease Modifying Therapy and by time of first occurrence (Table 8.08). Time of first occurrence will be categorised as follows: Days 1-7, Days 8-14, Days 15-22, , Days 23-29, Days 30-36, Days 37-56, Days 57-84, Days 85-168, Days 169- 252, >252 Days.

In addition, the following displays will also be provided for the Titration Period only by MS Background Disease Modifying Therapy and will be presented for placebo and each individual dose level as per Section 7.1:

- Summary of all adverse events (Table 8.09)
- Summary of (Investigator assessed) drug-related adverse events (Table 8.10)
- Summary of (Investigator assessed) drug related adverse events by maximum intensity (Table 8.11)

- Summary of common adverse events (≥5% incidence in any dose group; Table 8.12)

The following listings will be produced:

- Listing of all adverse events (Listing 8.01)

This listing will display all collected information relating to every adverse event recorded in the study, by subject number. Basic demographic information is also included.

- Relationship of adverse events system organ classes, higher level group terms, preferred terms and verbatim text (Listing 8.02)

This listing will show a list of all SOCs, HLGTs, preferred terms and verbatim adverse event text collected and reported in this study. Verbatim text will be listed alongside the appropriate preferred term, and similarly the preferred terms will be listed alongside the appropriate HLGT and SOC.

- Listing of subject numbers for individual on treatment adverse events (Listing 8.03)

This listing will display the number of subjects and subject numbers for each preferred term of adverse event, split by SOC and HLGT.

The following figures will be produced:

- Dotplots will be produced showing the relative risks of GSK239512 vs placebo for the most common AEs (defined as ≥5% within either treatment group). The percentage of subjects with each adverse event will be displayed in the left-hand panel and the right-hand panel will show the relative risk, and asymptotic 95% confidence intervals (Figure 8.01).

12.1.5. Adverse Events of Special Interest

Adverse events of special interest (AESI) in this study include:

- Convulsion
- Perceptual abnormalities (e.g., hypnopompic hallucinations)
- Insomnia/sleep disorders
- Abnormal dreams/nightmares
- Mood symptoms (e.g., depressed mood, suicidality, anxiety)
- Feeding and body weight change
- LFT Abnormalities

To aid with the identification of adverse events of special interest, a set of MedDRA dictionary preferred terms and verbatim terms will be pre-specified as search criteria for

each of the event types. These will be stored in a separate document and identified prior to the unblinding of the study.

A summary of all adverse events clinically identified as an AESI excluding LFT abnormalities will be presented sorted by system organ class and preferred term in descending order from the highest total incidence to the lowest total incidence (Table 8.13- Table 8.18). In addition, separate tables will be provided showing the actual number of occurrences of each Adverse Event of Special Interest, and their characteristics (Table 8.19- Table 8.24).

In addition a listing of all AE data for the AESI events will be provided (Listing 8.04). This listing will be sorted by AESI, treatment group, centre and subject. This listing will be a subset of the all Adverse Events listing (Listing 8.01).

The following three additional tables shall also be provided by Study Phase:

- Summary of Adverse Events (AEs) coding to MedDRA HLGT "Sleep disorders and disturbances" (Table 8.25)
- Summary of AEs coding to MedDRA HLGT "Depressed mood disorders and disturbances" (Table 8.26)
- Summary of AEs coding to MedDRA HLGT "Suicidal and self-injurious behaviours NEC" (Table 8.27)

In addition, a listing of all AE data included in these 3 summary tables will be provided (Listing 8.05). This listing will be sorted by HLGT category, treatment group, centre and subject. This listing will be a subset of the all Adverse Events listing (Listing 8.01).

12.1.6. Possible Suicidality Related Adverse Events

Any adverse events that were reported as possible suicidality related adverse events (PSRAEs) will be listed in Listing 8.06- Listing 8.09.

12.2. Deaths and Serious Adverse Events

Any death that occurs in the study is defined as a serious event (SAE), and should be recorded on the SAE pages.

A summary of serious adverse events by treatment group split by fatal/ non-fatal and treatment phase (on treatment and follow-up) will be provided (Table 8.28). Fatal and non-fatal SAEs will be listed in Listing 8.10 & Listing 8.11.

Additional information recorded in the eCRF for SAE’s will be provided in Listing 8.12.

12.3. Device Incidents and Near Incidents

No device is being used in this study.

12.4. Adverse Events Leading to Discontinuation of Investigational Product and/or Withdrawal from the Study and Other Significant Adverse Events

Subjects can withdraw from investigational product due to an adverse event during the treatment phase and enter follow-up.

A summary of adverse events leading to withdrawal of study drug or withdrawal from study, by treatment group split by treatment phase: Treatment, Titration and Maintenance will be provided (Table 8.29).

All AEs leading to withdrawal will be listed (Listing 8.13). This listing will be a subset of the listing of all treatment AEs (Listing 8.01).

12.5. Pregnancies

Female subjects of childbearing potential are being recruited into this study. They are required to have a negative pregnancy test at Screening and baseline, and then at each visit during the treatment phase and at every visit during the Follow-up period. For pregnancies that occur during the study, they are followed up by the GCSP group and case narratives will be provided for the study report so no further information will be provided in this RAP. Child Bearing potential at Screening will be listed in Listing 6.03.

12.6. Clinical Laboratory Evaluations

12.6.1. Haematology and Chemistry

Summary statistics (n, mean, standard deviation, median, minimum and maximum) for absolute values and change from baseline at each scheduled post-baseline assessment and the maximum on-treatment value will be tabulated by treatment group for all laboratory parameters collected at each scheduled assessment (Table 8.30 to Table 8.33). Note: FSH and estradiol will be listed only and not included in any summary tables.

Laboratory values at each assessment will be compared with both the appropriate normal ranges (F1 flag) and potential clinical concern (PCC) ranges (F3 flag). Lab values will be presented for each treatment group using classification by normal ranges as: within range, below the range (i.e. below the lower limit) or above the range (i.e. above the upper limit). Subjects with actual values or changes in lab measurements of potential clinical concern (see Section 9.4.1) will be summarised separately at each scheduled assessment and the maximum on-treatment value for each treatment group (Table 8.34 to Table 8.37). If a reference range, or range of clinical concern, is not provided, the parameter will not be included in the data display.

Shift tables will be provided to show the number and percentage of subjects with transitions from baseline (within normal range, above normal range and below normal range) to each scheduled post-baseline assessment and the maximum on-treatment value (within normal range, above normal range and below normal range) in each treatment

group for haematology data and chemistry data. These tables will use the F1 (normal range) and be repeated using F3 flags, as specified in Section 9.4.1 (Table 8.38 to Table 8.41). Parameters where no F3 flags are specified will be excluded from the data display.

A listing will be produced showing laboratory data from *all* assessments for subjects who have any values of potential clinical concern during the study (Listing 8.15). In addition, a subset listing will also be provided showing just the laboratory values of potential clinical concern (Listing 8.14).

Boxplots of each haematology and chemistry parameter at each scheduled assessment will be provided; each parameter will be presented with values divided by the upper limit of normal. The number of subjects at each visit in each treatment group will be presented (Figure 8.02 and Figure 8.03). To aid interpretation of each graph, any post-baseline on-treatment value >3xULN will be set to be 3xULN and will be annotated with the subject number and in parentheses the actual multiple above the ULN.

A shift plot for each haematology and chemistry parameter will also be presented (Figure 8.04 and Figure 8.05). The x-axis will be the baseline value (standardised by dividing by the ULN) and the y-axis will be the maximum on-treatment value for the laboratory parameter (standardised by dividing by the ULN). A reference line of Y=X will be presented. Each treatment group will be presented using a different colour and each Background Disease Modifying Therapy will be presented using a different symbol. To aid interpretation of each graph, any post-baseline maximum on-treatment value >3xULN will be set to be 3xULN and will be annotated with the subject number and in parentheses the actual multiple above the ULN.

12.6.1.1. Liver Function Tests

A table of graded LFTs for Alanine Amino Transferase (ALT), Aspartate Amino Transferase (AST), Total Bilirubin (BIL), Alkaline Phosphatase (ALP) and Gamma Glutamyl Transferase (GGT) will be provided at each scheduled assessment and maximum on-treatment value (Table 8.42). Graded LFTs will be defined by the following criteria:

- Grade 1: > ULN and ≤3 x ULN
- Grade 2: > 3- ≤5 x ULN
- Grade 3: > 5- ≤20 x ULN
- Grade 4: >20 x ULN

Where ULN=upper limit of normal range

Boxplots will be produced showing the distribution of the liver function test (LFT) parameters (ALT, AST, GGT, alkaline phosphatase and total bilirubin) at each scheduled assessment as part of Figure 8.05 (see Section 12.6.1) and the maximum on-treatment value for each LFT side by side by treatment group (Figure 8.06).

By-subject listings of information on liver events in relation to time of treatment (Listing 8.16) and for calculating the RUCAM score (Listing 8.17) will be produced.

Listings of Liver Imaging and Biopsy data will be produced (Listing 8.18, Listing 8.19).

12.6.2. Urinalysis Results

Urinalysis data will be listed only (Listing 8.20).

12.7. Multiple Sclerosis Safety Assessments

12.7.1. EDSS

Summary displays of EDSS assessments at scheduled assessments will only include EDSS assessments performed when the subject was not affected by an MS relapse. Summary statistics (number of subjects, mean, standard deviation, median, minimum and maximum) and a frequency table of the number of subjects with each EDSS score at each scheduled assessment will be presented by treatment group for the EDSS summary score (Table 8.43). Summary Statistics and a frequency table of the number of subjects in each Functional System Score: Visual Modified Score (Table 8.44), Brainstem Functional System Score (Table 8.45), Pyramidal Function System Score (Table 8.46), Cerebellar Functional System Score (Table 8.47), Sensory Functional System Score (Table 8.48), Bladder/ Bowel Modified Functional System Score (Table 8.49), Cerebral Functional System Score (Table 8.50) and Ambulatory Score (Table 8.51) will be produced by treatment group and visit for the treatment phase.

In addition, a summary of the number of subjects in each treatment group with an improved (by ≥1 point and by ≥2 points), unchanged or worsened (by ≥1 point and by ≥2 points) EDSS Total score at Weeks 12, 24, 36 and 48 compared to their baseline assessment will be provided (Table 8.52). In addition, a summary table of subjects with improved (by ≥1 point and by ≥2 points), unchanged or worsened (by ≥1 point and by ≥2 points) Functional System Scores (FSS) sustained for at least 12 weeks at Week 48 will be provided by FSS (Table 8.53).

A scatter plot of baseline EDSS score versus Week 48 EDSS score will be provided (Figure 8.07). The x-axis will be the Baseline EDSS Total Score and the y-axis will be the Week 48 EDSS Total Score. A reference line of Y=X will be presented. Each treatment group will be presented using a different colour and each Background Disease Modifying Therapy will be presented using a different symbol.

Summary tables and figures of all EDSS data will only include EDSS assessments conducted when the subject was not experiencing the symptoms of a relapse. This will be determined by the response to Question 12 in the eCRF (DMDATA: EDSSSYN.EDSSRELP) such that if the response is “Yes”, the EDSS assessment will be excluded in summary displays; all Baseline EDSS assessments will be included in summary tables and figures. All EDSS assessments will be listed.

The EDSS summary score and the functional system scores will be listed by treatment group (Listing 8.21). Listing 8.22- Listing 8.31 will list the responses to each individual question for each functional system.

12.7.2. MS Relapses

Details of relapses occurring during the study and neurological deficits will be listed by treatment group and subject (Listing 8.32 and Listing 8.33). The EDSS summary score and the Functional System Scores for all EDSS assessments made at the time of relapses and the change in EDSS score from the previous scheduled visit and the following scheduled visit will be listed by treatment group (Listing 8.34). A listing of all steroids administered to subjects during relapses will be provided (Listing 8.35).

The number and percentage (of the ITT population) of subjects experiencing relapses will be summarised by treatment group (Table 8.54) during the treatment phase and during the Follow-up phase separately for all subjects and also by baseline EDSS group (0-2.5, ≥3; Table 8.55). Included in the summary table will be the proportion of relapses that were treated with methylprednisolone or other steroid, the number (percentage) of relapses that required hospitalisation and summary statistics for the time spent in hospital. A summary table of the frequency of the number of subjects with each neurological deficit during the treatment phase will be presented (Table 8.56). For a subject with multiple relapses with the same deficit the most extreme status will be present, i.e. that subject would be included in the “Ongoing” row rather than the “Resolved” row.

For subjects that relapsed during the study, history of previously impacted functional systems during relapses was also collected in the eCRF. This data will be listed (Listing 6.15).

The rate of relapses during the Treatment Phase will be analysed using maximum likelihood based analysis, assuming the Negative Binomial distribution, with time on treatment, measured in days, as an offset variable. The model will include adjustment for Background Disease Modifying Therapy and treatment group, which will be fitted as a categorical variable. The adjusted mean relapse rate over the treatment period, treatment ratio and 90% confidence intervals will be presented (Table 8.57).

The proportion of subjects relapsing during the Treatment Phase in each treatment group will be compared using a logistic regression adjusted for Background Disease Modifying Therapy and treatment group, which will be fitted as a categorical variable. The odds ratio with associated 90% confidence interval will be presented (Table 8.58).

The time to first relapse (based on the earliest symptom start date for a subject’s first relapse) will be compared between treatment groups using a Cox’s Proportional Hazards model adjusting for Background Disease Modifying Therapy and treatment group, which will be fitted as a categorical variable. Tied data will be handled using Efron’s method [Efron, 1977]. The hazard ratio with associated 90% confidence interval will be presented (Table 8.59) along with the quartile summary statistics; where they are not-estimable they will be denoted as “NA” for Not Available. A Kaplan Meier plot of time to first relapse will also be provided (Figure 8.08). Time to first relapse will be

calculated as the relapse onset date minus date study medication was started plus one day. For subjects that do not relapse, their time to relapse will be censored at the date of withdrawal for subjects that withdraw from the study during the Treatment Phase or the Week 48 visit date for subjects that did not withdraw prematurely.

12.8. Other Safety Measures

12.8.1. Vital Signs

The following vital signs measurements will be collected at each study assessment: systolic and diastolic blood pressure, heart rate and temperature. Height will be measured at screening only. Vital signs will be listed by subject (Listing 8.36) and summarised by treatment group, treatment phase and scheduled assessment (Table 8.60). Parameters will be displayed in tables in the following order: SBP, DBP, Weight, HR and temperature. In addition, a summary of change from baseline at all scheduled post-baseline assessments in vital signs will also be produced (Table 8.61).

The number and percentage of subjects in each dose group with values of blood pressure, and heart rate outside pre-determined potentially clinically important ranges and with changes from baseline of potential clinical concern (see Section 9.4.2 for details on absolute values and changes from baseline of potential clinical concern) will be tabulated at each scheduled assessment and for the maximum on-treatment values (Table 8.62). All vital signs data of potential clinical concern will be listed (Listing 8.37).

Change from baseline in vital signs will also be displayed graphically. Boxplots will be produced for each parameter that will display the distribution of each change in vital sign at each scheduled assessment up to the end of the follow-up period and the maximum on treatment change (Figure 8.09).

12.8.2. ECG Assessments

ECGs are collected and read by a central ECG reader at Screening, and Weeks 2, 4, 12, 24, 36 and 48. A summary of the clinical interpretation of the ECG according to the investigator, number and percentage of subjects who had abnormal and/or clinically significant ECG findings will be presented by treatment group (Table 8.63). A summary of ECG parameters (HR, PR, RR, QRS, QT, QTcF and QTcB) and change from baseline in each ECG parameter will also be presented by treatment group and visit; including the maximum on-treatment value (Table 8.64 and Table 8.65). In addition, a summary of the number of subjects with ECG parameters of potential clinical concern and changes of potential clinical concern (see Section 9.4.3) will also be provided by treatment group and visit (Table 8.66 and Table 8.67).

A listing of ECG values will be presented by treatment group and subject (Listing 8.39) and a listing of ECG interpretation according to the investigator will also be presented by treatment group and subject (Listing 8.38).

The distribution of change from baseline and the maximum on treatment change in QTcB and QTcF by time will be displayed in Figure 8.10.

12.8.3. Columbia Suicide Severity Rating Scale (C-SSRS)

The electronic Columbia–Suicide Severity Rating Scale (eC-SSRS) is a measure of suicidal ideation and behaviour completed by the subject as an automated telephone interview.

The following outcomes are eC-SSRS categories and have binary responses (yes/no). The categories have been re-ordered from the actual scale in an increasing order of severity from 1 to 9 to facilitate the definitions of the comparative endpoints.

- Category 1 – Wish to be Dead
- Category 2 – Non-specific Active Suicidal Thoughts
- Category 3 – Active Suicidal Ideation with Any Methods (Not Plan) without Intent to Act
- Category 4 – Active Suicidal Ideation with Some Intent to Act, without Specific Plan
- Category 5 – Active Suicidal Ideation with Specific Plan and Intent
- Category 6 – Preparatory Acts or Behaviour
- Category 7 – Aborted Attempt
- Category 8 – Interrupted Attempt
- Category 9 – Actual Attempt (non-fatal)

The following outcomes are numerical scores derived from the eC-SSRS categories. The scores are created at each assessment for each patient.

- Suicidal Ideation Score: The maximum suicidal ideation category (1-5 on the eC-SSRS) present at the assessment for all assessments following the Baseline assessment (the assessment at Baseline is a lifetime score). Assign a score of 0 if no ideation is present.
- Suicidal Behaviour Score: The maximum suicidal behaviour category (6-9 on the eC-SSRS) present at the assessment for all assessments following the Baseline assessment (the assessment at Baseline is a lifetime score). Assign a score of 0 if no behaviour is present.
- Suicidal Ideation or Behaviour Score: The maximum suicidal ideation or behaviour category (1-9 on the eC-SSRS) present at the assessment for all assessments following the Baseline assessment (the assessment at Baseline is a lifetime score). Assign a score of 0 if no ideation or behaviour is present.

A summary table of the number (and percentage) of subjects within each category for Suicidal Ideation, Suicidal Behaviour and Suicidal Ideation or Behaviour Score will be provided (Table 8.68) for completed eC-SSRS assessments. All eC-SSRS Categories data will be listed for completed eC-SSRS assessments (Listing 8.40 to Listing 8.42). A separate listing of incomplete eC-SSRS assessments will be provided (Listing 8.43). If the eC-SSRS could not be completed then an Investigator led assessment was to be conducted, all Investigator led assessments will be listed separately (Listing 8.44).

13. HEALTH OUTCOMES ANALYSES

13.1. MSQoL54

13.1.1. Summary Displays

All MSQoL54 data will be listed for the Randomised Population. Listing 9.01 will list all responses and scores for each administration of MSQoL54. The listing will be sorted by treatment group, centre, subject, visit and subscale ordered as per Table 9 in Section 9.2.10. Listing 9.02 will list each subscale score ordered as per listing 9.01. Listing 9.03 will list the Composite Scores. For each listing, a "*" will be used to denote post-baseline MSQoL54 assessments that were conducted at least 14 days after the last dose of study medication was taken.

Summary statistics (n, mean, standard deviation, median, minimum and maximum) for each MSQoL54 subscale will be provided at each visit, and for change from baseline at Week 48 in Table 9.01. A frequency count of the number subjects responding in each category for the two single-items will be provided (Table 9.02). Summary statistics for the Composite Scores will be summarised at each visit and for the change from baseline in Table 9.03. In addition, a summary table of the number of subjects at Week 48 that showed an improvement (by ≥10 point, by <10 - ≥5 point, <5 - ≥3 point and <3 point increases), worsening (by ≥10 point, by <10 - ≥5 point, <5 - ≥3 point and <3 pointdecreases) or unchanged relative to their baseline assessment for each Composite Score will be provided (Table 9.04).

13.1.2. Statistical Analysis

The change from baseline in MSQoL54 Composite Scores at Week 48 will be analysed using a parametric analysis of covariance (a linear model assuming normal errors) which will include terms for the baseline MSQoL54 Composite Score (as appropriate), Background Disease Modifying Therapy and treatment group (fitted as a categorical variable). Pair wise comparisons will be conducted for GSK239512 versus placebo (Table 9.05 and Table 9.06).

The normality assumptions underpinning the analysis will be assessed by inspection of the following plots:

- Histogram of marginal studentised residuals derived from the MMRM model
- Q-Q Plots
- Scatter plot of studentised residuals against fitted values
- Scatter plot of studentised residuals against each of the model covariates
- Normal probability plot with simulation envelope

If the residuals indicate non-normality of the model, the van Elteren extension to the Wilcoxon rank sum test, stratifying for Background Disease Modifying Therapy may be performed as a non parametric sensitivity analysis.

14. CLINICAL PHARMACOLOGY DATA ANALYSES

PK analysis and PK/PD exploration analysis will be conducted under the management of Clinical Pharmacology Modelling & Simulation (CPMS). Since this is a single dose level study, population PK and population PK/PD analyses are not planned.

The proposed PK tables, figures and listings for PK and PK/PD are shown in Section 16 of the RAP

14.1. Pharmacokinetic Analyses

The reconciliation of the PK Case Report Form (CRF) and SMS2000 data will be performed by, or under the direct auspices of Clinical Data Management (CDM), GlaxoSmithKline. The merging of PK concentration data, randomisation and CRF data will be performed by, or under the direct auspices of the Biostatistics and Programming Team (BPT), GlaxoSmithKline.

Pre-dose trough concentrations of GSK239512 will be summarized descriptively by week (i.e. 4, 8, 24, 36 and 48) and by dose level. Concentrations of GSK239512 at week 8 will be summarized descriptively by nominal sampling time (i.e. pre-dose and at 0.5, 2 and 6 hours post-dose) and dose level.

14.2. Pharmacokinetic/Pharmacodynamic Analyses

The relationship between the co-primary study endpoints and the GSK239512 trough concentrations during the maintenance stage will be explored graphically. In the presence of a relationship, a PK/PD modeling approach may be used to quantify the relationship.

For those subjects whose dose remained constant during the maintenance, the trough concentration to be used PK/PD analysis will be the average trough concentration during the maintenance phase. For any subject whose dose changed during the maintenance phase, the GSK239512 trough concentrations will be the concentration or average the concentration at the dose level that the subject was on at the time that the lesion occurred and is deemed to reflect the GSK239512 dose during the remyelination period.

15. REFERENCES

Barnard J, Rubin D. Small Sample Degrees of Freedom with Multiple Imputations. *Biometrika.* 1999;86:948-955.

Carpenter JR, Roger JH, Kenward MG. Analysis of Longitudinal Trials with Protocol Deviations: a Framework for Relevant, Accessible Assumptions, and Inference via Multiple Imputation. *Journal of Biopharmaceutical Statistics (accepted for publication).* 2013;23:6:1352-1371 .

Efron B, The *efficiency of Cox's likelihood function for censored data*. Journal of the American Statistical Association. 1977; 72, 557-565.

GlaxoSmithKline Clinical Protocol Document Number HM2012N133918_00; Proof of Mechanism Study to Assess the Potential of GSK239512 to Remyelinate Lesions in Subjects with Relapsing Remitting Multiple Sclerosis H3M116477. Effective Date: 26 July 2012

Vickrey BG, Hays RD, Harooni R, Myers LW, Ellison GW. A health-related quality of life measure for multiple sclerosis. *Qual Life Res*. 1995; 4(3): 187-206.

16. ATTACHMENTS

16.1. Table of Contents for Data Display Specifications

Study Population Tables

Table No.	Pop	Table Title	Template Reference
6.001	R	Summary of Subjects by Population	**SA1**
6.002	ITT	Summary of Inclusion/Exclusion Criteria Deviations	**IE1**
6.003	ITT	Summary of Subject Disposition	**ES1**
6.004	ITT	Summary of Subject Visits	SP1
6.005	NR	Summary of Reasons for Screening Failures	SP2
6.006	ITT	Summary of Protocol Deviations	**DV1B**
6.007	ITT	Summary of Demographic Characteristics	**DM1**
6.008	ITT	Summary of Race and Racial Combinations	**DM5**
6.009	ITT	Summary of Race and Racial Combination Details	**DM6**
6.010	ITT	Summary of Past Medical Conditions	**MH1**
6.011	ITT	Summary of Current Medical Conditions	**MH1**
6.012	ITT	Summary of Family History of Cardiovascular Risk Factors	SP3
6.013	ITT	Summary of MS Medical History at Screening	NS_P02
6.014	ITT	Summary of Smoking History	**SU1**
6.015	ITT	Summary of Prior MS Disease Modifying Therapies	**CM1**
6.016	ITT	Summary of Prior Medications	**CM1**
6.017	ITT	Summary of Concomitant Medications (excluding required MS Disease Modifying Therapy) taken during the Titration Period	**CM1**
6.018	ITT	Summary of Concomitant Medications (excluding required MS Disease Modifying Therapy) taken during the Maintenance Period	**CM1**
6.019	ITT	Summary of Concomitant Medications (excluding required MS Disease Modifying Therapy) taken during the Treatment Phase	**CM1**
6.020	ITT	Summary of Medications Initiated during the Follow-Up Period	**CM1**
6.021	ITT	Summary of Prohibited Medications taken during the Treatment Phase	**CM1**
6.022	ITT	Summary of Exposure	SAT01

Table No.	Pop	Table Title	Template Reference
6.023	ITT	Summary of Treatment Compliance	NS_COMP1
6.024	ITT	Summary of Dose Titration	SP3
6.025	ITT	Summary of Treatment Compliance during the Titration Period	NS_COMP1
6.026	ITT	Summary of Treatment Compliance during the Maintenance Period	NS_COMP1
6.027	ITT	Summary of MS Medical History at Screening for Subjects with an MRI in the 12 Months Prior to Screening	See iSRC.

Study Population Listings

Listing No	Pop	Listing Title	Template Reference
6.001	R	Listing of Subjects Excluded from Populations	**SA3a**
6.002	R	Listing of Inclusion/Exclusion Criteria Deviations	**IE3**
6.003	R	Listing of Child Bearing Potential at Screening (Female Subjects only)	SP08
6.004	R	Listing of Subject Withdrawals	**ES2**
6.005	R	Listing of Subject Visits	NS_P07
6.006	R	Listing of Protocol Deviations	**DV2**
6.007	R	Listing of Subjects for Whom the Treatment Blind was Broken	**BL1**
6.008	R	Listing of Subjects Randomised to the Incorrect Strata	**BL1**
6.009	R	Listing of Demographic Characteristics	**DM2**
6.010	R	Listing of Race	**DM9**
6.011	R	Listing of Medical History	**MH2**
6.012	R	Listing of Family History of Cardiovascular Risk Factors	SP4
6.013	R	Listing of MS Medical History at Screening.	SP07
6.014	R	Listing of MS Medical History at Screening: Summary of Mrain MRI Scans in Previous 12 Months	SP07a
6.015	R	Listing of Previously Impacted Functional Systems during Relapses for Subjects Relapsing in the Study	SP5
6.016	R	Listing of Smoking History	**SU2**
6.017	R	Listing of Prior MS Disease Modifying Therapies	**CM3**
6.018	R	Listing of Prior/Concomitant Medications	**CM3**

Listing No	Pop	Listing Title	Template Reference
6.019	R	Relationship between ATC Level 1, Ingredient and Verbatim Text	**CM6**
6.020	R	Listing of Prohibited Medications taken during the Treatment Phase	**CM3**
6.021	R	Listing of Subjects Receiving Investigational Product	**TA1**
6.022	R	Listing of Exposure Data	**EX3**
6.023	R	Listing of Study Medication Dispensed and Tablets Returned	**EX3**
6.024	R	Listing of Compliance Data	SPL13
6.025	R	Listing of Subjects Receiving Incorrect Study Medication	SPL15

Study Population Figures

Figure No	Pop	Listing Title	Template Reference
6.001	ITT	Summary of Duration of Exposure to Study Medication	SAF01

Efficacy Tables

Table No.	Pop	Table Title	Template Reference
**MTR Summary Displays**			
7.001	ITT	Frequency Count of Number of Gadolinium-Enhancing Lesions with MTR-Assessments at Each MRI Assessment	NS_TE06
7.002	ITT	Frequency Count of Number of delta-MTR Lesions with MTR-Assessments at Each MRI Assessment	NS_TE06
7.003	ITT	Summary Statistics of MTR Values for Gadolinium-Enhancing Lesions by Relative MRI Scan	NS_TE07
7.004	ITT	Summary Statistics of MTR Values for delta-MTR Lesions by Relative MRI Scan	NS_TE07
7.005	ITT	Summary Statistics of Change in MTR Values from Reference MRI Scan MTR Value for Gadolinium-Enhancing Lesions by Relative MRI Scan	NS_TE07
7.006	ITT	Summary Statistics of Change in MTR Values from Reference MRI Scan MTR Value for for delta-MTR Lesions by Relative MRI Scan	NS_TE07

Table No.	Pop	Table Title	Template Reference
**MTR ANCOVA Analyses**			
7.007	ITT	Summary of the Statistical Analysis of the Change in MTR Value Post Lesion relative to Pre Lesion for Gadolinium-Enhancing Lesions Excluding MRI Scans Within 70 Days of Lesion Occurrence	NS_TE01
7.008	ITT	Posterior Probability of MTR Value Post Lesion relative to Pre Lesion for Gadolinium-Enhancing Lesions Excluding MRI Scans Within 70 Days of Lesion Occurrence and 90% Credible Intervals	Post2
7.009	ITT	Summary of the Statistical Analysis of the Change in MTR Value Post Lesion relative to Pre Lesion for Gadolinium-Enhancing Lesions Excluding MRI Scans Within 70 Days of Lesion Occurrence with at Least 2 MRI Assessments Pre- and Post-Lesion	NS_TE01
7.010	ITT	Posterior Probability of MTR Value Post Lesion relative to Pre Lesion for Gadolinium-Enhancing Lesions Excluding MRI Scans Within 70 Days of Lesion Occurrence with at Least 2 MRI Assessments Pre- and Post-Lesion and 90% Credible Intervals	Post2
7.011	ITT	Summary of the Statistical Analysis of the Change in MTR Value Post Lesion relative to Pre Lesion for Gadolinium-Enhancing Lesions Excluding MRI Scans Within 28 Days Prior to Lesion Occurrence and 70 Days Post Lesion Occurrence	NS_TE01
7.012	ITT	Summary of the Statistical Analysis of the Change in MTR Value Post Lesion relative to Pre Lesion for Gadolinium-Enhancing Lesions	NS_TE01
7.013	ITT	Summary of the Statistical Analysis of the Change in MTR Value Post Lesion relative to Pre Lesion for Gadolinium-Enhancing Lesions Excluding MRI Scans Within 28 Days of Lesion Occurrence	NS_TE01
7.014	ITT	Summary of the Statistical Analysis of the Change in MTR Value Post Lesion relative to Pre Lesion for Gadolinium-Enhancing Lesions Excluding MRI Scans Within 70 Days Prior to Lesion Occurrence and 28 Days Post Lesion Occurrence	NS_TE01
7.015	ITT	Summary of the Statistical Analysis of the Change in MTR Value Post Lesion relative to Pre Lesion for delta-MTR Lesions Excluding MRI Scans Within 70 Days of Lesion Occurrence	NS_TE01
7.016	ITT	Posterior Probability of MTR Value Post Lesion relative to Pre Lesion for delta-MTR Lesions Excluding MRI Scans Within 70 Days of Lesion Occurrence and 90% Credible Intervals	Post2
7.017	ITT	Summary of the Statistical Analysis of the Change in MTR Value Post Lesion relative to Pre Lesion for delta-MTR Lesions Excluding MRI Scans Within 70 Days of Lesion Occurrence for Lesions with at Least 2 MRI Assessments Pre- and Post-Lesion	NS_TE01
7.018	ITT	Posterior Probability of MTR Value Post Lesion relative to Pre Lesion for delta-MTR Lesions Excluding MRI Scans Within 70 Days of Lesion Occurrence with at Least 2 MRI Assessments Pre- and Post-Lesion and 90% Credible Intervals	Post2

Table No.	Pop	Table Title	Template Reference
7.019	ITT	Summary of the Statistical Analysis of the Change in MTR Value Post Lesion relative to Pre Lesion for delta-MTR Lesions Excluding MRI Scans Within 28 Days Prior to Lesion Occurrence and 70 Days Post Lesion Occurrence	NS_TE01
7.020	ITT	Summary of the Statistical Analysis of the Change in MTR Value Post Lesion relative to Pre Lesion for delta-MTR Lesions	NS_TE01
7.021	ITT	Summary of the Statistical Analysis of the Change in MTR Value Post Lesion relative to Pre Lesion for delta-MTR Lesions Excluding MRI Scans Within 28 Days of Lesion Occurrence	NS_TE01
7.022	ITT	Summary of the Statistical Analysis of the Change in MTR Value Post Lesion relative to Pre Lesion for delta-MTR Lesions Excluding MRI Scans Within 70 Days Prior to Lesion Occurrence and 28 Days Post Lesion Occurrence	NS_TE01
7.023	ITT	Summary of the Statistical Analysis of the Change in MTR Value Post Lesion relative to Pre Lesion for Gadolinium-Enhancing Lesions averaged for each Subject Excluding MRI Scans Within 70 Days of Lesion Occurrence	NS_TE01
7.024	ITT	Posterior Probability of MTR Value Post Lesion relative to Pre Lesion for Gadolinium-Enhancing Lesions averaged for each Subject Excluding MRI Scans Within 70 Days of Lesion Occurrence and 90% Credible Intervals	Post2
7.025	ITT	Summary of the Statistical Analysis of the Change in MTR Value Post Lesion relative to Pre Lesion for Gadolinium-Enhancing Lesions averaged for each Subject Excluding MRI Scans Within 70 Days of Lesion Occurrence with at Least 2 MRI Assessments Pre- and Post-Lesion	NS_TE01
7.026	ITT	Posterior Probability of MTR Value Post Lesion relative to Pre Lesion for Gadolinium-Enhancing Lesions averaged for each Subject Excluding MRI Scans Within 70 Days of Lesion Occurrence with at Least 2 MRI Assessments Pre- and Post-Lesion and 90% Credible Intervals	Post2
7.027	ITT	Summary of the Statistical Analysis of the Change in MTR Value Post Lesion relative to Pre Lesion for Gadolinium-Enhancing Lesions averaged for each Subject Excluding MRI Scans Within 28 Days Prior to Lesion Occurrence and 70 Days Post Lesion Occurrence	NS_TE01
7.028	ITT	Summary of the Statistical Analysis of the Change in MTR Value Post Lesion relative to Pre Lesion for Gadolinium-Enhancing Lesions averaged for each Subject	NS_TE01
7.029	ITT	Summary of the Statistical Analysis of the Change in MTR Value Post Lesion relative to Pre Lesion for Gadolinium-Enhancing Lesions averaged for each Subject Excluding MRI Scans Within 28 Days of Lesion Occurrence	NS_TE01

Table No.	Pop	Table Title	Template Reference
7.030	ITT	Summary of the Statistical Analysis of the Change in MTR Value Post Lesion relative to Pre Lesion for Gadolinium-Enhancing Lesions averaged for each Subject Excluding MRI Scans Within 70 Days Prior to Lesion Occurrence and 28 Days Post Lesion Occurrence	NS_TE01
7.031	ITT	Summary of the Statistical Analysis of the Change in MTR Value Post Lesion relative to Pre Lesion for delta-MTR Lesions averaged for each Subject Excluding MRI Scans Within 70 Days of Lesion Occurrence	NS_TE01
7.032	ITT	Posterior Probability of MTR Value Post Lesion relative to Pre Lesion for delta-MTR Lesions averaged for each Subject Excluding MRI Scans Within 70 Days of Lesion Occurrence and 90% Credible Intervals	Post2
7.033	ITT	Summary of the Statistical Analysis of the Change in MTR Value Post Lesion relative to Pre Lesion for delta-MTR Lesions averaged for each Subject Excluding MRI Scans Within 70 Days of Lesion Occurrence for Lesions with at Least 2 MRI Assessments Pre- and Post-Lesion	NS_TE01
7.034	ITT	Posterior Probability of MTR Value Post Lesion relative to Pre Lesion for delta-MTR Lesions averaged for each Subject Excluding MRI Scans Within 70 Days of Lesion Occurrence with at Least 2 MRI Assessments Pre- and Post-Lesion and 90% Credible Intervals	Post2
7.035	ITT	Summary of the Statistical Analysis of the Change in MTR Value Post Lesion relative to Pre Lesion for delta-MTR Lesions averaged for each Subject Excluding MRI Scans Within 28 Days Prior to Lesion Occurrence and 70 Days Post Lesion Occurrence	NS_TE01
7.036	ITT	Summary of the Statistical Analysis of the Change in MTR Value Post Lesion relative to Pre Lesion for delta-MTR Lesions averaged for each Subject	NS_TE01
7.037	ITT	Summary of the Statistical Analysis of the Change in MTR Value Post Lesion relative to Pre Lesion for delta-MTR Lesions averaged for each Subject Excluding MRI Scans Within 28 Days of Lesion Occurrence	NS_TE01
7.038	ITT	Summary of the Statistical Analysis of the Change in MTR Value Post Lesion relative to Pre Lesion for delta-MTR Lesions averaged for each Subject Excluding MRI Scans Within 70 Days Prior to Lesion Occurrence and 28 Days Post Lesion Occurrence	NS_TE01
**MTR MMRM Analyses**			
7.039	ITT	Summary of the Repeated Measures Analysis of the Change in MTR Value Post Lesion relative to Pre Lesion for Gadolinium-Enhancing Lesions Excluding MRI Scans Within 70 Days of Lesion Occurrence	NS_TE01
7.040	ITT	Posterior Probability based on the Repeated Measures Analysis of MTR Value Post Lesion relative to Pre Lesion for Gadolinium-Enhancing Lesions Excluding MRI Scans Within 70 Days of Lesion Occurrence and 90% Credible Intervals	Post2

Table No.	Pop	Table Title	Template Reference
7.041	ITT	Summary of the Repeated Measures Analysis of the Change in MTR Value Post Lesion relative to Pre Lesion for Gadolinium-Enhancing Lesions Excluding MRI Scans Within 70 Days of Lesion Occurrence with at Least 2 MRI Assessments Pre- and Post-Lesion	NS_TE01
7.042	ITT	Posterior Probability based on the Repeated Measures Analysis of MTR Value Post Lesion relative to Pre Lesion for Gadolinium-Enhancing Lesions Excluding MRI Scans Within 70 Days of Lesion Occurrence with at Least 2 MRI Assessments Pre- and Post-Lesion and 90% Credible Intervals	Post2
7.043	ITT	Summary of the Repeated Measures Analysis of the Change in MTR Value Post Lesion relative to Pre Lesion for Gadolinium-Enhancing Lesions Excluding MRI Scans Within 28 Days Prior to Lesion Occurrence and 70 Days Post Lesion Occurrence	NS_TE01
7.044	ITT	Summary of the Repeated Measures Analysis of the Change in MTR Value Post Lesion relative to Pre Lesion for Gadolinium-Enhancing Lesions	NS_TE01
7.045	ITT	Summary of the Repeated Measures Analysis of the Change in MTR Value Post Lesion relative to Pre Lesion for Gadolinium-Enhancing Lesions Excluding MRI Scans Within 28 Days of Lesion Occurrence	NS_TE01
7.046	ITT	Summary of the Repeated Measures Analysis of the Change in MTR Value Post Lesion relative to Pre Lesion for Gadolinium-Enhancing Lesions Excluding MRI Scans Within 70 Days Prior to Lesion Occurrence and 28 Days Post Lesion Occurrence	NS_TE01
7.047	ITT	Summary of the Repeated Measures Analysis of the Change in MTR Value Post Lesion relative to Pre Lesion for delta-MTR Lesions Excluding MRI Scans Within 70 Days of Lesion Occurrence	NS_TE01
7.048	ITT	Posterior Probability based on the Repeated Measures Analysis of MTR Value Post Lesion relative to Pre Lesion for delta-MTR Lesions Excluding MRI Scans Within 70 Days of Lesion Occurrence and 90% Credible Intervals	Post2
7.049	ITT	Summary of the Repeated Measures Analysis of the Change in MTR Value Post Lesion relative to Pre Lesion for delta-MTR Lesions Excluding MRI Scans Within 70 Days of Lesion Occurrence for Lesions with at Least 2 MRI Assessments Pre- and Post-Lesion	NS_TE01
7.050	ITT	Posterior Probability based on the Repeated Measures Analysis of MTR Value Post Lesion relative to Pre Lesion for delta-MTR Lesions Excluding MRI Scans Within 70 Days of Lesion Occurrence with at Least 2 MRI Assessments Pre- and Post-Lesion and 90% Credible Intervals	Post2
7.051	ITT	Summary of the Repeated Measures Analysis of the Change in MTR Value Post Lesion relative to Pre Lesion for delta-MTR Lesions Excluding MRI Scans Within 28 Days Prior to Lesion Occurrence and 70 Days Post Lesion Occurrence	NS_TE01

Table No.	Pop	Table Title	Template Reference
7.052	ITT	Summary of the Repeated Measures Analysis of the Change in MTR Value Post Lesion relative to Pre Lesion for delta-MTR Lesions	NS_TE01
7.053	ITT	Summary of the Repeated Measures Analysis of the Change in MTR Value Post Lesion relative to Pre Lesion for delta-MTR Lesions Excluding MRI Scans Within 28 Days of Lesion Occurrence	NS_TE01
7.054	ITT	Summary of the Repeated Measures Analysis of the Change in MTR Value Post Lesion relative to Pre Lesion for delta-MTR Lesions Excluding MRI Scans Within 70 Days Prior to Lesion Occurrence and 28 Days Post Lesion Occurrence	NS_TE01
7.055	ITT	Summary of the Repeated Measures Analysis of the Change in MTR Value Post Lesion relative to Pre Lesion for Gadolinium-Enhancing Lesions averaged for each Subject Excluding MRI Scans Within 70 Days of Lesion Occurrence	NS_TE01
7.056	ITT	Posterior Probability based on the Repeated Measures Analysis of MTR Value Post Lesion relative to Pre Lesion for Gadolinium-Enhancing Lesions averaged for each Subject Excluding MRI Scans Within 70 Days of Lesion Occurrence and 90% Credible Intervals	Post2
7.057	ITT	Summary of the Repeated Measures Analysis of the Change in MTR Value Post Lesion relative to Pre Lesion for Gadolinium-Enhancing Lesions averaged for each Subject Excluding MRI Scans Within 70 Days of Lesion Occurrence with at Least 2 MRI Assessments Pre- and Post-Lesion	NS_TE01
7.058	ITT	Posterior Probability based on the Repeated Measures Analysis of MTR Value Post Lesion relative to Pre Lesion for Gadolinium-Enhancing Lesions averaged for each Subject Excluding MRI Scans Within 70 Days of Lesion Occurrence with at Least 2 MRI Assessments Pre- and Post-Lesion and 90% Credible Intervals	Post2
7.059	ITT	Summary of the Repeated Measures Analysis of the Change in MTR Value Post Lesion relative to Pre Lesion for Gadolinium-Enhancing Lesions averaged for each Subject Excluding MRI Scans Within 28 Days Prior to Lesion Occurrence and 70 Days Post Lesion Occurrence	NS_TE01
7.060	ITT	Summary of the Repeated Measures Analysis of the Change in MTR Value Post Lesion relative to Pre Lesion for Gadolinium-Enhancing Lesions averaged for each Subject	NS_TE01
7.061	ITT	Summary of the Repeated Measures Analysis of the Change in MTR Value Post Lesion relative to Pre Lesion for Gadolinium-Enhancing Lesions averaged for each Subject Excluding MRI Scans Within 28 Days of Lesion Occurrence	NS_TE01

Table No.	Pop	Table Title	Template Reference
7.062	ITT	Summary of the Repeated Measures Analysis of the Change in MTR Value Post Lesion relative to Pre Lesion for Gadolinium-Enhancing Lesions averaged for each Subject Excluding MRI Scans Within 70 Days Prior to Lesion Occurrence and 28 Days Post Lesion Occurrence	NS_TE01
7.063	ITT	Summary of the Repeated Measures Analysis of the Change in MTR Value Post Lesion relative to Pre Lesion for delta-MTR Lesions averaged for each Subject Excluding MRI Scans Within 70 Days of Lesion Occurrence	NS_TE01
7.064	ITT	Posterior Probability based on the Repeated Measures Analysis of MTR Value Post Lesion relative to Pre Lesion for delta-MTR Lesions averaged for each Subject Excluding MRI Scans Within 70 Days of Lesion Occurrence and 90% Credible Intervals	Post2
7.065	ITT	Summary of the Repeated Measures Analysis of the Change in MTR Value Post Lesion relative to Pre Lesion for delta-MTR Lesions averaged for each Subject Excluding MRI Scans Within 70 Days of Lesion Occurrence for Lesions with at Least 2 MRI Assessments Pre- and Post-Lesion	NS_TE01
7.066	ITT	Posterior Probability based on the Repeated Measures Analysis of MTR Value Post Lesion relative to Pre Lesion for delta-MTR Lesions averaged for each Subject Excluding MRI Scans Within 70 Days of Lesion Occurrence with at Least 2 MRI Assessments Pre- and Post-Lesion and 90% Credible Intervals	Post2
7.067	ITT	Summary of the Repeated Measures Analysis of the Change in MTR Value Post Lesion relative to Pre Lesion for delta-MTR Lesions averaged for each Subject Excluding MRI Scans Within 28 Days Prior to Lesion Occurrence and 70 Days Post Lesion Occurrence	NS_TE01
7.068	ITT	Summary of the Repeated Measures Analysis of the Change in MTR Value Post Lesion relative to Pre Lesion for delta-MTR Lesions averaged for each Subject	NS_TE01
7.069	ITT	Summary of the Repeated Measures Analysis of the Change in MTR Value Post Lesion relative to Pre Lesion for delta-MTR Lesions averaged for each Subject Excluding MRI Scans Within 28 Days of Lesion Occurrence	NS_TE01
7.070	ITT	Summary of the Repeated Measures Analysis of the Change in MTR Value Post Lesion relative to Pre Lesion for delta-MTR Lesions averaged for each Subject Excluding MRI Scans Within 70 Days Prior to Lesion Occurrence and 28 Days Post Lesion Occurrence	NS_TE01
**Conventional MRI**			
7.071	ITT	Summary Statistics of the Actual Number of New Gadolinium Enhancing Lesions (OC Dataset)	NS_TE04

Table No.	Pop	Table Title	Template Reference
7.072	ITT	Frequency Table of the Actual Number of New Gadolinium-Enhancing Lesions (OC Dataset)	NS_TE05
7.073	ITT	Summary Statistics of the Cumulative Number of New Gadolinium Enhancing Lesions (AES Dataset)	NS_TE04
7.074	ITT	Frequency Table of the Cumulative Number of New Gadolinium-Enhancing Lesions (OC Dataset)	NS_TE05
7.075	ITT	Summary Statistics of the Actual Number of New Enlarging T2 Lesions (OC Dataset)	NS_TE04
7.076	ITT	Frequency Table of the Actual Number of New Enlarging T2Lesions (OC Dataset)	NS_TE05
7.077	ITT	Summary Statistics of the Cumulative Number of New Enlarging T2 Lesions (AES Dataset)	NS_TE04
7.078	ITT	Frequency Table of the Cumulative Number of New Enlarging T2 Lesions (OC Dataset)	NS_TE05
7.079	ITT	Summary Statistics of the Actual Number of Cumulative Unique Active Lesions (OC Dataset)	NS_TE04
7.080	ITT	Frequency Table of the Actual Number of Cumulative Unique Active Lesions (OC Dataset)	NS_TE05
7.081	ITT	Summary Statistics of the Cumulative Number of Cumulative Unique Active Lesions (AES Dataset)	NS_TE04
7.082	ITT	Frequency Table of the Cumulative Number of Cumulative Unique Active Lesions (OC Dataset)	NS_TE05
7.083	ITT	Summary Statistics of the Actual Number of New Unenhancing T1 Lesions (OC Dataset)	NS_TE04
7.084	ITT	Frequency Table of the Actual Number of New Unenhancing T1 Lesions (OC Dataset)	NS_TE05
7.085	ITT	Summary Statistics of the Cumulative Number of New Unenhancing T1 Lesions (AES Dataset)	NS_TE04
7.086	ITT	Frequency Table of the Cumulative Number of New Unenhancing T1 Lesions (OC Dataset)	NS_TE05
7.087	ITT	Summary Statistics of the Actual Number of New Gadolinium Enhancing Lesions Evolving into Black Holes by Week 48 (OC Dataset)	NS_TE04
7.088	ITT	Frequency Table of the Actual Number of New Gadolinium-Enhancing Lesions Evolving into Black Holes by Week 48 (OC Dataset)	NS_TE05

Table No.	Pop	Table Title	Template Reference
7.089	ITT	Summary Statistics of the Cumulative Number of New Gadolinium Enhancing Lesions Evolving into Black Holes by Week 48 (AES Dataset)	NS_TE04
7.090	ITT	Frequency Table of the Cumulative Number of New Gadolinium-Enhancing Lesions Evolving into Black Holes by Week 48 (OC Dataset)	NS_TE05
7.091	ITT	Summary of the Statistical Analysis of the Cumulative Number of New Gadolinium-Enhancing Lesions at Week 48 (AES Dataset)	NS_TE01
7.092	ITT	Summary of the Statistical Analysis of the Cumulative Number of New Gadolinium-Enhancing Lesions at Week 48 using Multiple Imputation Methods (OC Dataset)	NS_TE01a
7.093	ITT	Summary of the Statistical Analysis of the Cumulative Number of New Enlarging T2 Lesions at Week 48 (AES Dataset)	NS_TE01
7.094	ITT	Summary of the Statistical Analysis of the Cumulative Number of New Enlarging T2 Lesions at Week 48 using Multiple Imputation Methods (AES Dataset)	NS_TE01a
7.095	ITT	Summary of the Statistical Analysis of the Cumulative Number of Cumulative Unique Active Lesions at Week 48 (AES Dataset)	NS_TE01
7.096	ITT	Summary of the Statistical Analysis of the Cumulative Number of Cumulative Unique Active Lesions at Week 48 using Multiple Imputation Methods (AES Dataset)	NS_TE01a
7.097	ITT	Summary of the Statistical Analysis of the Cumulative Number of Unenhancing T1 Lesions at Week 48 (AES Dataset)	NS_TE01
7.098	ITT	Summary of the Statistical Analysis of the Cumulative Number of Unenhancing T1 Lesions at Week 48 using Multiple Imputation Methods (AES Dataset)	NS_TE01a
7.099	ITT	Summary of the Statistical Analysis of the Cumulative Number of New Gadolinium-Enhancing Lesions Evolving into Black Holes at Week 48 (AES Dataset)	NS_TE01
7.100	ITT	Summary of the Statistical Analysis of the Cumulative Number of New Gadolinium-Enhancing Lesions Evolving into Black Holes at Week 48 using Multiple Imputation Methods (AES Dataset)	NS_TE01a
**Conventional MRI Volumes**			
7.101	ITT	Summary Statistics of Normalised Brain Volume (OC Dataset)	NS_TE04
7.102	ITT	Summary Statistics of Whole Brain Atrophy Volume Change from Screening (AES Dataset)	NS_TE04
7.103	ITT	Summary Statistics of White Matter Volume at each MRI (OC Dataset)	NS_TE04

Table No.	Pop	Table Title	Template Reference
7.104	ITT	Summary Statistics of Change from Screening in White Matter Volume at each MRI (AES Dataset)	NS_TE04
7.105	ITT	Summary Statistics of Grey Matter Volume at each MRI (OC Dataset)	NS_TE04
7.106	ITT	Summary Statistics of Change from Screening in Grey Matter Volume at each MRI (AES Dataset)	NS_TE04
7.107	ITT	Summary of the Analysis of Covariance of the Whole Brain Atrophy Volume Change from Screening at Week 48 (AES Dataset)	NS_TE01
7.108	ITT	Summary of the Analysis of Covariance of White Matter Volume Change from Screening at Week 48 (AES Dataset)	NS_TE01
7.109	ITT	Summary of the Analysis of Covariance of Grey Matter Volume Change from Screening at Week 48 (AES Dataset)	NS_TE01
7.110	ITT	Summary of Repeated Measures Analysis of the Change from Screening in White Matter Volume at each MRI (AES Dataset)	NS_TE01
7.111	ITT	Summary of Repeated Measures Analysis of the Change from Screening in Grey Matter Volume at each MRI (AES Dataset)	NS_TE01
**CogState**			
7.112	ITT	Summary of CogState Battery Task Primary Measure Baseline Raw Scores Relative to a Normal Population	NS_CG01
7.113	ITT	Frequency Count of CogState Battery Tasks at each Assessment Meeting Completion or Integrity Failure Criterion	NS_CG02
7.114	ITT	Summary of CogState Battery Task Primary Measure Raw Scores by Visit	EFT01
7.115	ITT	Summary of CogState Battery Task Primary Measure Standardised Scores by Visit	EFT01
7.116	ITT	Summary of CogState Battery Total Score by Visit	EFT01
7.117	ITT	Summary of CogState Battery Executive Function Composite Score by Visit	EFT01
7.118	ITT	Summary of CogState Battery Memory Composite Score by Visit	EFT01
7.119	ITT	Summary of CogState Battery Attention Composite Score by Visit	EFT01
7.120	ITT	Summary of Repeated Measures Analysis of Change from Baseline in CogState Battery Total Score	CG2
7.121	ITT	Posterior Probability for Change from Baseline in CogState Battery Total Score and 90% Credible Intervals at Week 48	Post2

Table No.	Pop	Table Title	Template Reference
7.122	ITT	Summary of Repeated Measures Analysis of Change from Baseline in CogState Battery Total Score by Background Disease Modifying Therapy Stratum	CG2
7.123	ITT	Summary of Analysis of Covariance of CogState Battery Total Score at Week 48	CG2
7.124	ITT	Summary of Analysis of Covariance of CogState Battery Total Score at Week 48 (Multiple Imputed Dataset – MAR, CDC and Delta approaches)	CG2a
7.125	ITT	Summary of Repeated Measures Analysis of Change from Baseline in CogState Battery Executive Function Composite Score	CG2
7.126	ITT	Posterior Probability for Change from Baseline in CogState Battery Executive Function Composite Score and 90% Credible Intervals at Week 48	Post2
7.127	ITT	Summary of Repeated Measures Analysis of Change from Baseline in CogState Battery Executive Function Composite Score by Background Disease Modifying Therapy Stratum	CG2
7.128	ITT	Summary of Analysis of Covariance of CogState Battery Executive Function Composite Score at Week 48	CG2
7.129	ITT	Summary of Analysis of Covariance of CogState Battery Executive Function Composite Score at Week 48 (Multiple Imputed Dataset – MAR, CDC and Delta approaches)	CG2a
7.130	ITT	Summary of Repeated Measures Analysis of Change from Baseline in CogState Battery Memory Composite Score	CG2
7.131	ITT	Posterior Probability for Change from Baseline in CogState Battery Memory Composite Score and 90% Credible Intervals at Week 48	Post2
7.132	ITT	Summary of Repeated Measures Analysis of Change from Baseline in CogState Battery Memory Composite Score by Background Disease Modifying Therapy Stratum	CG2
7.133	ITT	Summary of Analysis of Covariance of CogState Battery Memory Composite Score at Week 48	CG2
7.134	ITT	Summary of Analysis of Covariance of CogState Battery Memory Composite Score at Week 48 (Multiple Imputed Dataset – MAR, CDC and Delta approaches)	CG2a
7.135	ITT	Summary of Repeated Measures Analysis of Change from Baseline in CogState Battery Attention Composite Score	CG2
7.136	ITT	Posterior Probability for Change from Baseline in CogState Battery Attention Composite Score and 90% Credible Intervals at Week 48	Post2
7.137	ITT	Summary of Repeated Measures Analysis of Change from Baseline in CogState Battery Attention Composite Score by Background Disease Modifying Therapy Stratum	CG2

Table No.	Pop	Table Title	Template Reference
7.138	ITT	Summary of Analysis of Covariance of CogState Battery Attention Composite Score at Week 48	CG2
7.139	ITT	Summary of Analysis of Covariance of CogState Battery Memory Composite Score at Week 48 (Multiple Imputed Dataset – MAR, CDC and Delta approaches)	CG2a
7.140	ITT	Summary of Repeated Measures Analysis of Change from Baseline in CogState Battery Groton Maze Learning Test Standardised Test Score	CG2
7.141	ITT	Summary of Repeated Measures Analysis of Change from Baseline in CogState Battery One Back Test Standardised Test Score	CG2
7.142	ITT	Summary of Repeated Measures Analysis of Change from Baseline in CogState Battery International Shopping List - Immediate Recall Task Standardised Test Score	CG2
7.143	ITT	Summary of Repeated Measures Analysis of Change from Baseline in CogState Battery International Shopping List - Delayed Recall Task Standardised Test Score	CG2
7.144	ITT	Summary of Repeated Measures Analysis of Change from Baseline in CogState Battery One Card Learning Task Standardised Test Score	CG2
7.145	ITT	Summary of Repeated Measures Analysis of Change from Baseline in CogState Battery Detection Task Standardised Test Score	CG2
7.146	ITT	Summary of Repeated Measures Analysis of Change from Baseline in CogState Battery Identification Task Standardised Test Score	CG2
**T2 Lesion MTR**			
7.147	ITT	Summary Statistics of T2 Lesion MTR Values by Visit	NS_TE07
7.148	ITT	Summary Statistics of Change from Screening in T2 Lesion MTR Values by Visit	NS_TE07
7.149	ITT	Summary of Repeated Measures Analysis of Change from Screening in T2 Lesion MTR Values	CG2
7.150	ITT	Posterior Probability for Change from Screening in T2 Lesion MTR Values and 90% Credible Intervals at Week 48	Post2

Efficacy Listings

Listing No	Pop	Listing Title	Template Reference
7.001	R	Listing of MTR Value Data	EL1
7.002	R	Listing of Derived Lesion Level MTR Value Data	EL2

Listing No	Pop	Listing Title	Template Reference
7.003	R	Listing of Derived Subject Level MTR Value Data	EL2
7.004	R	Listing of Subjects with no Lesions for MTR Analyses	EL3
7.005	R	Listing of Number of New Gadolinium Enhancing Lesions	NS_LE02
7.006	R	Listing of Number of New Enlarging T2 Lesions	NS_LE02
7.007	R	Listing of Number of Cumulative Unique Active Lesions	NS_LE02
7.008	R	Listing of Number of New Unenhancing T1 Lesions	NS_LE02
7.009	R	Listing of Number of Gadolinium-Enhancing Lesions Evolving to Black Holes at Week 48	NS_LE02
7.010	R	Listing of Normalised Brain Volume and Change from Screening in Atrophy at Week 48	NS_LE03
7.011	R	Listing of White Matter Volume	NS_LE03
7.012	R	Listing of Grey Matter Volume	NS_LE03
7.013	R	Listing of CogState Battery Task Data	
7.014	R	Listing of CogState Battery Standardised Task Scores	
7.015	R	Listing of CogState Battery	
7.016	R	Listing of T2 Lesion MTR Data	EL1

Efficacy Figures

Figure No	Pop	Listing Title	Template Reference
**MTR**			
7.001	ITT	Raw Mean MTR Value and 90% Confidence Interval for Gadolinium-Enhancing Lesions by Relative MRI Scan	FE01a
7.002	ITT	Raw Mean MTR Value and 90% Confidence Interval for delta-MTR Lesions by Relative MRI Scan	FE01a
7.003	ITT	Raw Mean Change from Reference MRI MTR Value and 90% Confidence Interval for Gadolinium-Enhancing Lesions by Relative MRI Scan	FE01a
7.004	ITT	Raw Mean Change from Reference MRI MTR Value and 90% Confidence Interval for delta-MTR Lesions by Relative MRI Scan	FE01a
7.005	ITT	Profile Plot of all Gadolinium-Enhancing Lesions with MTR Measurements by Subject	None

Figure No	Pop	Listing Title	Template Reference
7.057	ITT	Profile Plot of all delta-MTR Lesions with MTR Measurements by Subject	None
7.006	ITT	Posterior Probability of MTR Value Post Lesion relative to Pre Lesion for gadolinium-enhancing Lesions Excluding MRI Scans Within 70 Days of Lesion Occurrence Based on ANCOVA Statistical Analysis	Fig1
7.007	ITT	Posterior Probability of MTR Value Post Lesion relative to Pre Lesion for gadolinium-enhancing Lesions Excluding MRI Scans Within 70 Days of Lesion Occurrence with at Least 2 MRI Assessments Pre- and Post-Lesion Based on ANCOVA Statistical Analysis	Fig1
7.008	ITT	Posterior Probability of MTR Value Post Lesion relative to Pre Lesion for delta-MTR Lesions Excluding MRI Scans Within 70 Days of Lesion Occurrence Based on ANCOVA Statistical Analysis	Fig1
7.009	ITT	Posterior Probability of MTR Value Post Lesion relative to Pre Lesion for delta-MTR Lesions Excluding MRI Scans Within 70 Days of Lesion Occurrence with at Least 2 MRI Assessments Pre- and Post-Lesion Based on ANCOVA Statistical Analysis	Fig1
7.010	ITT	Posterior Probability of MTR Value Post Lesion relative to Pre Lesion for gadolinium-enhancing Lesions Excluding MRI Scans Within 70 Days of Lesion Occurrence averaged for each Subject Based on ANCOVA Statistical Analysis	Fig1
7.011	ITT	Posterior Probability of MTR Value Post Lesion relative to Pre Lesion for gadolinium-enhancing Lesions Excluding MRI Scans Within 70 Days of Lesion Occurrence averaged for each Subject with at Least 2 MRI Assessments Pre- and Post-Lesion Based on ANCOVA Statistical Analysis	Fig1
7.012	ITT	Posterior Probability of MTR Value Post Lesion relative to Pre Lesion for delta-MTR Lesions Excluding MRI Scans Within 70 Days of Lesion Occurrence averaged for each Subject Based on ANCOVA Statistical Analysis	Fig1
7.013	ITT	Posterior Probability of MTR Value Post Lesion relative to Pre Lesion for delta-MTR Lesions Excluding MRI Scans Within 70 Days of Lesion Occurrence averaged for each Subject with at Least 2 MRI Assessments Pre- and Post-Lesion Based on ANCOVA Statistical Analysis	Fig1
7.014	ITT	Adjusted Mean Change in MTR Value at each Relative MRI for Gadolinium-Enhancing Lesions Excluding MRI Scans Within 70 Days of Lesion Occurrence from Mixed-Model Repeated Measures Analysis	

Figure No	Pop	Listing Title	Template Reference
7.015	ITT	Posterior Probability of MTR Value Post Lesion relative to Pre Lesion for Gadolinium-Enhancing Lesions Excluding MRI Scans Within 70 Days of Lesion Occurrence Based on MMRM Analysis	Fig1
7.016	ITT	Adjusted Mean Change in MTR Value at each Relative MRI for Gadolinium-Enhancing Lesions Excluding MRI Scans Within 70 Days of Lesion Occurrence with at Least 2 MRI Assessments Pre- and Post-Lesion from Mixed-Model Repeated Measures Analysis	
7.017	ITT	Posterior Probability of MTR Value Post Lesion relative to Pre Lesion for Gadolinium-Enhancing Lesions Excluding MRI Scans Within 70 Days of Lesion Occurrence with at Least 2 MRI Assessments Pre- and Post-Lesion Based on MMRM Analysis	Fig1
7.018	ITT	Adjusted Mean Change in MTR Value at each Relative MRI for delta-MTR Lesions Excluding MRI Scans Within 70 Days of Lesion Occurrence from Mixed-Model Repeated Measures Analysis	
7.019	ITT	Posterior Probability of MTR Value Post Lesion relative to Pre Lesion for delta-MTR Lesions Excluding MRI Scans Within 70 Days of Lesion Occurrence Based on MMRM Analysis	Fig1
7.020	ITT	Adjusted Mean Change in MTR Value at each Relative MRI for delta-MTR Lesions Excluding MRI Scans Within 70 Days of Lesion Occurrence with at Least 2 MRI Assessments Pre- and Post-Lesion from Mixed-Model Repeated Measures Analysis	
7.021	ITT	Posterior Probability of MTR Value Post Lesion relative to Pre Lesion for delta-MTR Lesions Excluding MRI Scans Within 70 Days of Lesion Occurrence with at Least 2 MRI Assessments Pre- and Post-Lesion Based on MMRM Analysis	Fig1
7.022	ITT	Adjusted Mean Change in MTR Value at each Relative MRI for Gadolinium-Enhancing Lesions Averaging lesions for a Subject Excluding MRI Scans Within 70 Days of Lesion Occurrence from Mixed-Model Repeated Measures Analysis	
7.023	ITT	Posterior Probability of MTR Value Post Lesion relative to Pre Lesion for Gadolinium-Enhancing Lesions Excluding MRI Scans Within 70 Days of Lesion Occurrence averaged for each Subject Based on MMRM Analysis	Fig1
7.024	ITT	Adjusted Mean Change in MTR Value at each Relative MRI for Gadolinium-Enhancing Lesions Averaging lesions for a Subject Excluding MRI Scans Within 70 Days of Lesion Occurrence with at Least 2 MRI Assessments Pre- and Post-Lesion from Mixed-Model Repeated Measures Analysis	

Figure No	Pop	Listing Title	Template Reference
7.025	ITT	Posterior Probability of MTR Value Post Lesion relative to Pre Lesion for Gadolinium-Enhancing Lesions Excluding MRI Scans Within 70 Days of Lesion Occurrence averaged for each Subject with at Least 2 MRI Assessments Pre- and Post-Lesion Based on MMRM Analysis	Fig1
7.026	ITT	Adjusted Mean Change in MTR Value at each Relative MRI for delta-MTR Lesions Averaging lesions for a Subject Excluding MRI Scans Within 70 Days of Lesion Occurrence from Mixed-Model Repeated Measures Analysis	
7.027	ITT	Posterior Probability of MTR Value Post Lesion relative to Pre Lesion for delta-MTR Lesions Excluding MRI Scans Within 70 Days of Lesion Occurrence averaged for each Subject Based on MMRM Analysis	Fig1
7.028	ITT	Adjusted Mean Change in MTR Value at each Relative MRI for delta-MTR Lesions Averaging lesions for a Subject Excluding MRI Scans Within 70 Days of Lesion Occurrence with at Least 2 MRI Assessments Pre- and Post-Lesion from Mixed-Model Repeated Measures Analysis	
7.029	ITT	Posterior Probability of MTR Value Post Lesion relative to Pre Lesion for delta-MTR Lesions Excluding MRI Scans Within 70 Days of Lesion Occurrence averaged for each Subject with at Least 2 MRI Assessments Pre- and Post-Lesion Based on MMRM Analysis	Fig1
**Conventional MRI Lesions**			
7.030	ITT	Raw Mean Number of Cumulative Number of New Gadolinium Enhancing Lesions (AES)	FE01
7.031	ITT	Raw Mean Number of Cumulative Number of New Enlarging T2 Lesions (AES)	FE01
7.032	ITT	Raw Mean Number of Cumulative Number of Unique Active Lesions (AES)	FE01
7.033	ITT	Raw Mean Number of Cumulative Number of New Unenhancing T1 Lesions (AES)	FE01
7.034	ITT	Raw Mean Number of Cumulative Number of New Gadolinium Enhancing Lesions Evolving into Black Holes at Week 48 (AES)	FE01
**Conventional MRI Volumes**			
7.035	ITT	Raw Mean Change from Screening in White Matter Volume at each MRI (AES)	FE01
7.036	ITT	Raw Mean Change from Screening in Grey Matter Volume at each MRI (AES)	FE01

Figure No	Pop	Listing Title	Template Reference
7.037	ITT	Adjusted Mean Change from Screening in White Matter Volume at each MRI from Mixed-Model Repeated Measures Analysis (AES Dataset)	
7.038	ITT	Adjusted Mean Change from Screening in White Matter Volume at each MRI from Mixed-Model Repeated Measures Analysis (AES Dataset)	
**CogState**			
7.039	ITT	Adjusted Mean Change from Baseline (90% CI) in CogState Battery Total Score by Visit (MMRM Results)	Fig2
7.040	ITT	Posterior Probability of Change from Baseline in Cogstate Battery Total Score at Week 48	Fig1
7.041	ITT	Adjusted Mean Change from Baseline (90% CI) in CogState Battery Executive Function Composite Score by Visit (MMRM Results)	Fig2
7.042	ITT	Posterior Probability of Change from Baseline in Cogstate Battery Executive Function Composite Score at Week 48	Fig1
7.043	ITT	Adjusted Mean Change from Baseline (90% CI) in CogState Battery Memory Composite Score by Visit (MMRM Results)	Fig2
7.044	ITT	Posterior Probability of Change from Baseline in Cogstate Battery Memory Composite Score at Week 48	Fig1
7.045	ITT	Adjusted Mean Change from Baseline (90% CI) in CogState Battery Attention Composite Score by Visit (MMRM Results)	Fig2
7.046	ITT	Posterior Probability of Change from Baseline in Cogstate Battery Attention Composite Score at Week 48	Fig1
7.047	ITT	Adjusted Mean Change from Baseline (90% CI) in CogState Battery Groton Maze Learning Test Standardised Scores by Visit (MMRM Results)	Fig2
7.048	ITT	Adjusted Mean Change from Baseline (90% CI) in CogState Battery One Back Test Standardised Scores by Visit (MMRM Results)	Fig2
7.049	ITT	Adjusted Mean Change from Baseline (90% CI) in CogState Battery International Shopping List Task – Immediate Recall Task Standardised Scores by Visit (MMRM Results)	Fig2
7.050	ITT	Adjusted Mean Change from Baseline (90% CI) in CogState Battery International Shopping List Task – Delayed Recall Task Standardised Scores by Visit (MMRM Results)	Fig2
7.051	ITT	Adjusted Mean Change from Baseline (90% CI) in CogState Battery One Card Learning Task Standardised Scores by Visit (MMRM Results)	Fig2

Figure No	Pop	Listing Title	Template Reference
7.052	ITT	Adjusted Mean Change from Baseline (90% CI) in CogState Battery Detection Task Standardised Scores by Visit (MMRM Results)	Fig2
7.053	ITT	Adjusted Mean Change from Baseline (90% CI) in CogState Battery Identification Task Standardised Scores by Visit (MMRM Results)	Fig2
**T2 Lesion MTR**			
7.054	ITT	Raw Mean Change from Screening in T2 Lesion MTR Value at each MRI	
7.055	ITT	Adjusted Mean Change from Baseline (90% CI) in T2 Lesion MTR Value by Visit (MMRM Results)	Fig2
7.056	ITT	Posterior Probability of Change from Baseline T2 Lesion MTR Value at Week 48	Fig1

Safety Tables

Table No.	Pop	Table Title	Template Reference
**Adverse Events**			
8.001	ITT	Summary of All Adverse Events by Study Phase	**AE1**
8.002	ITT	Summary of Drug-related Adverse Events by Study Phase	**AE1**
8.003	ITT	Summary of Adverse Events by Maximum Intensity and Study Phase	**AE5**
8.004	ITT	Summary of Adverse Events considered to be Drug-Related by Maximum Intensity and Study Phase	**AE5**
8.005	ITT	Summary of Common Adverse Events Occurring in ≥5% of Subjects in Either Treatment Group.	**AE1**
8.006	ITT	Summary of (Investigator assessed) study medication related Common Adverse Events Occurring in ≥5% of Subjects in Either Treatment Group.	**AE1**
8.007	ITT	Summary of Characteristics of Adverse Events by Study Phase	**ESI1**
8.008	ITT	Summary of Adverse Events by Time of First Occurrence	**AE6**
8.009	ITT	Summary of All Adverse Events Occurring in the Titration Period by GSK239512 Dose	**AE1**
8.010	ITT	Summary of Drug-related Adverse Events Occurring in the Titration Period by GSK239512 Dose	**AE1**

Table No.	Pop	Table Title	Template Reference
8.011	ITT	Summary of Adverse Events considered to be Drug-Related Occurring in the Titration Period by Maximum Intensity GSK239512 Dose	**AE5**
8.012	ITT	Summary of Common Adverse Events Occurring in ≥5% of Subjects Occurring in the Titration Period by GSK239512 Dose	**AE1**
8.013	ITT	Summary of Adverse Events of Special Interest Related to Convulsions by Study Phase	**AE1**
8.014	ITT	Summary of Adverse Events of Special Interest Related to Perceptual Abnormalities by Study Phase	**AE1**
8.015	ITT	Summary of Adverse Events of Special Interest Related to Insomnia/Sleep Disorders by Study Phase	**AE1**
8.016	ITT	Summary of Adverse Events of Special Interest Related to Abnormal Dreams/Nightmares by Study Phase	**AE1**
8.017	ITT	Summary of Adverse Events of Special Interest Related to Mood Symptoms by Study Phase	**AE1**
8.018	ITT	Summary of Adverse Events of Special Interest Related to Feeding and Body Weight Change by Study Phase	**AE1**
8.019	ITT	Summary of Characteristics of Adverse Events of Special Interest Related to Convulsions by Study Phase	**ESI1**
8.020	ITT	Summary of Characteristics of Adverse Events of Special Interest Related to Perceptual Abnormalities by Study Phase	**ESI1**
8.021	ITT	Summary of Characteristics of Adverse Events of Special Interest Related to Insomnia/Sleep Disorders by Study Phase	**ESI1**
8.022	ITT	Summary of Characteristics of Adverse Events of Special Interest Related to Abnormal Dreams/Nightmares by Study Phase	**ESI1**
8.023	ITT	Summary of Characteristics of Adverse Events of Special Interest Related to Mood Symptoms by Study Phase	**ESI1**
8.024	ITT	Summary of Characteristics of Adverse Events of Special Interest Related to Feeding and Body Weight Change by Study Phase	**ESI1**
8.025	ITT	Summary of Adverse Events (AEs) coding to MedDRA HLGT "Sleep disorders and disturbances" by Study Phase	**AE1**
8.026	ITT	Summary of AEs coding to MedDRA HLGT "Depressed mood disorders and disturbances" by Study Phase	**AE1**
8.027	ITT	Summary of AEs coding to MedDRA HLGT "Suicidal and self-injurious behaviours NEC" by Study Phase	**AE1**
8.028	ITT	Summary of Investigator-Reported Serious Adverse Events by Study Phase	**AE1**

Table No.	Pop	Table Title	Template Reference
8.029	ITT	Summary of Adverse Events Leading to Permanent Discontinuation of Study Drug and/or Withdrawal from Study by Study Phase	**AE1**
**Laboratory Data**			
8.030	ITT	Summary of Haematology Laboratory Values	**LB1**
8.031	ITT	Summary of Chemistry Laboratory Values	**LB1**
8.032	ITT	Summary of Change from Baseline in Haematology Laboratory Values	**LB1**
8.033	ITT	Summary of Change from Baseline in Chemistry Laboratory Values	**LB1**
8.034	ITT	Summary of Haematology Laboratory Values outside the Normal Range (F1 flag)	**LB2**
8.035	ITT	Summary of Chemistry Laboratory Values outside the Normal Range (F1 flag)	**LB2**
8.036	ITT	Summary of Haematology Laboratory Values outside the Clinical Concern Range (F3 flag)	**LB2**
8.037	ITT	Summary of Chemistry Laboratory Values outside the Clinical Concern Range (F3 flag)	**LB2**
8.038	ITT	Summary of Changes in Haematology Laboratory Values from Baseline with respect to the Normal Rages (F1 Flag)	**LB4**
8.039	ITT	Summary of Changes in Chemistry Laboratory Values from Baseline with respect to the Normal Rages (F1 Flag)	**LB4**
8.040	ITT	Summary of Changes in Haematology Laboratory Values (using Clinical Concern Ranges)	**LB4**
8.041	ITT	Summary of Changes in Chemistry Laboratory Values (using Clinical Concern Ranges)	**LB4**
8.042	ITT	Summary of Graded LFT Values	LV01
**MS Specific Safety Endpoints**			
8.043	ITT	Summary of Expanded Disability Status Scale (EDSS) Total Score	NS_SAF01
8.044	ITT	Summary of Expanded Disability Status (EDSS): Visual Modified Score	NS_SAF01
8.045	ITT	Summary of Expanded Disability Status (EDSS): Brainstem Functional System Score	NS_SAF01
8.046	ITT	Summary of Expanded Disability Status (EDSS): Pyramidal Function System Score	NS_SAF01
8.047	ITT	Summary of Expanded Disability Status (EDSS): Cerebellar Functional System Score	NS_SAF01

Table No.	Pop	Table Title	Template Reference
8.048	ITT	Summary of Expanded Disability Status (EDSS): Sensory Functional System Score	NS_SAF01
8.049	ITT	Summary of Expanded Disability Status (EDSS): Bladder/ Bowel Modified Functional System Score	NS_SAF01
8.050	ITT	Summary of Expanded Disability Status (EDSS): Cerebral Functional System Score	NS_SAF01
8.051	ITT	Summary of Expanded Disability Status (EDSS): Ambulatory Score	NS_SAF01
8.052	ITT	Summary of Change from Baseline in Expanded Disability Status Scale (EDSS)	NS_SAF04
8.053	ITT	Summary of Sustained Changes in Functional System Scores at Week 48	NS_SAF02
8.054	ITT	Summary of Relapses	NS_SAF03
8.055	ITT	Summary of Relapses by Baseline EDSS Score and Study Phase	NS_SAF03
8.056	ITT	Summary of Neurological Deficits	NS_SAF05
8.057	ITT	Summary of Statistical Analysis of the Relapse Rate during the Treatment Phase	NS_TE02
8.058	ITT	Summary of Statistical Analysis of the Proportion of Subjects Relapsing during the Treatment Phase	NS_SAF06
8.059	ITT	Summary of Statistical Analysis of the Time to First Relapse during the Treatment Phase	NS_SAF07
**Other Safety Endpoints**			
8.060	ITT	Summary of Vital Signs	**VS1**
8.061	ITT	Summary of Change from Baseline in Vital Signs	**VS1**
8.062	ITT	Summary of Vital Signs with respect to the Reference Range and Change from Baseline Criteria	**VS2**
8.063	ITT	Summary of ECG Findings	**EG1**
8.064	ITT	Summary of ECG Values	**EG2**
8.065	ITT	Summary of Change from Baseline in ECG Values	**EG2**
8.066	ITT	Summary of ECG Values of Potential Clinical Concern	**LB2**
8.067	ITT	Summary of Change from Baseline in ECG Values of Potential Clinical Concern	**LB2**
8.068	ITT	Summary of the Number of Subjects within each Category of the Columbia Suicide Severity Rating Scale.	**ECSSR1**

Safety Listings

Listing No	Pop	Listing Title	Template Reference
8.001	R	Listing of All Adverse Events	**AE8**
8.002	R	Relationship of Adverse Events System Organ Classes, Higher Level Group Terms, Preferred terms and Verbatim Text	**AE2**
8.003	R	Listing of Subject Numbers for Individual On-Treatment Adverse Events	**AE7**
8.004	R	Listing of Adverse Events of Special Interest	**AE8**
8.005	R	Listing of HLGT Adverse Events of Special Interest	**AE8**
8.006	R	Listing of Possible Suicidality-Related Adverse Events (Section 1-Section 2)	**PSRAE1**
8.007	R	Listing of Possible Suicidality-Related Adverse Events (Section 3)	**PSRAE2**
8.008	R	Listing of Possible Suicidality-Related Adverse Events (Section 4)	**PSRAE3**
8.009	R	Listing of Possible Suicidality-Related Adverse Events (Section 5-Section 8)	**PSRAE4**
8.010	R	Listing of Fatal Adverse Events	**AE8**
8.011	R	Listing of Non-Fatal Serious Adverse Events	**AE8**
8.012	R	Listing of Additional Information for SAEs	NS_LS01
8.013	R	Listing of All Adverse Events Leading to Permanent Discontinuation of Study Drug and/or Withdrawal from Study	**AE8**
	R		
8.014	R	Listing of Laboratory Data of Potential Clinical Concern	**LB5**
8.015	R	Listing of Laboratory Data for Subjects with Abnormalities of Potential Clinical Concern	**LB5**
8.016	R	Listing of Liver Events in Relation to Time	**LIVER5**
8.017	R	Listing of Liver Events Information for RUCAM Score	**LIVER6**
8.018	R	Listing of Liver Biopsy	**LIVER7**
8.019	R	Listing of Liver Imaging Details	**LIVER8**
8.020	R	Listing of Urinalysis Data	**UR1**
8.021	R	Listing of EDSS Total Score and Functional System Scores	NS_LSAF01

Listing No	Pop	Listing Title	Template Reference
8.022	R	Listing of EDSS Visual FSS Questions	NS_LOV1
8.023	R	Listing of EDSS Brainstem FSS Questions	NS_LOV2
8.024	R	Listing of EDSS Pyramidal FSS Questions - Reflexes	NS_LOV3a
8.025	R	Listing of EDSS Pyramidal FSS Questions - Limbs	NS_LOV3b
8.026	R	Listing of EDSS Pyramidal FSS Questions – Spasticity and Motor Performance	NS_LOV3c
8.027	R	Listing of EDSS Cerebellar FSS Questions	NS_LOV4
8.028	R	Listing of EDSS Sensory FSS Questions	NS_LOV5
8.029	R	Listing of EDSS Bladder/Bowel FSS Questions	NS_LOV6
8.030	R	Listing of EDSS Mental FSS Questions	NS_LOV7
8.031	R	Listing of EDSS Ambulation Questions	NS_LOV8
8.032	R	Listing of Subjects with Relapses	NS_LSAF06
8.033	R	Listing of Details of Relapses (Neurological Deficits)	NS_LSAF07
8.034	R	Listing of Expanded Disability Status (EDSS) Scores at the Time of Relapses	NS_LSAF01
8.035	R	Listing of Steroids administered to Subjects during Relapses	**CM3**
8.036	R	Listing of Vital Signs	**VS4**
8.037	R	Listing of Vital Signs Data for Subjects with Abnormalities of Potential Clinical Concern	**VS4**
8.038	R	Listing of ECG Findings	**EG5**
8.039	R	Listing of ECG Values	**EG3**
8.040	R	Listing of eC-SSRS Suicidal Ideation and Behaviour Data for Completed eC-SSRS Assessments Only	**ECSSRS4**
8.041	R	Listing of eC-SSRS Suicidal Behaviour Details for Completed eC-SSRS Assessments Only	**ECSSRS5**
8.042	R	Listing of Details of Most Severe Suicidal Ideation at Each eC-SSRS Assessment for Completed eC-SSRS Assessments Only	**ECSSRS6**
8.043	R	Listing of eC-SSRS Suicidal Ideation and Behaviour Data for Incomplete eC-SSRS Assessments Only	**ECSSRS4**

Listing No	Pop	Listing Title	Template Reference
8.044	R	Listing of C-SSRS Suicidal Ideation and Behaviour Data for Completed C-SSRS Assessments Only	**CSSRS4**

Safety Figures

Figure No	Pop	Figure Title	Template Reference
8.001	ITT	Dotplot of Most Frequent On-Treatment Adverse Events by Relative Risk (GSK239512 vs placebo)	**AE10**
8.002	ITT	Boxplot of Chemisty Parameters by visit	**LB9**
8.003	ITT	Boxplot of Haematology Parameters by visit	**LB9**
8.004	ITT	Scatter Plot of Maximum On-Treatment Value for each Chemistry Laboratory Parameter versus Baseline	None
8.005	ITT	Scatter Plot of Maximum On-Treatment Value for each Haematology Laboratory Parameter versus Baseline	None
8.006	ITT	Boxplot of Maximum On-Treatment LFTs	**LB10**
8.007	ITT	Scatter Plot of Week 48 EDSS Score versus Baseline EDSS Score	None
8.008	ITT	Kaplan Meier Plot of Time to First Relapse	**TTE10**
8.009	ITT	Boxplot of Change from Baseline in Vital Signs by visit	**LB9**
8.010	ITT	Boxplot of Change from Baseline in QTcB and QTcF	**EG8**

Health Outcomes Tables

Table No.	Pop	Table Title	Template Reference
9.001	ITT	Summary Statistics for MSQoL54 Subscale Scores	HO1
9.002	ITT	Frequency Counts for the MSQoL54 Single-item Measures	HO2
9.003	ITT	Summary Statistics for MSQoL54 Composite Scores	HO1
9.004	ITT	Summary of Changes from Screening in MSQoL54 Composite Scores at Week 48	HO3
9.005	ITT	Summary of the Statistical Analysis of the Change from Screening at Week 48 in MSQoL54 Physical Health Composite Score (AES Dataset)	HO4

Table No.	Pop	Table Title	Template Reference
9.006	ITT	Summary of the Statistical Analysis of the Change from Screening at Week 48 in MSQoL54 Mental Health Composite Score (AES Dataset)	HO4

Health Outcomes Listings

Listing No	Pop	Listing Title	Template Reference
9.001	R	Listing of MSQoL54 Scale Responses	HO5
9.002	R	Listing of MSQoL54 Scale Subscale Scores	HO6
9.003	R	Listing of MSQoL54 Scale Composite Scores	HO7

Pharmacokinetic Tables

Table No.	Pop	Table Title	Template Reference
10.001	PK	Summary statistics for GSK239512 trough plasma concentrations (ng/mL) by week and by dose	BPT responsible
10.002	PK	Summary statistics for GSK239512 plasma concentrations (ng/mL) by nominal time at Week 8 and by dose	BPT responsible
10.003	PK	Summary statistics for GSK239512 average trough plasma concentration used for PK/PD analyses by dose	CPMS to provide individual values to BPT for summarising

Pharmacokinetic Listings

Listing No.	Pop	Table Title	Template Reference
10.001	PK	Listing of individual GSK239512 trough plasma concentrations (ng/mL) by week and by dose	BPT responsible
10.002	PK	Listing of individual GSK239512 plasma concentrations (ng/mL) by nominal time at Week 8 and by dose	BPT responsible

Listing No.	Pop	Table Title	Template Reference
10.003	PK/PD	Listing of individual GSK239512 average trough plasma concentration used for PK/PD analyses by dose	CPMS to provide individual values to BPT for summarising

Pharmacokinetic Figures

Figure No.	Pop	Table Title	Template Reference
10.001	PK	Individual GSK239512 trough plasma concentrations (ng/mL) by week and by dose.	CPMS responsible.
10.002	PK	Individual GSK239512 plasma concentrations (ng/mL) by nominal time at Week 8 and by dose	CPMS responsible
10.003	PK	Box plot of GSK239512 trough concentrations (ng/mL) by week and by dose	CPMS responsible

Pharmacokinetic/Pharmacodynamic Figures

Figure No.	Pop	Table Title	Template Reference
10.001	PK/PD	MTR primary 1) versus average trough concentration	CPMS responsible.
10.002	PK/PD	MTR primary 2) versus average trough concentration	CPMS responsible

16.2. Data Display Specifications

Template Shells

477 Study

16.3. Laboratory Normal Ranges and Potential Clinical Concern Ranges

Lab Test Code	Algorithm Type	Low Clinical Concern Value	High Clinical Concern Value
ALB_PLC	A	26.0000	65.0000
ALP_PLC	+NR	.	3.0000
ALT_PLC	+NR	.	3.0000
AST_PLC	+NR	.	3.0000
BILT_PLC	A	.	44.0000
CA_PLC	A	1.8962	2.7000
CRT_PLC	A	.	176.8000
EOS_BLC	A	.	1.0000
GGT_PLC	+NR	.	3.0000
GLUC_PLC	A	2.7900	12.0000
HB_BLC	+NR	0.8500	1.1500
HCT_BLQ	+NR	0.8500	1.1500
K_PLC	A	3.0000	6.0000
LYMPH_BLC	A	0.6000	5.0000
MONO_BLC	A	.	2.0000
NA_PLC	A	127.0000	151.0000
NEUT_BLC	A	1.5000	.
PLT_BLC	A	100.0000	600.0000
RETIC_BLC	+NR	0.7500	1.2000
TP_PLC	+NR	0.8000	1.2000
WBC_BLC	A	3.0000	16.0000
BASO_BLC	+NR		2
BILD_PLC	+NR		1.5
CK_PLC	+NR		2
LDH_PLC	+NR		2
MCHC_BLC	+NR	0.75	1.2
MCH_BLC	+NR	0.75	1.2
MCV_BLV	+NR	0.75	1.2
RBC_BLC	+NR	0.75	1.2
UREA_BLC	+NR	0.8	2
Note: A=Absolute Value; +NR=Multiple of Normal range			
